# Integrative transcriptome imputation reveals tissue-specific and shared biological mechanisms mediating susceptibility to complex traits

**DOI:** 10.1038/s41467-019-11874-7

**Published:** 2019-08-23

**Authors:** Wen Zhang, Georgios Voloudakis, Veera M. Rajagopal, Ben Readhead, Joel T. Dudley, Eric E. Schadt, Johan L. M. Björkegren, Yungil Kim, John F. Fullard, Gabriel E. Hoffman, Panos Roussos

**Affiliations:** 1 Department of Psychiatry, Pamela Sklar Division of Psychiatric Genomics and Friedman Brain Institute, Icahn School of Medicine at Mount Sinai, New York, NY 10029 USA; 2 Department of Genetics & Genomic Sciences and Institute for Genomics and Multiscale Biology, Icahn School of Medicine at Mount Sinai, New York, 10029 NY USA; 3 Department of Biomedicine, Aarhus University, 8000 Aarhus, Denmark; 4 The Lundbeck Foundation Initiative for Integrative Psychiatric Research (iPSYCH), Aarhus University, 8000 Aarhus, Denmark; 5 Centre for Integrative Sequencing (iSEQ), Aarhus University, 8000 Aarhus, Denmark; 6 The Biodesign Institute, Arizona State University, Tempe, AZ 85281 USA; 7 Clinical Gene Networks AB, 114 44 Stockholm, Sweden; 8 Vascular Biology Unit, Department of Medical Biochemistry and Biophysics, Karolinska Institutet, 171 77 Stockholm, Sweden; 9 Department of Physiology, Institute of Biomedicine and Translation Medicine, University of Tartu, 50411 Tartu, Estonia; 10 Mental Illness Research Education and Clinical Center (MIRECC), James J. Peters VA Medical Center, Bronx, 10468 NY USA

**Keywords:** Data integration, Machine learning, Genetic association study, Drug development

## Abstract

Transcriptome-wide association studies integrate gene expression data with common risk variation to identify gene-trait associations. By incorporating epigenome data to estimate the functional importance of genetic variation on gene expression, we generate a small but significant improvement in the accuracy of transcriptome prediction and increase the power to detect significant expression-trait associations. Joint analysis of 14 large-scale transcriptome datasets and 58 traits identify 13,724 significant expression-trait associations that converge on biological processes and relevant phenotypes in human and mouse phenotype databases. We perform drug repurposing analysis and identify compounds that mimic, or reverse, trait-specific changes. We identify genes that exhibit agonistic pleiotropy for genetically correlated traits that converge on shared biological pathways and elucidate distinct processes in disease etiopathogenesis. Overall, this comprehensive analysis provides insight into the specificity and convergence of gene expression on susceptibility to complex traits.

## Abstract (web summary)

PrediXcan is a widely used gene expression imputation method that links genetic variants to gene expression. Here, the authors develop EpiXcan which leverages epigenetic annotations to inform transcriptomic imputation and further use the obtained gene-trait associations for computational drug repurposing.

## Introduction

Despite the recent success of genome-wide association studies (GWASs) in furthering our understanding of the genetic basis of disease, the mechanisms through which many of the identified risk variants act remain largely unknown^1^. Disease-associated risk variants are highly enriched in *cis* regulatory elements (CREs), including promoters and enhancers^2,3^ and increasing evidence suggests that they affect the regulation of gene expression^2–6^. Multiple computational methods have been developed to perform transcriptome-wide association studies (TWASs) linking risk variants with differential gene expression^7–11^. For instance, using the summary data-based Mendelian randomization method, Zhu and colleagues conducted a TWAS for complex traits by integrating eQTL and GWAS summary data^9^. However, the field is increasingly favoring transcriptomic imputation methods as the basis of TWAS applications, as they enable feature-centered modeling of the combined effect of multiple *cis*-SNPs (SNPs in proximity to the transcription start site) on transcription. The two most widely used methods are PrediXcan and FUSION. Gusev et al. developed the latter method and were the first to apply it to GWAS summary statistics to explore genetic mechanisms for complex traits^11^. On the other hand, PrediXcan^12^ is the first, and most widely used, transcriptomic imputation method for individual genotypes that was adapted for use with GWAS summary statistics and, so far, it outperforms similar methods^13^. Briefly, PrediXcan uses elastic net (ENet) regression models, trained in a reference transcriptome, to impute gene expression. The models use a set of *cis*-SNPs as linear predictors of gene expression. The imputed expressions are then correlated with the phenotype of interest to identify gene-trait associations (GTAs). The generated trait-associated imputed transcriptomes can also be leveraged for diverse downstream applications such as the identification of candidate compounds, for which we have reference transcriptomic data, that are predicted to reverse trait-specific, genetically driven, gene expression changes^14^. These downstream applications depend on the prediction accuracy of the genetically regulated expression (GReX) and, thus, any improvements in the transcriptomic imputation performance would translate to higher confidence in the GReX-based drug repositioning predictions.

Here, we present EpiXcan, a method that increases prediction accuracy in transcriptome imputation by integrating epigenetic data to model the prior probability that a SNP affects transcription. EpiXcan specifically leverages annotations derived from the Roadmap Epigenomics Mapping Consortium (REMC) that integrates multiple epigenetic assays, including DNA methylation, histone modification and chromatin accessibility^15^. The rationale of our approach is that SNPs within CREs are more likely to be functionally relevant^16^. We utilize 14 large-scale transcriptome datasets of genotyped individuals to train prediction models and integrate with 58 complex traits and diseases to define significant GTAs. GTAs exhibit significant enrichment for relevant biological pathways and known genes linked to trait-related phenotypes in humans and mice. Imputed transcriptomic changes are used to identify known compounds that can normalize genetically driven expression perturbations. Chemogenomic enrichment analyses are performed and an agnostic approach is proposed to validate drug predictions. Pairwise trait analysis identifies genes that exhibit agonistic pleiotropy for genetically correlated traits that converge on shared biological pathways. Finally, bi-directional regression analysis identifies putative causal relationships among traits. Overall, our analysis provides insight into the specificity and convergence of gene expression mediating the genetic risk architecture underlying susceptibility to complex traits and diseases.

## Results

### EpiXcan outperforms PrediXcan

Since TWAS is limited to genes that can be accurately predicted from genotype data, increasing prediction accuracy can increase the scope and power of analyses. Here, we integrate biologically relevant data in a single framework to improve performance of gene expression prediction. The overall schematic of EpiXcan is shown in Supplementary Fig. 1. Briefly, EpiXcan leverages epigenetic annotation to inform transcriptomic imputation by employing a three-step process (see Methods and Supplementary Methods): (1) estimate SNP priors that reflect the likelihood of a SNP having a regulatory role in gene expression based on a Bayesian hierarchical model^17^ that integrates epigenomic annotation^15^ and eQTL summary statistics for *cis*-SNPs (SNPs located ±1 Mb from the transcription start site of the gene); (2) rescale the SNP priors to penalty factors by employing an adaptive mapping approach; and (3) use the genotypes and penalty factors in weighted elastic net to perform gene expression prediction.

Using simulated data, we apply EpiXcan and PrediXcan to train prediction models and estimate the adjusted cross-validation R-squared (*R*^*2*^_CV_), which is the correlation between the predicted and observed expression levels during the nested cross validation. Although the actual *R*^*2*^_CV_ achieved by both methods is generally low, in all simulated scenarios, EpiXcan improves the average *R*^*2*^_CV_ compared to PrediXcan models (all *p* values ≤7 × 10^−10^ based on one-sample sign test; Supplementary Fig. 2). We then train prediction models by applying EpiXcan and PrediXcan in 14 RNAseq datasets, derived from dorsolateral prefrontal cortex (DLPFC) from the CommonMind Consortium (CMC)^18^, seven tissues from Stockholm-Tartu Atherosclerosis Reverse Network Engineering Task (STARNET)^19^ and six tissues from GTEx^20^ (Supplementary Table 1). We compare the performance of EpiXcan with PrediXcan by considering the delta value (EpiXcan minus PrediXcan) of two metrics: (1) cross-validation *R*^2^ (*R*^*2*^_CV_) within each tissue and (2) predictive performance *R*^2^ (*R*^*2*^_PP_), estimated based on Pearson’s correlation between predicted and observed expression in an independent dataset of a relevant tissue. Positive delta values indicate that EpiXcan has higher prediction performance compared to PrediXcan.

**Fig. 1:**
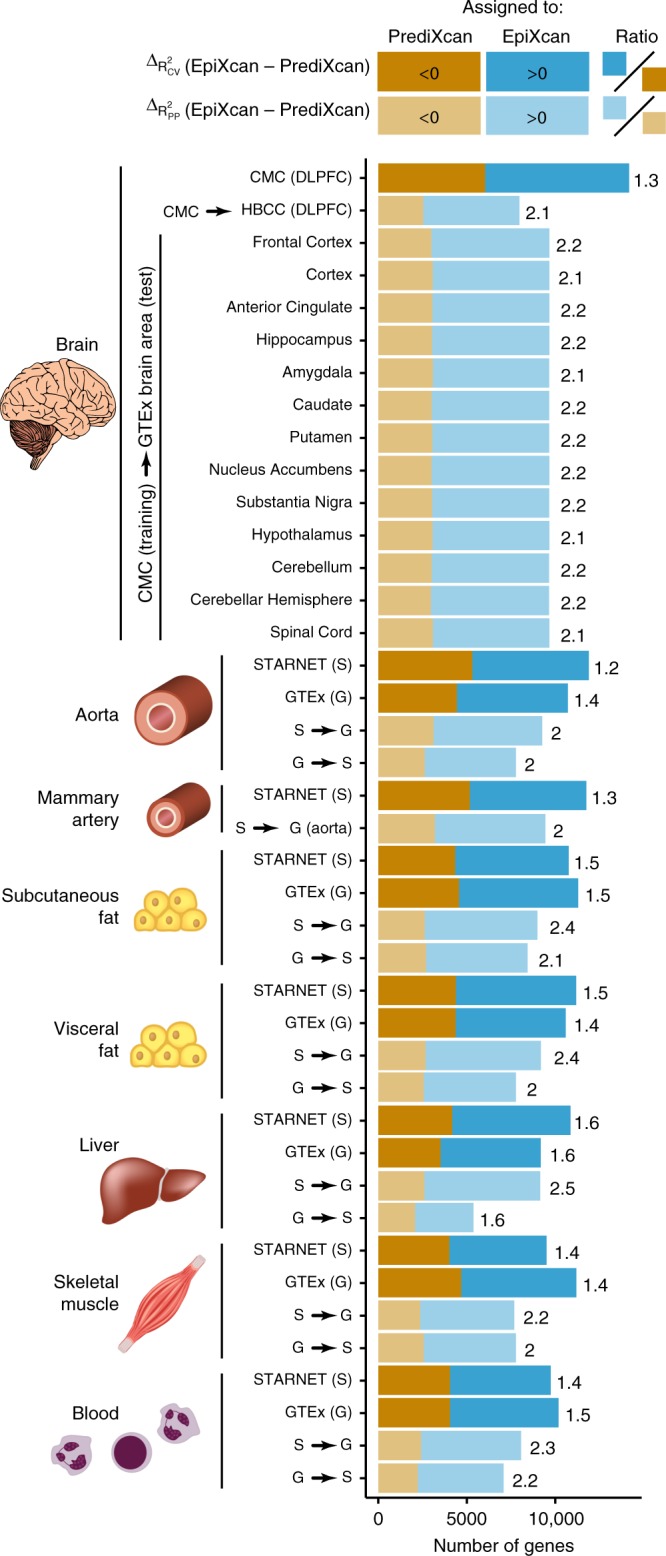
Comparison of prediction performance between EpiXcan and PrediXcan. EpiXcan and PrediXcan models are trained across multiple tissues that include: brain, aorta, mammary artery, subcutaneous fat, visceral fat, liver, skeletal muscle, and blood by leveraging 14 datasets from CMC, STARNET and GTEx. The difference in training performance between EpiXcan and PrediXcan models is compared using the adjusted cross validation *R*^2^ (*R*^*2*^_CV_) metric. The 14 models are further assessed by estimating the predictive performance (*R*^*2*^_PP_) in independent datasets; the training dataset is shown before the arrow and the test dataset after the arrow (G = GTEx and S = STARNET). For a given dataset, we compare the *R*^*2*^_CV_ and *R*^*2*^_PP_ by estimating the delta value of EpiXcan minus PrediXcan for each gene. Positive and negative delta values indicate genes with higher predictive performance in EpiXcan and PrediXcan, respectively. These genes are assigned as “EpiXcan” and “PrediXcan” and counts are shown as barplots. The number on the right indicates the ratio of “EpiXcan” assigned gene counts divided by “PrediXcan” counts. Across all datasets, the ratios are higher than 1 indicating that EpiXcan outperforms PrediXcan. *p* value from one-sample sign test indicates that the shift of the delta *R*^*2*^_CV_ and *R*^*2*^_PP_ values is greater than zero (All *p* values < 9 × 10^−16^)

Across all datasets, EpiXcan improves the average *R*^*2*^_CV_ compared to PrediXcan (all *p* values ≤ 9 × 10^−16^ based on one-sample sign test; Fig. 1**;** Supplementary Figs. 3, 4**;** Supplementary Data 1). We predict 4.6% more genes (pairwise Wilcoxon test *p* value = 6.10 × 10^−5^) with *R*^*2*^_CV_ *>* 0.01 using EpiXcan (average number of genes across tissues is 10,181) compared to PrediXcan (average number of genes across tissues is 9760). To obtain the second metric, *R*^*2*^_*PP*_, we train prediction models in the training dataset, which are then used to predict expressions in the test dataset. Across all datasets, EpiXcan improves the average *R*^*2*^_PP_ compared to PrediXcan (all *p* values < 9 × 10^−16^ based on one-sample sign test; Fig. 1**;** Supplementary Figs. 5–7**;** Supplementary Data 2). Importantly, the ratios of genes predicted more effectively by EpiXcan are higher in the independent dataset evaluation (*R*^*2*^_PP_) than in the cross-validation (unpaired *t*-test, *p* value = 3.3 × 10^−17^) (Fig. 1), suggesting that the adaptive rescaling of the penalty factors during model training does not result in significant overfitting that could affect the external validity of the models. Overall, compared to PrediXcan, EpiXcan has improved predictive performance and identifies more genes that can be used for TWAS.

In addition, we compare EpiXcan to recently developed predictive methods such as the Bayesian sparse linear mixed model (BSLMM)^21^ and the Dirichlet process regression (DPR) method^22^ (Methods**;** Supplementary Methods). EpiXcan outperforms BSLMM and DPR in transcriptomic imputation, both in cross-validation and in independent datasets (all *p* values < 7 × 10^−16^) while having on average, depending on the method, from 0.63× to ~240× the computing speed (Supplementary Fig. 8).

### EpiXcan informs better gene-trait associations

We apply EpiXcan and PrediXcan prediction models from 14 tissues (Supplementary Table 1) in 58 complex traits (Supplementary Data 3) and examine their performance based on four criteria: the number of GTAs that are: (1) significant after multiple testing correction, (2) positioned outside the GWAS loci (3) unique (i.e., genes identified only by one method), and (4) enriched for clinically relevant genes.

**Fig. 2:**
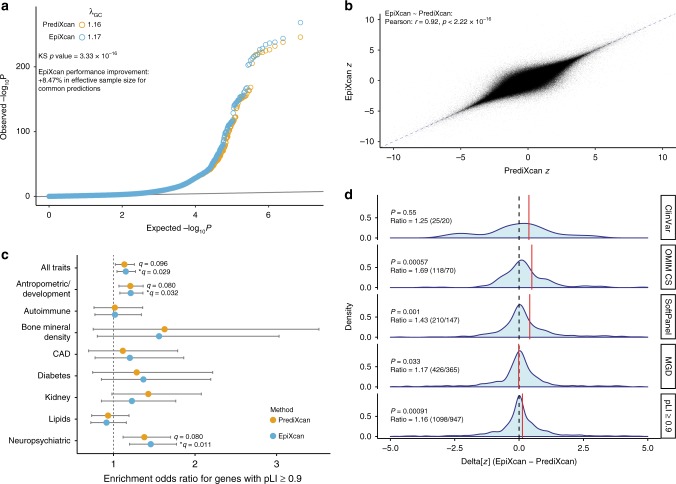
Comparison of gene-trait associations between EpiXcan and PrediXcan. **a** EpiXcan and PrediXcan pairwise Wilcoxon test *p* value distributions for all gene-trait associations. Quantile-quantile (QQ) plot of the *p* values for all gene-trait associations show a significant, albeit modest, shift to the left. The genomic inflation factor (*λ*) is slightly higher for EpiXcan than PrediXcan (1.17 and 1.16). The two distributions are significantly different (Kolmogorov-Smirnov test *p* value is 3.3 × 10^−16^) and EpiXcan achieves an 8.47% improvement in effective sample size for common predictions based on *χ*^2^ test percentage improvement. **b** EpiXcan and PrediXcan have a high correlation of gene-trait association *z* scores. Scatter plot of EpiXcan and PrediXcan *Z* values, Pearson *r* = 0.92 and Spearman *ρ* = 0.91, *p* value < 2.22 × 10^−16^ for both. Only *z* values between −10 and 10 are plotted. The dotted blue line corresponds to *y* *=* *x*. **c** Gene set enrichment analysis (GSEA) for extremely loss-of-function intolerant (pLI ≥ 0.9) genes. Odds ratio with 95% CI are plotted for combined gene-trait associations from all traits and trait categories for enrichment in genes with pLI ≥ 0.9 (* for *q* value < 0.05). For all pLI decile bins enrichment refer to Supplementary Data 4. **d** EpiXcan has more power than PrediXcan to detect expression changes of trait-specific, clinically significant genes. These density plots depict the distribution of the Δ[*z*] (EpiXcan − PrediXcan) values for all gene-trait associations that are significant from either EpiXcan or PrediXcan. *P* value is from one sample sign test. Ratio is the number of Δ[*z*] measurements in favor of EpiXcan to that of PrediXcan. The red lines correspond to the mean of each distribution

EpiXcan has more power to detect GTAs than PrediXcan (Kolmogorov-Smirnov *p* value is 3.3 × 10^−16^, Fig. 2a) and achieves an 8.47% average increase in *χ*^2^ statistic for GTAs where *χ*^2^ ≥ 1 by both methods (*n* = 1,077,801, Mann–Whitney *U* test *p* value < 2.2 × 10^−16^). Since statistical power is linearly related to the *χ*^2^ statistic, this corresponds to EpiXcan producing an 8.47% increase in effective sample size. Consequently, we observe a 9.6% increase (*n* = 1202) in the significant GTAs at 0.01 false discovery rate (FDR)^23^ using EpiXcan (*n* = 13,724) compared to PrediXcan (*n* = 12,522). One advantage of PrediXcan/EpiXcan methods is that they identify genes within loci that did not reach genome-wide significance *(p* value < 5 × 10^−8^) in GWASs. We detect an 18.3% increase (one sample sign test *p* value = 3.6 × 10^−7^) in the GTAs using EpiXcan (mean of 25.4) compared to PrediXcan (mean of 21.5) (Supplementary Fig. 9). The largest difference is observed for height (EpiXcan = 168, PrediXcan = 134), followed by schizophrenia (EpiXcan = 119, PrediXcan = 104) (Supplementary Fig. 10a). The overwhelming majority of the most significant SNPs for these genes identified by both methods have GWAS *p* values within the interval (5 × 10^−8^, 10^−3^) and are more likely to be within the interval (5 × 10^−8^, 10^−5^) when adjusted for the *p* value distribution of LD-independent genomic regions for each GWAS (Supplementary Fig. 10b). They thus represent ‘borderline’ GWAS results that one might expect to be identified as larger studies become available. Similarly, EpiXcan detects 9.95% more GTAs (one-sample sign test *p* value = 0.015) that are not identified by MAGMA gene analysis^24^ compared to PrediXcan (Supplementary Fig. 9).

For any given tissue and trait, we find high correlation of GTA *z* scores between EpiXcan and PrediXcan (Pearson’s correlation *r* *=* *0.92*) (Fig. 2b), although unique associations are observed for each method. We identify 79.9% (*n* = 327) more unique genes in EpiXcan (*n* = 788) than PrediXcan (*n* = 461) (Supplementary Fig. 11), due to either a lack of a prediction model for a specific gene and/or tissue or insufficient statistical power using PrediXcan models. For example, using the waist-adjusted BMI trait and prediction models from STARNET subcutaneous adipose tissue, overall, we observe high correlation between EpiXcan and PrediXcan genes (Pearson’s *r* *=* 0.83) (Supplementary Fig. 12). Interestingly, EpiXcan identifies 7 genes (*PPP2R5A*, *ALAS1*, *HOXC8*, *PIEZO1*, *SCD*, *PARP3*, and *EYA1*) that are not detected by PrediXcan, even if we test across all tissue-specific models. *SCD* (stearoyl–CoA desaturase) is of particular interest, as it encodes an enzyme that catalyzes a rate-limiting step in the synthesis of unsaturated fatty acids (mainly oleate and palmitoleate); knocking out the *SCD* ortholog in the mouse results in reduced body adiposity and resistance to diet-induced weight gain^25^. Accordingly, EpiXcan predicts that upregulated *SCD* gene expression is associated with increased waist-adjusted BMI.

To more broadly compare the unique GTAs identified by EpiXcan or PrediXcan, we wanted to see whether they exhibit similar colocalization properties. Several methods (e.g., HEIDI post-SMR^9^, COLOC^7^, eCAVIAR^26^) make use of local LD patterns in an attempt to distinguish: (1) pleiotropy-driven (causal variants affecting both phenotype and gene expression) and causality-driven (causal variants affecting phenotype via gene expression) GTAs from (2) linkage-driven GTAs (one causal variant affecting gene expression and a second causal variant affecting the phenotype in LD) which can lead to misinterpretation of TWAS-derived GTAs. No method can provide perfect separation of pleiotropy and linkage but, as shown for S-PrediXcan^27^, HEIDI analysis is moderately to highly concordant with COLOC’s classification, and PrediXcan performs favorably when compared to other methods. Thus, we utilize our HEIDI post-SMR analysis^5^ to identify the proportion of genes with good colocalization properties uniquely identified by either study (Methods) and find no difference between EpiXcan and PrediXcan (*χ*^2^ test *p* value = 0.14, Supplementary Note 1).

### EpiXcan uncovers more clinically relevant genes

We perform a series of gene set enrichment analyses (GSEA) to determine how well EpiXcan can uncover clinically relevant genes and molecular pathways compared to PrediXcan. For this, we employ five categories of datasets: (1) ExAC gene pLI (probability of loss-of-function intolerance) dataset^28^, (2) ClinVar dataset—pathogenic or likely pathogenic genes in the ClinVar database^29^, (3) OMIM CS dataset—genes in OMIM with phenotypes in the clinical synopsis (CS) section^30^, (4) SoftPanel dataset—custom gene panels for our traits created with SoftPanel^31^ based on ICD-10 classification and keyword queries (underlying knowledge base is OMIM but gene panel creation is more integrative), and (5) MGD dataset—mouse orthologs of human genes associated with mouse strain-specific phenotypes^32^. GTAs from both PrediXcan and EpiXcan exhibit enrichment for genes that are associated with the traits in the above datasets (Supplementary Fig. 13).

Transcripts identified by EpiXcan (*q* value = 0.029), but not by PrediXcan (*q* value = 0.096), are enriched for genes that are extremely loss-of-function intolerant (pLI ≥ 0.9) (Fig. 2c). More specifically, we find significant enrichment of pLI genes with neuropsychiatric (*q* value = 0.012, known association^33,34^) and anthropometric/development (*q* value = 0.032) related traits (Supplementary Data 4). Unlike pLI, for all other gene sets (ClinVar, OMIC CS, SoftPanel, MGD), we define and test for enrichment only for that specific trait. For example, for autism, we generate a gene list from the significant autism-specific GTAs from all tissues for each method. We then perform GSEA for genes in the ClinVar database that are reported to be associated with autism. In so doing, we find that, overall, EpiXcan has more power than PrediXcan to identify clinically relevant genes (Fig. 2d), including those that are more likely to belong to more than one dataset (pLI, ClinVar, OMIC CS, SoftPanel, MGD) (Supplementary Fig. 14).

In conclusion, TWAS across 58 traits shows that, compared to PrediXcan, EpiXcan has more power to detect significant genes, including unique associations, which are indispensable for life and clinically significant. In the following section, we further explore the EpiXcan-derived GTAs, in terms of: (1) per-tissue contribution of significant genes, (2) gene-set enrichment analysis, (3) computational drug repurposing analysis, and (4) genes shared within, and across, different disease categories.

### Tissues differentially contribute GTAs

**Fig. 3:**
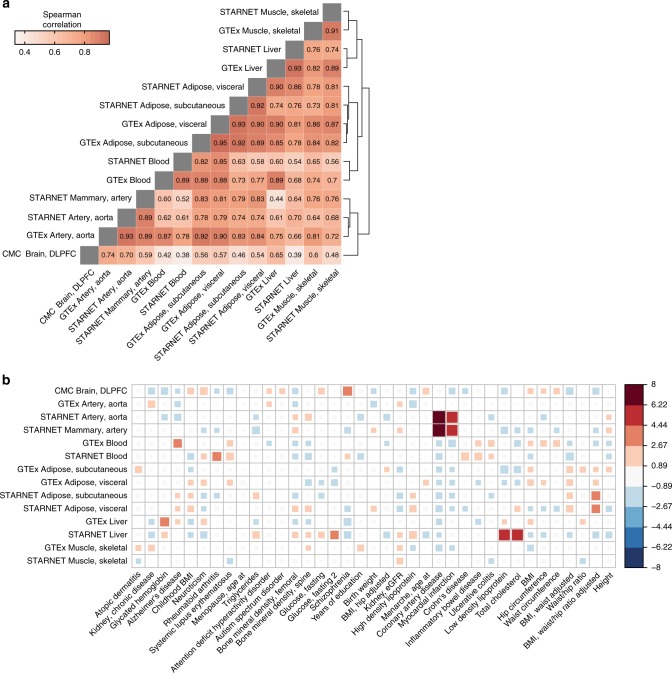
Contribution of GWAS and tissues to gene-trait associations. **a** Correlation of genetically regulated expression imputed for different tissues (pooled GTAs for all traits). Correlation is calculated for significant imputed expression changes with the Spearman method. Dendrogram on the right edge is shown from Ward hierarchical clustering. **b** Enrichment of tissue-specificity of significant EpiXcan GTAs compared to a null model, where each tissue contributes equally (Pearson’s *χ*^2^ test *p* value = 2.7 × 10^−8^). Statistically, the enrichment is the Pearson standardized residual for each tissue-trait pair from the *χ*^2^ test. Box size and color indicate enrichment (red) or depletion (blue) for each tissue-trait pair. Only traits with expected frequency of more than 1 significant gene-trait association for each tissue model are evaluated as per Pearson’s *χ*^2^ test requirements. Tissues and traits are ordered based on Ward hierarchical clustering. Right-hand side panel indicates tissue-specificity enrichment score

In this study, we employ 3 different training cohorts to generate 14 predictive models for 8 tissue homogenate types and use the predictive models to impute tissue-specific transcriptomes across 58 GWASs. By pooling together imputed transcriptomes for each tissue from all traits, we first determine the robustness of our method by examining the *z* score correlation for similar tissues within and across cohorts. As expected, predictions are highly correlated when EpiXcan models are trained in (1) different cohorts (GTEx and STARNET) predicting the same tissue (Spearman’s *ρ*: 0.89–0.93) and (2) the same cohort predicting similar tissues (Spearman’s *ρ*:0.89 when comparing aorta with mammary artery, and 0.92–0.95 when comparing visceral with subcutaneous adipose tissues) (Fig. 3a). In contrast, unrelated tissues, such as blood and brain, exhibit only moderate correlation (Spearman’s *ρ* 0.38–0.42).

Tissue-specificity of GTAs can be used to prioritize biologically relevant tissues for each disease. In contrast to a null model of no trait-associated tissue specificity, significant EpiXcan GTAs are statistically enriched for particular tissues (Pearson’s *χ*^2^ test *p* value = 2.7 × 10^−8^, Fig. 3b). For example, we find a higher number of contributions than expected from brain tissue in schizophrenia and from blood in inflammatory bowel diseases, which is concordant with previous SMR analysis^5^. This occurs despite the observation that largely similar numbers of GTAs are obtained irrespective of tissue source (Supplementary Fig. 15).

For 48 of the traits, more than 50% of the associated genes are found in only one tissue (Supplementary Fig. 16) and a large proportion (32.98 ± 17.36%; mean ± SD) of these unique GTAs come from the highest contributing tissue type (Supplementary Fig. 17). A few examples of top tissue type contributors for unique GTAs are as follows; schizophrenia: brain tissue (30.34%, CMC), myocardial infarction and coronary artery disease: arterial tissue (33.33% and 31.88% respectively, STARNET aorta and mammary artery and GTEx aorta), systemic lupus erythematosus: blood (38.89%, STARNET and GTEx blood), most lipid traits: liver (24.06–26.43%, STARNET and GTEx liver). Besides tissue relevance, cohort size and tissue dissimilarity explain 52% of the variation in the number of unique GTAs contributed by different tissues (Supplementary Fig. 18, multiple linear regression model, *p* value = 0.007). This indicates that additional GTAs will be uncovered with increased sample size of gene expression datasets in disease-relevant tissues.

### Biological relevance of gene-trait associations

High confidence GTAs (observed *p* value vs. expected *p* value) for a given trait get progressively enriched for genes that are more directly implicated in the pathogenesis of diseases with trait-relevant phenotypes. In databases (ClinVar and OMIM) where mostly large effect mutations are cataloged, we observe this progressive increase in enrichment (as indicated by *λ*, Supplementary Fig. 19), starting with genes that are associated with trait-relevant clinical signs, even if those clinical signs are not the primary symptoms of the disorder (OMIM CS: *λ* = 1.29, *p* value = 6.17 × 10^−14^). Next, we see enrichment for genes that are driving similar disorders based on ICD10 classification grouping, or phenotype descriptive terms (SoftPanel: *λ* = 1.36, *p* value < 2.22 × 10^−16^). Finally, we observe the highest enrichment for those genes that are directly driving trait-relevant phenotypes (ClinVar: *λ* = 1.83, *p* value = 7.07 × 10^−14^). We also observe enrichment (*λ* = 1.21, *p* value = 1.69 × 10^−13^) for mouse orthologs that produce mouse phenotypes in the same phenotypic category as the relevant human trait.

We perform gene-set enrichment analysis for traits with more than 10 significant GTAs (43 out of 58 traits) to determine if the associated genes can be mapped to biological processes (Supplementary Data 5). After FDR adjustment, 74 highly enriched pathways are obtained with *p* values < 1.70 × 10^−5^ (corresponds to *q* < 0.05). Significantly associated genes are enriched for biological processes relevant to trait pathophysiology. For instance, the enriched pathways for elevated total cholesterol and triglycerides are involved in sterol and lipid homeostasis, as well as lipoprotein digestion, mobilization, and transport. Similarly, for atopic dermatitis the significantly enriched pathway modulates the rate or extent of water loss from an organism via the skin. In addition, genes associated with mineral density of the femoral bone demonstrate a high enrichment for a pathway that positively regulates cartilage development.

### GReX-based computational drug repurposing

**Fig. 4:**
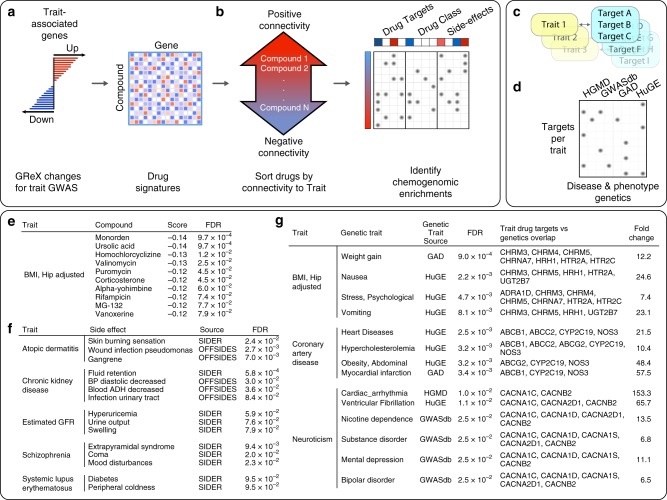
Leveraging gene-trait associations for computational drug repurposing. **a** Trait-associated genes are used to sort a library of drug induced gene expression signatures according to their connectivity with the trait. GReX: genetically regulated expression. **b** A secondary enrichment analysis on this drug list identifies pharmacological features that are over-represented at the extreme ends of the sorted list, thus presenting a chemogenomic view of the trait. **c** Drug targets linked with each trait (FDR < 0.1) are then (**d**) compared with risk loci genes for a range of diseases or phenotypes (FDR < 0.1). **e** Top 10 compounds predicted to normalize the expression of “Hip adjusted BMI” associated genes. **f** Subset of side-effect enrichments for phenotypically related traits. **g** Subset of traits with associated drug targets that are enriched for risk associated genes sets with phenotypically related traits

Computational drug repurposing (CDR) offers a systematic approach for relating disease and drug-induced states towards the goal of identifying indications for existing therapeutics^35^. We perform a computational screen against a library of 1309 drug-induced transcriptional profiles^36^ to identify small molecules capable of perturbing the expression of our identified trait-associated genes (Fig. 4a). For each trait/compound pair, we calculate a signed connectivity score^36^, which summarizes the transcriptional relationship between each trait and drug signature, thus identifying drugs that might be predicted to “normalize” the gene-trait signature, as well as those expected to induce a “disease-like” state (Fig. 4b-d, Supplementary Data 6). Figure 4e provides example compounds predicted to regulate the expression of genes associated with the “Hip circumference adjusted BMI” trait. This list includes drugs under investigation for treatment of obesity, including ursolic acid, which is reported to increase skeletal muscle and brown fat while reducing diet-induced obesity^37^.

To explore the higher-level biological context of trait/compound associations, we perform a chemogenomic enrichment analysis to determine whether drugs that regulate particular sets of trait-associated genes might share pharmacological features, such as drug targets, drug classes, side-effects and drug indications (Fig. 4b, Supplementary Data 6). We find multiple significant (FDR < 0.1) chemogenomic trends, including enrichment with phenotypically related side-effects (Fig. 4f), supporting the potential for these compounds to perturb trait-related molecular networks.

We hypothesized that, in general, trait-associated drug targets would connect to risk-associated genes for phenotypically related diseases^38^. To evaluate this, we identify referenced^39^ and predicted^40^ drug targets that are enriched (FDR < 0.1) among compounds that modulate the signature of each trait. We identify ≥1 drug target enrichment, for 53 of the traits considered, and ≥3 drug targets for 40 traits (Supplementary Data 6). We then perform a further gene set analysis on the targets associated with each trait, focusing on disease risk genetic resources that might implicate phenotypes that could then be related to the traits considered within this study. We identify several significant overlaps (FDR < 0.1) between trait-associated targets and phenotypically related disease risk gene sets (Fig. 4g, Supplementary Data 6). For example, drug targets enriched among compounds that perturb genes associated with “Hip circumference adjusted BMI” are enriched for risk genes for weight gain, nausea, and psychological stress, and drug targets enriched among compounds that perturb “Coronary Artery Disease” associated genes are enriched for risk genes for heart disease, hypercholesterolemia, abdominal obesity, and myocardial infarction.

Towards an objective assessment of the CDR pipeline performance, we compare the CDR predictions with known physician-curated indications for our traits (Supplementary Note 1) that fall into four groups of increasingly perceived efficacy: (1) non-indication: a drug that neither therapeutically changes the underlying or downstream biology nor treats a significant symptom of the disease, (2) symptomatic: a drug that treats a significant symptom of the disease, (3) FDA-approved for the trait, and (4) disease modifying: a drug that therapeutically changes the underlying or downstream biology of the disease. Compounds that are predicted to normalize the gene-trait signature demonstrate progressive enrichment for higher indication levels, whereas compounds that are expected to induce a “disease-like” state show a progressive depletion (Supplementary Fig. 20a). When only considering known disease modifying and non-indication compounds for our traits, compounds that are predicted to normalize the gene-trait signature are more likely to be disease modifying (odds ratio 11.37, Barnarnd’s unconditional test *p* value = 0.006, considering only our CDR predictions with *p* value < 0.3, Supplementary Fig. 20b). In addition, the chemogenomic enrichment for drug indications is also able to identify several cases where compounds predicted to normalize the gene-trait signature are enriched for compounds indicated to treat the trait’s comorbidities, e.g. (1) compounds that would reverse the “current versus former smoking” trait are enriched for compounds indicated for congestive heart failure and increased triglycerides and (2) compounds that would reverse childhood obesity are enriched for compounds indicated for coronary artery disease^41^, respectively (considering only chemogenomic enrichments with FDR < 0.25, Supplementary Data 6).

Taken together, these combined analyses illustrate the potential for the approach described in this study to inform drug discovery and drug development efforts. The identification of side effect, drug target and drug indication enrichments linked to known or plausible trait biology supports the veracity of the repurposing predictions deriving from the accurate prediction of known indications, and, more broadly, the power of integrative genomics approaches to identify molecular networks that underpin disease.

### Trait-trait correlations and gene-trait associations

**Fig. 5:**
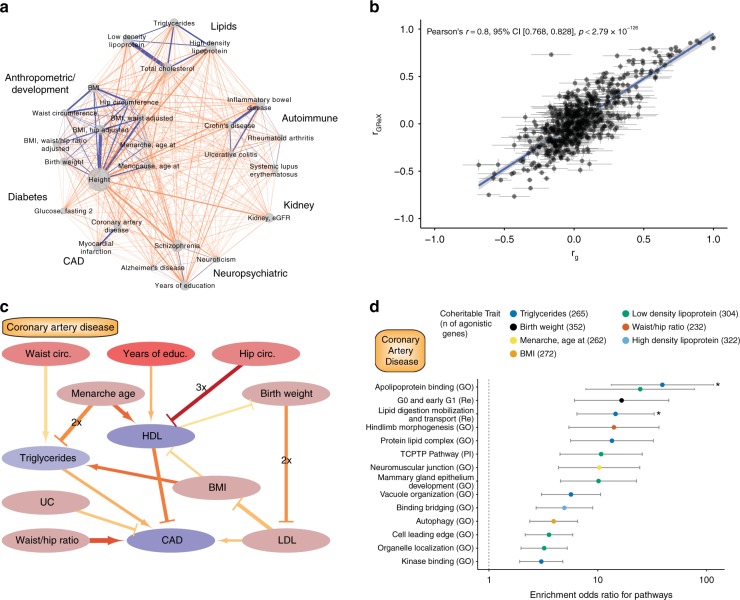
Trait-trait correlations and gene-trait associations. **a** Network indicating shared genes within/across trait categories. Only traits that have more than 50 associated genes are showcased. Edge width denotes number of shared genes for each trait pair. The node size indicates number of gene-trait associations for a given trait. Blue edges denote within-category trait associations and orange edges denote across-category trait associations. The analysis is based on significantly associated genes with FDR ≤0.5%. **b** Scatter plot of genetic correlation (*r*_g_) and genetically regulated gene expression (*r*_GReX_) for each pairwise trait combination. Standard error is shown with gray lines, *r*_g_ and r_GReX_ are highly correlated (Pearson’s *r* = 0.8, *p* value < 2.79 × 10^−126^). **c** Causal trait network of CAD. CAD and up to two traits upstream are plotted in this network graph to demonstrate causal (arrows) and protective (bar-headed lines) relationships as estimated by bi-directional regression analysis. The trait nodes are colored based on the parent causal trait network of all the traits of the study (Supplementary Fig. 21); nodes that have more children than parent nodes are a darker shade of red and blue, respectively. In edges, width denotes absolute beta, redder color denotes lower *p* value, and the 2× or 3× labels denote that the relationship is identified in 2 or 3 tissues, respectively. The analysis is based on genes with FDR ≤1%, and only the relationships with *p* value ≤0.05 are shown. **d** Graph depicting the odds ratio of pathway enrichment for CAD agonistic genes shared with traits involved in the causal network. Briefly, for causal traits, a list of genes (with unadjusted *p* value ≤0.05) that are predicted to change to the same direction (or the opposite direction for protective traits) is used for GSEA for common pathways. In this graph only the top 15 (based on *q* value) results are shown and are ranked based on odds ratio; an asterisk (*) indicates results that have *q* value ≤0.05. Error bars represent 95% CI for each enrichment

To further understand trait relatedness, we construct a network based on pairwise trait comparison of genetically regulated expression (including traits with more than 50 significant associations). By using a broad categorization of traits (Supplementary Data 3), we identify 245 pairs of shared gene associations across trait categories and 66 pairs within trait categories (Fig. 5a, Supplementary Data 7). Higher numbers of genes are shared between traits that belong to the same trait category than those that do not; the highest number of genes is shared between low density lipoprotein and total cholesterol in the lipids category. Previous studies have shown significant genetic correlation among common traits^42,43^. Pairwise trait GReX correlation shows a positive association with genetic co-heritability^42,43^ (Pearson’s *r* = 0.8, *p* value < 2.79 × 10^−126^) (Fig. 5b), extending the genetic similarity among traits to specific genes.

We then apply bi-directional regression analyses^44^ on the GReX of different traits across all tissues to infer causal relationships among pairs of traits with significant genetic and GReX correlation (Fig. 5c for CAD and Supplementary Fig. 21 for all the traits in our study). We find evidence that CAD is a complex trait whose predicted gene expression changes can be partly, but directly, explained by predicted expression changes found in individuals with elevated triglycerides, elevated LDL, and increased waist/ hip ratio. On the other hand, predicted expression changes in individuals with increased HDL, or those suffering from ulcerative colitis (UC), are expected to normalize expression changes in individuals with CAD. By expanding the causal network to include more upstream traits, we can see that another 6 traits (waist and hip circumference, years of education, age at menarche, birth weight, and BMI), which are correlated, or anti-correlated, with CAD may cause, or protect, from the predicted expression changes through effects on intermediate traits (Fig. 5c). For example, waist circumference acts via a causal relationship with triglycerides; other traits follow multiple pathways such as age at menarche, which opposes predicted transcriptomic changes of the increased triglycerides group while promoting imputed transcriptomic changes for individuals with high HDL. We then leverage these causal networks to dissect the pathogenesis of CAD by identifying the molecular pathways shared among all the involved trait pairs. For each trait that can cause or protect from CAD, we identify the agonistic genes—genes whose predicted expression is changing towards the same or opposite direction for causal (e.g., triglycerides) and protective (e.g., HDL) traits, respectively. Gene set enrichment analysis of agonistic genes for biological pathways point towards biologically relevant processes for CAD (Fig. 5d). For example, a subset of CAD genes (*n* = 256 out of 2806 genes with *p* value ≤ 0.05) is shared with triglycerides and affects biological processes related to apolipoprotein binding and lipid digestion, mobilization, and transport.

Taken together, the pairwise GReX trait correlations illustrate the potential to identify genes that are shared among genetically correlated traits. Agonistic versus antagonistic pleiotropy among two traits can be differentiated by leveraging the directionality of gene expression association in each trait. For traits, such as CAD, this analysis can be applied to dissect the complex phenotype, to identify genes and pathways that are shared with another trait, and potentially identify and develop therapeutic strategies to reverse those perturbations.

## Discussion

The maps of gene expression and regulatory annotations, generated by projects such as REMC^15^, CommonMind^18^, GTEx^20^, and STARNET^19^ hold the potential to further our understanding of non-coding risk genetic variation. Here we describe EpiXcan which, compared to PrediXcan, integrates biologically relevant data in a single framework to improve predictive performance of transcriptome imputation. EpiXcan is also better powered to identify clinically significant results such as enrichment for loss-of-function intolerant genes in neuropsychiatric traits^33,34^ and can detect more robust gene expression changes in genes associated with severe forms of the trait. Despite improvements in transcriptomic imputation predictive performance, it is important to note that all current imputation methods overall explain a small proportion of gene expression variation. We apply EpiXcan prediction models from 14 tissues in 58 common and complex traits and examine properties of those associations.

First, gene associations are predominantly identified in pathophysiologically relevant tissues and most associations are only identified in one tissue. Considering that the average correlation between genetically regulated gene expression of unrelated tissues such as blood and brain across 58 traits is 0.38–0.42 (Spearman’s *ρ*), we highlight the need for trait-relevant tissue datasets for such studies to be more effective.

Second, among genes associated with the traits in this study, we observe significant enrichment for biological pathways involved in trait pathophysiology. Moreover, gene-trait associations are significantly enriched for: (1) pathogenic (or likely pathogenic) genes for the given trait (clinVar), (2) genes associated with trait-relevant phenotypes (SoftPanel), (3) genes that have been associated with clinical signs relevant to the trait (OMIM CS), and (4) orthologous mouse genes with phenotypes that belong to the same phenotypic category as the given trait. This suggests that common variants partly act via smaller effect size perturbations in genes that lead to more severe forms of the phenotype when subject to larger effect size disruptions, as recently similarly suggested^27^.

Third, by leveraging trait-specific transcriptomic changes, we identify compounds that can reverse trait-specific changes, pointing to potential drug repurposing candidates. We assess the performance of our pipeline by comparing the predictions with known drug indications and find drugs that are predicted to normalize trait-specific changes are more likely than expected to be disease modifying for the trait. Towards further validation of our approach, chemogenomic enrichment analysis reveals trait-specific, phenotypically related, side effects, drug indications and drug target enrichment for risk-associated genes of phenotypically related traits. One recent study^14^ applied a similar approach, which was somehow limited in scope (brain tissue −10 regions—transcriptomic imputation with S-PrediXcan for psychiatric traits). It is hard to directly compare the results of the two studies since our approaches differ on many levels: we use EpiXcan, train models on more diverse tissues, employ a different drug repurposing pipeline that includes a set of chemogenomic enrichment analyses and use a more agnostic approach to validate our predictions. Despite the above limitations, both approaches share a lot of similarities, including their use of the same compound signature reference panel source and similar principles for ranking the compound predictions. Among common traits between the two studies, we do not find a particularly high concordance among our predictions (OR range: 0.91–1.31) but we do find that our predictions (1) are more likely to agree for schizophrenia (OR = 1.31, *p* value = 0.026) and (2) have higher concordance the higher the brain tissue enrichment score for the trait (*p* value < 2.2 × 10^−16^) compared to other tissues (Supplementary Note 1, Supplementary Fig. 22). For schizophrenia—where our results are most concordant—their studies identify no candidate compounds after adjustment for multiple testing. In contrast, EpiXcan identifies one statistically significant result (phenformin, Supplementary Data 6) that is a very potent antidiabetic agent (no longer FDA-approved due to safety concerns) which is not surprising given that glucose homeostasis is altered from illness onset in schizophrenia^45^. Within the top 10 results for schizophrenia, we also identify a potent antipsychotic (prochlorperazine), a voltage-gated sodium channel^46^ inhibitor (pramocaine) and guanfacine, which was trialed for cognitive impairment in schizophrenia and found to be worthy of further investigation^47^. Although our approach performs remarkably well given the modest percentage of gene expression variation that we are able to explain (despite performance improvements from EpiXcan), there are several limitations that are hampering its translational potential. Towards further improving common variant derived GReX-based CDR pipelines, generating cell-type specific predictive models with spatial and temporal annotation, as well as expanding and improving the repertoire of compound signatures in more relevant cells and in vivo models, holds much promise in establishing a powerful genetically driven drug discovery and repurposing pipeline.

Finally, we use bi-directional regression analysis^44^ to construct putative causal trait networks. Causal trait networks built on top of EpiXcan are sufficiently powered to provide valuable insight into the development of complex traits such as CAD. For example, we find that high BMI can influence CAD by two distinct pathways; (1) by positively influencing triglycerides (TG) which would positively influence CAD, and, conversely, (2) by negatively influencing HDL which would negatively influence CAD. The independent effect of BMI on TG and HDL has been shown in a population with a broad spectrum of BMI values^48^ which—as in our study—found no effect of BMI on LDL levels. Downstream, there is genetic evidence to suggest a causal influence of TG on CAD^49^. In addition, a negative correlation of HDL with CAD has been established in observational epidemiology, although a link between genetic loci causal for high levels of HDL and protective for CAD is, at present, elusive^50^. The construction of these causal trait networks allows us to identify genes that exhibit agonistic pleiotropy participating in shared pathways. Such information could potentially be used to develop distinct therapeutic strategies based on individual comorbidities.

Overall, the described method utilizes epigenomic information to further improve prediction of transcriptomes and it provides a framework for TWASs, improved interrogation of trait-associated biological pathway involvement, and a platform for drug repurposing and treatment development.

To facilitate interpretation, we provide the EpiXcan pipeline, trained models and resulting data tables as an online resource.

## Methods

### Genotype and expression data

Genotype datasets (CMC, GTEx and STARNET) are uniformly processed for quality control (QC) steps before imputation. We restrict our analysis to samples with European ancestry (Supplementary Methods). Genotypes are imputed using the University of Michigan server^51^ with the Haplotype Reference Consortium (HRC) reference panel^52^. RNAseq gene level counts are adjusted for known and hidden confounds, followed by quantile normalization. For CMC gene expression, we use the gene level counts generated from DLPFC RNAseq data^18^ (http://commonmind.org/). For GTEx^53^, we use publicly available, quality-controlled, gene expression datasets from the GTEx consortium (http://www.gtexportal.org/). RNAseq data for STARNET were obtained in the form of residualized gene counts from a previously published study^19^ [https://www.ncbi.nlm.nih.gov/projects/gap/cgi-bin/study.cgi?study_id=phs001203.v1.p1]. Additional information for CMC, STARNET and GTEx tissues (for both predictors and observed datasets) including sample sizes is shown in Supplementary Table 1. To compare the prediction accuracy of the CMC-trained predictors, we utilize expression data from the HBCC (*n* = 280 samples^18^), as well as 13 brain areas from GTEx^53^ (Supplementary Table 1). The GTEx data are publicly available de-identified data, whereas ethical approvals of STARNET and CMC data are detailed in the original papers.

### SNP priors and rescaling to WENet penalty factors

To leverage epigenomic information, we incorporate rescaled SNP priors as penalty factors into a weighted elastic net model. First, we compute eQTLs using MatrixEQTL^54^. Then, epigenome annotations from REMC^15^ are integrated to obtain SNP priors using qtlBHM^17^ (top panel in Supplementary Fig. 1**;** Supplementary Methods**;** Supplementary Data 8). Lastly, the SNP priors are rescaled to penalty factors used in WENet by a data-driven rescaling equation. The optimal rescaling equation is approximated by the best performing quadratic Bézier function, providing both the curve of the rescaling function and the minimum value of the penalty factors. Briefly, to determine the best performing rescaling equation, we simulate genotypes (*n* = 500 samples) using HAPGEN2^55^ and haplotypes from the 1000 Genomes Project^56^. For each gene under consideration, we utilize a shifting window policy to generate quadratic Bézier rescaling equations. In each separate window, we define a minimal penalty factor (Supplementary Fig. 23) and, within that window, evaluate possible intermediate Bézier curve control point locations to test for a wide range of curves for our rescaling equation (Supplementary Fig. 24). The equation that exhibits the highest improvement of *R*^*2*^_CV_ when compared to not assigning penalty factors to the SNPs (as in PrediXcan) is selected. The process to evaluate and select the optimal rescaling equation is described in greater detail in Supplementary Methods.

### Simulation analysis and predictive performance comparisons

Five hundred samples are simulated to verify the model performance. For specific gene, suppose **X** is the matrix containing genotypes of all *cis-*SNPs included in the gene. For the *i-*th SNP, we choose an effect estimate **β**_*i*_, so we have vector of estimated effects **β** for all the SNPs of the gene. Gene expression values are simulated by

1 $${\mathbf{y}} = {\mathbf{X}} \times {\mathbf{\beta }} + {\mathrm{level}} * {\mathbf{\varepsilon }}$$

Here ‘×’ denotes matrix-vector product and *ε* is normally distributed noise with given standard deviation (SD = 0.3). We select ten levels (Level from 0.1 to 1) of noise to simulate expression values for given genes. The CMC eQTL beta values are used as the effects in the simulation. We use 1000 genes with the highest significance from CMC eQTL studies to perform the simulations. For each gene, we simulate 50 times and take the mean value to evaluate the closeness between simulated and real-world gene expressions.

### Comparison with BSLMM and DPR methods

We perform a comparison, more limited in scope than in Fig. 1, of EpiXcan with PrediXcan, BSLMM and DPR. We use the CMC dataset for training and cross-validation (CV) and HBCC as an independent test dataset to calculate the gene expression imputation *R*^2^, as well as the per gene computation duration required by each method. For estimating the *R*^*2*^_CV_, we utilize four folds of the CMC samples for training and then the remaining one fold to test the prediction performance. Similar approaches for predictive performance comparisons were employed in previous studies^22^. To estimate the *R*^*2*^_PP_ in independent datasets, we use all CMC samples for training and the HBCC dataset to test the predictive performance. DPR has the option to use two different fitting algorithms: (1) the mean-field variational Bayesian (VB) approximation and (2) the Monte Carlo Markov Chain (MCMC). We perform the above tests and measure the predictive performance and imputation speed for EpiXcan, BSLMM, DPR (VB), DPR (MCMC), as well as PrediXcan. Details about different package implementation parameters are given in Supplementary Methods.

### Large scale gene-trait association analysis

We train predictors of gene expression by applying EpiXcan and PrediXcan to genotype and RNAseq datasets across 14 tissues (Supplementary Table 1). For each tissue, we keep genes with pred.perf *q* value of the correlation between cross-validated prediction and observed expression (pred.perf^12^) ≤0.01. We identify gene-trait associations by jointly analyzing summary statistics from 58 complex traits (Supplementary Data 3) and gene expression predictors using S-PrediXcan^27^. SNPs in the broad major histocompatibility complex (MHC) region (chromosome 6: 25–35 Mb) are removed. *p* values are adjusted using the Benjamini-Hochberg method of controlling the false discovery rate at ≤0.01. The gene-trait associations that remain after this filtering are considered significant. The analysis of the GWAS data pertains to de-identified summary-level data and requires no ethical approval.

Uniquely identified genes by EpiXcan (or PrediXcan) are genes that are identified in significant gene-trait associations with one method but not the other. For gene-trait associations found in multiple tissues, we categorize genes as upregulated (or downregulated) in the trait if there are more tissues in which the effects are towards the indicated direction. If there are equivalent numbers of tissues in which the gene is positively and negatively correlated with a given trait, we categorize the gene regulation as ambiguous. Transcriptomic imputation yields approximately the same number of genes predicted to be upregulated or downregulated (*z* scores) across each trait (Supplementary Fig. 25). To construct the shared gene network in Fig. 5a: (1) we filter genes so that those with pred.perf *q* values ≤0.5% and FDR-adjusted *p* values ≤0.5% are retained, (2) specifically for shared genes across traits of the same category, we only include genes with high effects (e.g. $\left| {z\;{\mathrm{score}}} \right| \ge {\mathrm{mean}}_i\left| {z\;{\mathrm{score}}} \right|_i,\;i$ is number of genes) to limit network density.

For identification of novel genes outside of GWAS loci, we define index SNPs based on LD clumped regions using Plink software (v1.9)^57^. The following settings are used: (a) significance threshold for index SNPs is 5 × 10^−8^, (b) significance threshold for clumped SNPs is 5 × 10^−8^, (c) clumping window size is 250 Kb and (d) LD threshold for clumping is 0.1. The coordinates of the GWAS loci are defined as 1 Mb on either side of the index SNP in each clump. The genomic coordinates of the significant genes are then extracted from GENCODE (build GRCh37, release 19) and overlapped with the coordinates of GWAS loci. Properties of those genes that lie outside the overlaps are explored. To identify the background distribution of *p* values of LD clumped regions, we used Plink, as above, but with no significance thresholds and a clumping window size of 500 kb. In addition, MAGMA gene analysis^24^ is performed for 55 GWAS phenotypes. Genes significantly associated with the phenotypes are identified after adjusting for multiple testing correction using Benjamini-Hochberg method. Significant genes identified using EpiXcan and PrediXcan are compared with the significant genes identified using MAGMA (FDR < 0.01) to indicate how many genes are inferred by EpiXcan or PrediXcan but not by MAGMA. The difference in the number of genes that lie outside the overlaps (when compared to GWAS or MAGMA) identified between the two methods is calculated by subtracting the number of genes identified by PrediXcan from the number of genes identified by EpiXcan. The statistical significance is tested with the null hypothesis such that the mean difference is zero using one sample sign test (H_0_: $\tilde X = 0$).

To indicate enrichment or depletion of the trait in a given tissue we use the Pearson standardized residuals as tissue-specificity enrichment score (${\mathrm{Standardized}}\;{\mathrm{residual}}_{ij} = \frac{{n_{ij} - \hat \mu _{ij}}}{{\sqrt {\hat \mu _{ij}\;(1 - p_{i + })(1 - p_{ + j})} }}$, where *i* is row, *j* is column, *n*_*ij*_ are observed values, $\hat \mu _{ij}$ are expected values, *p*_*i*__+_ is the observed ratio of total row count for *i* divided by all observations and *p*_+*j*_ is the observed ratio of total column count for *j* divided by all observations as described in Agresti^58^. To see whether there is a deviation from the null hypothesis of statistical independence (e.g. in Fig. 3b, the tissue-trait combination does not affect the number of significant GTAs), we perform Pearson’s *χ*^2^ test of independence. This method is applied for Fig. 3b and Supplementary Figs. 20a, 22b.

We identify significant GTAs from EpiXcan and PrediXcan as described above (predictive performance *q* value ≤ 0.01 and FDR ≤ 0.01) that are also identified in our SMR study (*p*_SMR ≤ 0.05)^5^. We then classify them into GTAs with either good co-localization properties (*p*_HET ≥ 0.05) or not (*p*_HET < 0.05, rejecting the null hypothesis that there is a single causal variant affecting both gene expression and trait variation, Supplementary Note 1).

### Gene set enrichment analyses and phenotypic datasets

To investigate whether the genes associated with a given trait exhibit enrichment for biological pathways, we use gene sets from MsigDB 5.1^59^ and filter out non-protein coding genes, as well as genes that do not have eQTL. For the enrichment analysis we only consider traits with >10 genes identified in significant gene-trait associations; this condition is met for 43 traits in our study. Statistical significance is evaluated with one-sided Fisher’s exact test and the adjusted *p* values are obtained by the Benjamini-Hochberg method. Similarly, for Fig. 2c, we perform gene set enrichment analysis for all decile bins of pLI from ExAC^28^ (all results can be found in Supplementary Data 4). The phenotypic datasets: ClinVar, OMIM CS, SoftPanel, and MGD are prepared as described in Supplementary Methods and contain genes that are associated with one or multiple traits. The approximation of known gene-phenotype associations from these datasets allows us to (1) compare the power of EpiXcan vs. PrediXcan in identifying known gene-trait associations (as in Fig. 2d) and (2) evaluate the extent to which common risk variants confer trait risk by affecting gene expression levels of genes associated with monogenic forms of the trait or genes associated with similar-to-the-trait phenotypes in humans and mice.

### Computational drug repurposing

Compound profiles are sourced from Connectivity map, and are based on gene expression microarray data collected from 6100 individual experiments^36^, each comparing compound-treated with vehicle-treated cell line based gene expression profiles. We download the ranked log_2_ fold change matrix available for the 6100 individual experiments, and merge them into a single representative signature for the 1309 unique small molecule compounds (Supplementary Data 9) according to the prototype-ranked list method^60^.

We iterate over each trait considered in this study, retaining trait/gene associations with an FDR < 0.1, and converting HGNC gene symbols to NCBI entrez gene identifiers. If a gene is linked with a trait via an association that was detected in multiple tissues, the associations are summarized as the mean *z* score. There are 58 traits with a minimum of 5 positively, and negatively, associated genes and each of these query signatures (QS) are used for the subsequent drug repurposing.

For each trait, and each unique compound, we calculate a connectivity score (CS) using an approach described in Lamb et al.^36^ The calculation of the CS proceeds as follows: a running sum enrichment score (ES) is calculated for the negative (ES_Neg_) and positive (ES_Pos_) components of the QS, separately, reflecting the distribution of the QS component within the ranked gene list of the compound under consideration. ES can assume a value between −1 and +1, where a negative ES indicates that genes within a QS component are relatively downregulated by a compound, and a positive ES indicates that genes within a QS component are relatively upregulated. The two ES are then combined into a single CS: $= \frac{{{\mathrm{ES}}_{{\mathrm{Pos}}} - {\mathrm{ES}}_{{\mathrm{Neg}}}}}{2}$. The resulting CS thus assumes a range of [−1, +1] and aims to summarize the overall transcriptional relationship between a compound and a QS. We estimate statistical significance of a given CS by generating an empirical CS distribution for a given QS against 1000 permutations of compound signatures. Permuted compound signatures are generated by randomizing the ranked log_2_ of gene expression fold change for a given compound, and used to derive two-tailed *p* values, which are adjusted by the Benjamini-Hochberg method of controlling the false discovery rate.

For each trait, connectivity scores are then used to sort the list of 1309 compounds and used as the basis for a chemogenomic enrichment analysis. For each compound in the drug signature library, we collect diverse chemogenomic annotations, such as drug target information, side effect, therapeutic class associations, and drug indications. Side-effect associations are downloaded from Offsides^61^ and SIDER^61^ and connected to compounds in Connectivity map via Stitch identifiers. Drug target associations include targets referenced in DrugBank^39^, and also an augmented set of associations, based on predictions generated using the Similarity Ensemble Approach^40^. Drug disease indications were derived from the Clue Drug Repurposing Hub (https://clue.io/repurposing) and used to annotate compounds within the scope of our analysis with available trait indications. This resulted in 139 distinct clinical indications with at least three associated compounds. For each of these features, we calculate a signed running sum enrichment score, which reflects whether that feature is over-represented at the extreme ends of the drug list that has been ordered according to trait. Statistical significance of enrichment scores is based on comparison to a large distribution of permuted null scores, generated by calculating scores from randomized chemogenomic sets that contain an equivalent number of compounds to the true set being evaluated. *p* values are adjusted using the Benjamini–Hochberg method of controlling the false discovery rate.

We compile disease and trait risk associations from multiple sources, including HGMD^62^, ClinVar^29^, dbGAP^63^, Genetic Associations Disease^64^, GWAS catalog^65^, GWASdb^66^, Human Phenotype Ontology^67^, HuGE^68^, and OMIM^69^. Many of these are accessed through Harmonizome^70^. We use a Fisher’s exact test to compare each set of trait-associated drug targets (that contain at least 3 targets), with each disease risk gene set. The analysis is performed against a background of 2802 genes, representing the unique set of human drug targets in the combined set of referenced and predicted targets associated with the 1309 compounds. Two-sided *p* values are adjusted using the Benjamini-Hochberg method of controlling the FDR.

### Trait co-heritability analysis

To calculate the genetically regulated gene expression correlation (*r*_GReX_), as shown in Fig. 3a, we keep the significant imputed gene expression change (*z* score) values with *q* value ≤0.01 and perform pairwise tissue Spearman correlation analysis of the complete cases of *z* scores. To cluster the tissues together for plotting, we use hierarchical agglomerative clustering analysis with Ward’s method.

For genetically regulated gene expression correlation (*r*_GReX_), pairwise genetic correlation (*r*_g_), as shown in Fig. 5b, among traits analyzed by GWAS is taken from previously published reports^42,43^. For trait comparisons that appear in both studies we use the more recent study^43^. We consider the genetic correlation between traits significant if *q* value ≤0.05. To calculate *r*_GReX_, we keep the imputed gene expression values with unadjusted *p* value ≤0.05 and perform pairwise trait Spearman’s correlation analysis with Holm’s adjustment for multiple comparisons. To estimate the correlation of *r*_g_ and *r*_GReX_ for the trait pairs in our study we perform Pearson’s correlation analysis with Holm’s adjustment for multiple comparisons.

We identify all the significantly correlated trait-pairs (*r*_g_ and *r*_GReX_, *q* value ≤ 0.05 as above) and perform bi-directional regression analyses^44^ to identify causal relationships among the traits of our study (Supplementary Fig. 21). Then, taking as an example the coronary artery disease (CAD), we graph all the putative causal and protective relationships up to 2 nodes upstream in Fig. 5c (when the causal relationship is bi-directional between 2 traits, the relationship with the higher degrees of freedom is kept) and perform pathway enrichment analysis of shared agonistic genes for this causal network in Fig. 5d. For each causal or protective trait in the network, we generate a list of genes whose expression changes are predicted towards the same direction (or the opposite direction for protective traits) in CAD. These lists of shared agonistic genes are used for GSEA for common pathways. In Fig. 5d. only the top 15 (based on *q* value) results are shown and are ranked based on odds ratio.

### URLs

For CMC, see http://commonmind.org/; for Synapse for CMC data, see https://www.synapse.org/cmc; for GTEx portal, see http://www.gtexportal.org/; for MSigDB, see http://software.broadinstitute.org/gsea/msigdb; for EpiXcan website and repository, see http://icahn.mssm.edu/EpiXcan; for EpiXcan source code, see https://bitbucket.org/roussoslab/epixcan; for qtlBHM package, see https://github.com/rajanil/qtlBHM; for RHOGE package, see https://github.com/bogdanlab/RHOGE; for PrediXcan pipeline, see https://github.com/hakyim/PrediXcan; for PredictDB resource, see https://github.com/hakyimlab/PredictDB_Pipeline_GTEx_v7; for Clue Drug Repurposing Hub, see https://clue.io/repurposing; for DPR, see https://github.com/biostatpzeng/DPR; for BSLMM, see https://github.com/genetics-statistics/GEMMA/releases.

### Reporting summary

Further information on research design is available in the Nature Research Reporting Summary linked to this article.

## Supplementary information

Reporting Summary

Supplementary Information

Supplementary Data 1

Supplementary Data 2

Supplementary Data 3

Supplementary Data 4

Supplementary Data 5

Supplementary Data 6

Supplementary Data 7

Supplementary Data 8

Supplementary Data 9

Supplementary Data 10

Description of Additional Supplementary Files

Peer Review File

## Data Availability

The data sets analyzed during the current study are available for download from the links provided in the URLs section; of note is that some are controlled-access data. The data sets generated by the analyses of this study are provided as Supplementary Data files. Intermediate data sets derived from online aggregator databases will be made available from the corresponding author upon reasonable request.
