# Supplementary material for: Integrative transcriptome imputation reveals tissue-specific and shared biological mechanisms mediating susceptibility to complex traits
Source: Nat Commun. 2019 Aug 23;10:3834. doi: 10.1038/s41467-019-11874-7 (PMC6707297; doi:10.1038/s41467-019-11874-7)
Supplement: Supplementary file 1 — Reporting Summary [file 41467_2019_11874_MOESM1_ESM.pdf]

nature research

Corresponding author(s): Panos Roussos

Last updated by author(s): Jul 31, 2019

Reporting Summary

Nature Research wishes to improve the reproducibility of the work that we publish. This form provides structure for consistency and transparency in reporting. For further information on Nature Research policies, see Authors & Referees and the Editorial Policy Checklist.

Statistics

For all statistical analyses, confirm that the following items are present in the figure legend, table legend, main text, or Methods section.

n/a	Confirmed	
☐	☒	The exact sample size (n) for each experimental group/condition, given as a discrete number and unit of measurement
☐	☒	A statement on whether measurements were taken from distinct samples or whether the same sample was measured repeatedly
☐	☒	The statistical test(s) used AND whether they are one- or two-sided<br>*Only common tests should be described solely by name; describe more complex techniques in the Methods section.*
☐	☒	A description of all covariates tested
☐	☒	A description of any assumptions or corrections, such as tests of normality and adjustment for multiple comparisons
☐	☒	A full description of the statistical parameters including central tendency (e.g. means) or other basic estimates (e.g. regression coefficient) AND variation (e.g. standard deviation) or associated estimates of uncertainty (e.g. confidence intervals)
☐	☒	For null hypothesis testing, the test statistic (e.g. F, t, r) with confidence intervals, effect sizes, degrees of freedom and P value noted<br>*Give P values as exact values whenever suitable.*
☐	☒	For Bayesian analysis, information on the choice of priors and Markov chain Monte Carlo settings
☐	☒	For hierarchical and complex designs, identification of the appropriate level for tests and full reporting of outcomes
☐	☒	Estimates of effect sizes (e.g. Cohen's d, Pearson's r), indicating how they were calculated

Our web collection on statistics for biologists contains articles on many of the points above.

Software and code

Policy information about availability of computer code

Data collection	The data are collected through the online portals and/or websites that described in the main text and supplementary material of the paper. Detailed resources of the main data collection:<br>CMC: http://commonmind.org/<br>Synapse for CMC data: https://www.synapse.org/cmc<br>GTEx portal: http://www.gtexportal.org/<br>MSigDB: http://software.broadinstitute.org/gsea/msigdb<br>REMC: https://egg2.wustl.edu/roadmap/web_portal/<br>clinVar: https://egg2.wustl.edu/roadmap/web_portal/<br>OMIM: https://www.omim.org/<br>MGD: http://www.informatics.jax.org/downloads/TrackHubs/mm10/<br>MGI phenotypic alleles: http://www.informatics.jax.org/downloads/reports/MGI_PhenotypicAllele.rpt<br>ExAC pLI: ftp://ftp.broadinstitute.org/pub/ExAC_release/release0.3.1/functional_gene_constraint/<br>fordist_cleaned_exac_r03_march16_z_pli_rec_null_data.txt<br>Clue Drug Repurposing Hub: https://clue.io/repurposing<br>FDALabel: https://nctr-crs.fda.gov/fdalabel/ui/search
Data analysis	Data analysis was performed using R and python language. The source code that we wrote is released at:<br>https://bitbucket.org/roussoslab/epixcan<br>Other packages used:<br>qtlBHM package: https://github.com/rajanil/qtlBHM<br>RHOGE package: https://github.com/bogdanlab/RHOGE<br>PrediXcan pipeline: https://github.com/hakyim/PrediXcan<br>PredictDB resource: https://github.com/hakyimlab/PredictDB_Pipeline_GTEx_v7<br>SoftPanel: http://www.isb.pku.edu.cn/softpanel/<br>BigBedToBed: http://hgdownload.cse.ucsc.edu/admin/exe/linux.x86_64/bigBedToBed

DPR: https://github.com/biostatpzeng/DPR
BSLMM: https://github.com/genetics-statistics/GEMMA/releases

For manuscripts utilizing custom algorithms or software that are central to the research but not yet described in published literature, software must be made available to editors/reviewers. We strongly encourage code deposition in a community repository (e.g. GitHub). See the Nature Research guidelines for submitting code & software for further information.

Data

Policy information about availability of data

All manuscripts must include a data availability statement. This statement should provide the following information, where applicable:

- Accession codes, unique identifiers, or web links for publicly available datasets
- A list of figures that have associated raw data
- A description of any restrictions on data availability

The final generated datasets are available at the website: http://icahn.mssm.edu/EpiXcan. Intermediate data generated during the analysis will be available upon requests to corresponding author.

Field-specific reporting

Please select the one below that is the best fit for your research. If you are not sure, read the appropriate sections before making your selection.

☒ Life sciences ☐ Behavioural & social sciences ☐ Ecological, evolutionary & environmental sciences

For a reference copy of the document with all sections, see nature.com/documents/nr-reporting-summary-flat.pdf

Life sciences study design

All studies must disclose on these points even when the disclosure is negative.

Sample size	Sample sizes are determined by the original data files and the accompanied description files downloaded from the portals.
Data exclusions	For the performed analyses, we exclude data (GTEx and CMC studies) that were not from EUR population. Since all the samples in STARNET are from EUR population, we do not exclude specific samples. In addition, SNPs and genes in the broad major histocompatibility complex (MHC) region (chromosome 6: 25~35 Mb) are not included in the downstream analysis. Exclusion criteria were pre-established.
Replication	Reproducibility of the EpiXcan transcriptome imputation method was verified in independent test datasets as described. There was no attempt to reproduce the experimental findings in validation experiments.
Randomization	Randomization is not applicable in our study, we obtained already published datasets as above.
Blinding	Investigators were not blinded to group allocations since the information was publicly available. Detailed information was known to readers.

Reporting for specific materials, systems and methods

We require information from authors about some types of materials, experimental systems and methods used in many studies. Here, indicate whether each material, system or method listed is relevant to your study. If you are not sure if a list item applies to your research, read the appropriate section before selecting a response.

Materials & experimental systems

n/a	Involved in the study	
☒	☐	Antibodies
☒	☐	Eukaryotic cell lines
☒	☐	Palaeontology
☒	☐	Animals and other organisms
☒	☐	Human research participants
☒	☐	Clinical data

Methods

n/a	Involved in the study	
☒	☐	ChIP-seq
☒	☐	Flow cytometry
☒	☐	MRI-based neuroimaging
