# Supplementary material for: Integrative transcriptome imputation reveals tissue-specific and shared biological mechanisms mediating susceptibility to complex traits
Source: Nat Commun. 2019 Aug 23;10:3834. doi: 10.1038/s41467-019-11874-7 (PMC6707297; doi:10.1038/s41467-019-11874-7)
Supplement: Supplementary file 2 — Supplementary Information [file 41467_2019_11874_MOESM2_ESM.pdf]

Supplementary Information for

Integrative transcriptome imputation reveals tissue-specific and shared biological mechanisms mediating susceptibility to complex traits

Wen Zhang, Georgios Voloudakis, Veera M. Rajagopal, Ben Readhead, Joel T. Dudley, Eric E. Schadt, Johan L.M. Björkegren, Yungil Kim, John F. Fullard, Gabriel E. Hoffman, Panos Roussos

Correspondence to: P.R. (panagiotis.roussos@mssm.edu)

This PDF file includes:

Supplementary Methods
Supplementary Notes
Supplementary Figures 1 to 29
Supplementary Tables 1 and 2
Supplementary References

Table of Contents

Supplementary Methods 3
Weighted elastic net (WENet) model utilized in EpiXcan 3
SNP priors 4
Data-driven equation that rescales SNP priors to penalty factors 4
Bézier curves with interpolation functions 6
Genotype preprocessing 7
Estimating adjusted R^2 7
Implementations and comparisons with BSLMM and DPR 7
GWAS statistics 8
Enrichment of clinically significant genes 8
Preparation of the datasets 9
Gene set enrichment analysis for pLI 11
Z-score differences for clinical datasets 11
Assessment of computational drug repurposing (CDR) pipeline performance 11
Supplementary Notes 13
EpiXcan has better performance than PrediXcan 13
GTA colocalization property comparisons for EpiXcan and PrediXcan 13
Comparison of drug repurposing predictions with So et al. 14
Theorem and proof 15
Supplementary Figures 16
Supplementary Tables 50
Supplementary References 56

Supplementary Methods

Weighted elastic net (WENet) model utilized in EpiXcan

The elastic net (ENet) linear regression model is implemented in PrediXcan[1]. Criterion can be written as Supplementary Equation 1:

$$\mathbb{C}_{\text{ENet}}(\boldsymbol{\theta},\lambda,\alpha)=\sum_{i=1}^{n}[\boldsymbol{y_i}-\boldsymbol{X_i}\boldsymbol{\theta}]^2+\lambda\alpha|\boldsymbol{\theta}|_1+\lambda(1-\alpha)|\boldsymbol{\theta}|_2\,, \tag{1}$$

where $\boldsymbol{X_i}$, $1\le i\le n$, is the i-th row-vector of matrix $\mathbf{X}$ containing genotypes with dosages from 0 to 2. n is the number of samples. In Supplementary Equation 1, all SNPs are equally treated. In EpiXcan, we use a weighted ENet (WENet) model that incorporates penalty factors from rescaled SNP priors, the criterion of which can be written as Supplementary Equation 2:

$$\mathbb{C}_{\text{WENet}}(\boldsymbol{\theta},\lambda,\alpha)=\sum_{i=1}^{n}[\boldsymbol{y_i}-\boldsymbol{X_i}\boldsymbol{\theta}]^2+\lambda\alpha|\boldsymbol{\theta}|_{\boldsymbol{w}}+\lambda(1-\alpha)\boldsymbol{\theta}^T\mathbf{W}\boldsymbol{\theta} \tag{2}$$

In Supplementary Equation 2, $\mathbf{W}$ is the weight matrix that stores the penalty factors for SNPs. $|\boldsymbol{\theta}|_{\boldsymbol{w}}=\sum_{j=1}^{m}w_j|\theta_j|$, with w_j corresponding to the penalty factor of the j-th SNP. m is the number of *cis*-SNPs.

In Supplementary Equation 1, n-by-m matrix $\mathbf{X}$ encloses genotype of *cis*-SNPs of the specific gene for all samples, i.e., there are n samples and m *cis*-SNPs. $|\boldsymbol{\theta}|_1$ is the L_1 norm of $\boldsymbol{\theta}$, which is the coefficient vector of SNPs. $\boldsymbol{y}$ contains expression values of the specific gene for all the samples. y_i is the i-th entry of the response vector $\boldsymbol{y}$, which includes the expression value of the gene for the i-th sample. For later presentations, we use $\mathbf{x_j}$, $1\le j\le m$, to denote the j-th column vector of $\mathbf{X}$. Following the definitions, we know $\mathbf{x_j}$ encloses the genotype of the j-th SNP with respect to all the samples. The α parameter is set to 0.5 and λ is estimated via cross-validation (CV).

In Supplementary Equation 2, $\mathbf{W}$ is a diagonal matrix and its entries are the penalty factors utilized. We see from Supplementary Equation 2 that if $\mathbf{W}=\mathbf{I}$, which is the identity matrix, the algorithm becomes the standard traditional elastic net module (Supplementary Equation 1). From this perspective, the WENet model is more general and it consists of classic ENet as a special case. Supplementary Equation 2 contains three terms: the negative log-likelihood function of linear regression; the L_1 normalized term, which penalizes the L_1 norm of $\boldsymbol{\theta}$; and the ridge penalty, which can be formulated as the inner product of $\boldsymbol{\theta}$ with respect to matrix $\mathbf{W}$, $<\boldsymbol{\theta},\boldsymbol{\theta}>_{\mathbf{W}}$. In cases where $\alpha=1$ and $\lambda\neq0$, the method is reduced to Lasso. If $\lambda=0$, the method is even more simplified as a standard regression model without penalties. If all the penalty factors are 1's, *i.e.*, matrix $\mathbf{W}$ is identity matrix, the model is reduced to standard ENet without penalty weights.

The model employed by the elastic net method in Gamazon *et al*[1] is based on the criterion (Supplementary Equation 1), where all SNPs have the same penalty factor, which is set to 1 by default. Grouping effects[2,3] of

WENet model are provided in Theorem 1.

SNP priors

We first prepare eQTL statistics (computed with MatrixEQTL[4]) and SNP annotations (extracted from REMC https://egg2.wustl.edu/roadmap/web_portal/). For each eQTL tissue, we use the matched REMC tissue to extract the corresponding annotations (**Supplementary Data 8**). We then provide them as input of qtlBHM[5] that utilizes a Bayesian hierarchical model to calculate priors, which is a measure of SNP causality. Priors are derived from chromHMM[6] and, for each tissue, SNPs in the same state are assigned the equivalent priors based on the chromHMM tracks in which they are located. The REMC tissues that match eQTL tissues and prior statistics for all tissues of this study, for each annotation category, are provided in **Supplementary Data 8**. SNP priors for a given dataset can be calculated using our pipeline at https://bitbucket.org/roussoslab/epixcan; for the SNP priors included in this study, we offer them in the predictor databases as a direct download at https://icahn.mssm.edu/EpiXcan.

Data-driven equation that rescales SNP priors to penalty factors

The higher the estimated SNP priors given by qtlBHM[5], the higher the likelihood that the SNP has an important effect on gene expression. On the other hand, higher penalty factors in the WENet model denote a smaller effect on gene expression. Thus, optimal equations must be found to properly rescale priors to penalty factors. For this study, we developed a method based on Bézier curves employing a shifting-window strategy to approximate the data-driven rescaling function. Theoretically, we can have a different rescaling equation for each gene but, for simplicity and computational resource efficiency, for this study we opt to use one rescaling equation for all models of a given tissue. The steps of this method using the CMC tissue dataset as a template are as follows:

1) We perform PrediXcan and obtain the target R^2_{CV} for all genes. We then select 8 genes that are representative for different levels of R^2_{CV} that can be found in the study. For CMC we select the following genes (and provide the target R^2_{CV} for each one in parentheses): DDX11 (0.7631), ADAM15 (0.3029), C1orf112 (0.1497), C1RL (0.5098), ERBB3 (0.0204), ECT2L (0.0131), SEPT1 (0.0053), ZNF346 (0.0050)
2) We simulate 500 genotypes using HAPGEN2[7]. We use haplotypes from the 1000 Genomes Project[8] and a fine-scale recombination map[7] to simulate genotypes. We further filter the genotypes to include SNPs with MAF of at least 5%. We then keep the SNP structure for the *cis* SNPs of the 8 genes selected.
3) For each of the genes from (1), we perform simulations to select best rescaling function. All rescaling is based on quadratic Bézier curves. A *n*-th order Bézier curve is defined by a set of points, P_0 through P_n,

which are control points. The first (P_0) and last (P_n) control points are usually called the starting and ending control points of the curve. All other points are intermediate control points that do not lie on the curve, however, they decide the shapes of the curve. A *n*-th order Bézier curve has *n*+1 control points, *n*-1 of which are intermediate control points - a brief introduction of Bézier curves is enclosed in the next section. We then:

a. Define region of prior-to-penalty factor mapping. As shown in **Supplementary Figure 23b**, to map priors to penalty factors, we first define an area with x ∈ [0, maximal SNP prior], corresponding to the SNP priors and y ∈ [0, 1], corresponding to penalty factors.

b. Shifting-window policy. We then divide the rectangle region of prior-to-penalty factor mapping into several sub-windows. In each of the sub-windows, we have ranges of both the penalty factors and the priors. As described above, penalty factors should decrease with increasing values of priors, so that important SNPs can have a larger effect on transcriptomic imputation. We set the upper bound of penalty factors at 1 (as in the ENet model employed by PrediXcan) to which the minimal value of priors will be mapped (**Supplementary Figure 23b**). Since we do not have a lower bound of the penalty factors to which the maximal priors will be mapped, we map the maximal prior to the lowest rescaled factor value (y_2) in each sub-window starting from 0 and going all the way to 1 with step size 0.1 (step size is arbitrarily set) (e.g. in **Supplementary Figure 17a**, y_2=0.5).

c. Define a set of possible rescaling equations in each sub-window. Above, we set P_0 (0, 1), the starting point of the curve, and P_2 (x_2, y_2), where x_2 is the maximal point of the priors and y_2 with a range [0, 1] that is fixed for each sub-window (**Supplementary Figure 23**). For each sub-window, we define a grid (we set grid size of 0.1) denoting all the possible positions for P_1 intermediate control points that we will evaluate. For each different intermediate control point (P_1), we get a quadratic Bézier rescaling equation. An example is illustrated in **Supplementary Figure 24** (only a limited number of candidate rescaling functions are shown, although there are hundreds of possibilities).

d. Perform simulations to assess performance for each rescaling equation. We apply all possible Bézier curves as rescaling candidates to compute the $R^2_{CV(simulation)}$ for 100 times of gene-specific simulations. For each simulation we use the 500 simulated genotypes from (2) and assess performance ($R^2_{CV(simulation)}$) against simulated gene expression. Simulated gene expression is calculated by equation (2). Instead, we choose PrediXcan predictors as the effect estimates, and *noise* is normally distributed with a given standard deviation (SD=0.3).

e. Select the best performing rescaling equation in each sub-window from (3b). The rescaling

function with the highest improvement of $R^2_{CV(simulation)}$ from (3d) is chosen as the optimal rescaling in the sub-window. We select for maximal improvement as determined by:

$$\Delta R^2_{CV} = mean(R^2_{CV(simulation)} \text{ of EpiXcan}) - mean(R^2_{CV(simulation)} \text{ of PrediXcan}) \quad (3)$$

In **Supplementary Figure 26**, we show the optimal rescaling equations for each sub-window for gene *DDX11*.

f. <u>Select the best sub-window-specific performing rescaling equation from (3e) for each gene (from (1)).</u> For every optimal rescaling equation from each sub-window (e.g. **Supplementary Figure 26**), we perform another 100 simulations as described in (3d). We select the best performing one based on Supplementary Equation 3, which is the optimal rescaling equation for the gene.

g. <u>Select the best gene-specific performing rescaling equation to use for the tissue</u>. For each of the 8 genes from (1), we can see the best performing rescaling equations (**Supplementary Figure 27**). We perform EpiXcan using each of these rescaling equations and select the one that performs best based on R^2_{CV} improvement.

Finally, to conserve computational resources, we skew the rescaling equation from CMC, to fit the maximal priors (**Supplementary Data 8**) for all other tissues (**Supplementary Figure 28**). Here, we provide a framework for data-driven adaptive rescaling. Depending on the needs of each study, researchers may opt to estimate from scratch tissue-specific rescaling equations, or even use gene-specific rescaling equations.

Bézier curves with interpolation functions

The n-th order Bézier curve is determined by n+1 control points, of which n-1 are intermediate control points. For briefness, we list quadratic Bézier curves here and the function is given as

$$y = (1-t)^2 y_0 + 2t(1-t)y_1 + t^2 y_2, \quad x = (1-t)^2 x_0 + 2t(1-t)x_1 + t^2 x_2 \quad (4)$$

As variable t varies from 0 to 1, y is a function of x with second order. t is an intermediate variable, which is used to define Bézier function. x_k and y_k, k=0,1,…,n are the coordinates of control points.

Note that in Supplementary Equation 4, x and y are denoted by separate functions with respect to variable t. We deduce a function of y with respect to x by eliminating t to calculate rescaled values, which are the penalty weights or factors that used in EpiXcan. Here x stores primitive priors, from which we obtain the penalty factors y.

As we stated earlier, more intermediate control points are necessary for higher order Bézier interpolations. Theoretically, the more control points we use and the higher order of the interpolations, the higher accuracy will be achieved. To balance accuracy and computation complexity, we use quadratic (second order/degree) Bézier interpolations to approximate the rescaling equations and apply them to the EpiXcan approach. Selecting quadratic Bézier interpolation also has the benefit of not having to control for counter-intuitive increase of

penalty factors with increase in priors that can happen in the middle of the curve with higher order equations.

Genotype preprocessing

We remove samples with call rate < 0.95, sex mismatch (genetic sex different from pedigree sex) and autosomal heterozygosity deviation (|Fhet|>0.2). We remove variants with call rate < 0.95, Hardy-Weinberg equilibrium (HWE) p value < 1.0×10^{-6}. We identify related individuals using identity by descent analysis (IBD). One of each pair of related individuals (piHAT > 0.2) is removed at random. Since the entire STARNET cohort and the majority of the CMC cohort include individuals of EUR ancestry, we use genotype and gene expression information only from this population to train the models. For this, we merge the samples with 1000 genome EUR subset and do principal component analysis (PCA) using ~25,000 pruned and thinned variants. We plot first and second principal components and define an ellipsoid based on 1000G EUR samples (**Supplementary Figure 29**). Those that lie 8 SD away from the center of this ellipsoid are considered as genetic outliers and removed (**Supplementary Figure 29**). For post imputation, we remove variants with INFO < 0.8, minor allele frequency (MAF) < 0.01, more than one alternative allele. We also remove ambiguous alleles (A/T, G/C), indels (insertions and deletions) and variants without RS identifier.

Estimating adjusted R^2

We compare the performance of EpiXcan and PrediXcan models using adjusted R^2 (for both R^2_{CV} and R^2_{PP}), which control for sample size of the training dataset (same for both methods) and for the number of predictors in the model (differs for each gene between methods). We group the training samples *a priori* prior to the cross validation and use the same groupings in both EpiXcan and PrediXcan. The adjusted R^2 is computed using formula (Supplementary Equation 5) where R^2 is either R^2_{CV} or R^2_{PP}, n_{sample} is the sample size and n_{SNP} is number of SNPs in the model. For correlation R^2_{PP} adjustments, we use the n_{sample} and n_{SNP} in the source models (predictors, **Supplementary Table 1**).

$$R^2_{\mathrm{adj}} = 1 - \frac{(1-R^2)(n_{\mathrm{sample}}-1)}{n_{\mathrm{sample}}-n_{\mathrm{SNP}}-1} \qquad (5)$$

We use Wilcoxon and one-sample sign tests for the statistical comparisons of adjusted R^2_{CV} between PrediXcan and EpiXcan models.

Implementations and comparisons with BSLMM and DPR

To compare the performance of EpiXcan, PrediXcan, BSLMM and DPR (VB and MCMC) methods, we utilize

the CMC data for training and cross-validation performance (R^2_{CV}) comparisons and leverage the HBCC data set as an independent testing dataset to compare the R^2_{PP} (**Supplementary Figure 8**). We run BSLMM with its default parameters (e.g. SNP filtering with 1% MAF) enclosed in the GEMMA package[9]. For the DPR method[10], we utilize the DPR package provided by the authors. We use DPR_VB and DPR_MCMC to denote the fitting algorithm used in the DPR method, which correspond to the mean filed variational Bayesian (VB) approximation algorithm and the Monte Carlo Markov Chain (MCMC). We utilize the default settings for both VB and MCMC, i.e. '-dpr 1' for VB and '-dpr 2 -w 10000 -s 10000' for MCMC. We also collect information about per gene computation duration for each method (training and testing) and report the averages in **Supplementary Figure 8**. For BSLMM, the CPU time for relatedness matrix calculation is excluded.
For cross-validation, we use 80% of the CMC samples for training and the other 20% to calculate the R^2_{CV} for all genes amongst different methods. For independent dataset predictive performance, we train the tissue model in the whole CMC cohort and predict gene expression in the HBCC dataset. We then compute the correlation between predictive and observed HBCC gene expression (R^2_{PP}).

GWAS statistics

We download 58 GWAS summary statistics from public datasets and categorized traits into broad overall categories (**Supplementary Data 3**). Where multiple versions available, we use only the most updated version with the largest sample size. For some data sets, such as systemic lupus erythematous, we requested access to the GWAS data from authors. GWAS summary statistics for Alzheimer's disease are obtained from the International Genomics of Alzheimer's Project (IGAP), which is a two-stage study based on GWASs of European ancestry. IGAP uses genotyped and imputed data on ~7 million SNPs in stage I to analyze published GWAS datasets consisting of more than 17 thousand Alzheimer's disease subjects and 37,154 controls. For detailed information regarding resources of all the GWASs, please refer to **Supplementary Data 3**.

Enrichment of clinically significant genes

To compare the clinical significance of the gene-trait associations identified by EpiXcan and PrediXcan, we compile sets of known gene-trait associations by utilizing five different archives:

1. ClinVar[11]: a public archive of relationships among human sequence variation and phenotypes. We only keep the subset of entries that: a) have at least one current submission interpreting as pathogenic or likely pathogenic,

b) provide a gene name, c) provide a phenotype. This dataset allows trait-specific gene associations that have high confidence but returns a limited number of genes for each trait.

2. OMIM CS (OMIM Clinical Synopses): a custom subset of the OMIM[12] compendium. This subset is constructed by keeping the genes from the clinVar dataset, above, that have a corresponding OMIM ID associated with the entry. Then, by using the OMIM API, the Clinical Synopsis Data are fetched for each OMIM ID, allowing us to query trait-specific association of relevant clinical signs. Genes are thus linked with clinical signs from a big subset of genetic disorders, allowing for a greater number of gene-trait associations when compared with ClinVar.

3. SoftPanel[13]: a method for grouping diseases and related disorders for generation of customized diagnostic gene panels. For traits that have a corresponding ICD-10 number, we use the respective disorder or disorder group and extract the relevant gene sets. For traits that the latter extraction method does not yield any genes (either due to no ICD-10 classification equivalent or due to lack of genes identified with that method), we use the keyword-based search of SoftPanel which queries the OMIM database for keyword-matching disorders. The underlying design of the tool allows for even "softer" associations of the genes with the trait, thus providing a larger trait-specific list of genes when compared with the clinVar dataset and OMIM CS.

4. MGD (MGI Phenotypes)[14]: this dataset contains gene-phenotype associations from mouse lines. From this, we can infer trait-specific gene associations for the respective human ortholog genes. Direct phenotype overlap with human traits is challenging, as, in most cases, the mouse phenotype is more descriptive and does not use names of human diseases, disorders or syndromes; therefore, phenotype categories are used to query this database.

5. pLI (by ExAC)[15]: this dataset provides probabilities of loss of function intolerance (pLI) for each gene; the higher the pLI the higher the likelihood that this gene performs an essential function. This dataset does not provide trait-specific information but serves as an unbiased dataset to rank the "indispensability" of the genes.

Preparation of the datasets

ClinVar (dataset 1). The goal is to prepare a table that lists genes in clinVar that are likely pathogenic or pathogenic and associate them with traits; the following process was performed in January 2018: (1) the clinVar variant summary tabular file was downloaded from NCBI, (ftp://ftp.ncbi.nlm.nih.gov/pub/clinvar/tab_delimited/

variant_summary. txt. gz), (2) arrays of genes are excluded, (3) variants without gene names are excluded, (4) variants without associated phenotypes are excluded, (5) variants that don't have at least one current submission interpreting it as likely pathogenic or pathogenic are excluded, (6) the table is aggregated at the gene and phenotype level. The column "PhenotypeList" provides the phenotypes that are used for association with traits of our study, queries are performed as described in **Supplementary Data 10**. 135 unique genes from EpiXcan predictions are directly associated with our traits based on the query table.

OMIM CS (**dataset 2**). Briefly, the clinVar dataset (**dataset 1**) is used as a scaffold and is populated with information of clinical synopses from OMIM, as follows: (1) only the genes that have an associated OMIM ID were kept, (2) we obtain an OMIM API key and perform API calls to receive clinical synopsis information for each OMIM ID, while respecting call limitations to reduce server load (https://omim.org/help.api) by enforcing a sensible in-between calls time delay, (3) the acquired data are used to populate the table with clinical signs information, (4) only genes that have OMIM_CS (clinical synopsis) information are queried. Depending on the trait, specific keywords are used to search within the clinical synopsis data (**Supplementary Data 10**) and the identified genes are associated with the trait. 542 unique genes from EpiXcan predictions are associated with our traits.

SoftPanel (dataset 3). SoftPanel[13] is an online tool that generates panels of relevant genes based on several query types such as ICD-10 codes and keyword searches (also utilizing the OMIM API) for diseases and phenotypes. The tool can be accessed at http://www.isb.pku.edu.cn/softpanel/. The search terms for the 58 traits in our study are listed in **Supplementary Data 10**. 1,362 unique genes from EpiXcan predictions are associated with our traits.

MGD (MGI Phenotypes, **dataset 4**, accessed in June 2018**)**. The MGI phenotypes dataset can be generated as follows: (1) retrieve the .bb (big bed) files from http://www.informatics.jax.org/downloads/TrackHubs/mm10/ that have phenotype information, (2) convert .bb files to .bed files using the BigBedTo Bed binary (http://hgdownload.cse.ucsc.edu/admin/exe/linux.x86_64/bigBedToBed), (3) use the MGI_IDs from the bed file to query the list of all mouse phenotypic alleles (http://www.informatics.jax.org/downloads/reports/MGI_PhenotypicAllele.rpt) to get the respective MGI Marker Accession IDs, (4) use the MGI Marker Accession IDs to retrieve the (human) ENSEMBL IDs for each gene from a conversion table. (It can be

generated at https:// www. genenames. org/cgi-bin/download if the "Mouse Genome Database ID (supplied by MGI)" is included. 1,673 unique genes from EpiXcan predictions are associated with our traits.

pLI (Probability of loss-of-function intolerance**, dataset 5).** The generation of this dataset is previously described[15] and the table can be downloaded from (ftp://ftp.broadinstitute.org/pub/ExAC_release/release0.3.1/functional_gene_constraint/fordist_cleaned_exac_r03_march16_z_pli_rec_null_data.txt). Data are binned at 10% increments or thresholds as described. By convention we refer to genes belonging to the highest decile as extreme loss-of-function intolerant.

Gene set enrichment analysis for pLI

GSEA is performed for all pLI (probability of loss-of-function intolerant, **dataset 5**) deciles, p values are calculated with the fisher exact test and are FDR-adjusted to q values. We first perform GSEA for all significant genes and then perform a second separate GSEA for significant genes distributed in 8 lists, one for each trait category. No other pLI decile bins yield statistically significant results (q value < 0.05) as shown in **Supplementary Data 4**.

Z-score differences for clinical datasets

The $\Delta[z]$ (EpiXcan – PrediXcan) values for all the gene-trait associations that are significant from either EpiXcan or PrediXcan are considered. Each of the 5 panels corresponds to a different clinically relevant dataset (**datasets 1-5**). Of note is that the high pLI subset corresponds to pLI ≥ 0.9 (extreme loss of function intolerant genes) and is, by design, non-trait-specific (thus the higher number of observations in **Figure 2d**). p value is calculated with the one sample sign test against a theoretical median of 0 (H_0: $\tilde{X} = 0$). The ratio is the number of $\Delta[z]$ measurements in favor of EpiXcan to the respective number for PrediXcan.

Assessment of computational drug repurposing (CDR) pipeline performance

To objectively assess the computational drug repurposing pipeline performance, we compare the predictions against sets of real-world indications from two different sources:

1. PharmacotherapyDB 1.0: Physician-curated drug indications.

2. FDA-approved indications: Sourced with FDALabel (version 2.3) and manually physician-curated.

For the CDR compound predictions, we exclusively consider predictions that have a nominal p value < 0.3, which is stringent enough to have a certain level of confidence in the predictions.

Pharmacotherapy DB 1.0: A publicly available dataset[16] that sourced drug-disease pairs from MEDI-HPS[17], LabeledIn[18,19], EHRLink[20], PREDICT[21] and then assigned by physician curation to three different categories: a) disease modifying: "a drug that therapeutically changes the underlying or downstream biology of the disease", b) symptomatic: "a drug that treats a significant symptom of the disease" and c) non-indication: "a drug that neither therapeutically changes the underlying or downstream biology, nor treats a significant symptom of the disease".

FDALabel (version 2.3): We use FDALabel (https://nctr-crs.fda.gov/fdalabel/ui/search, keyword search terms and permanent links to search parameters are provided in **Supplementary Data 10**). The sourced trait-compound combinations included in our results (e.g. **Supplementary Table 2**) were manually curated by a physician and the following trait-compound-indication combinations were discarded:

- For obesity, isoniazid is not an FDA indication.
- For coronary artery disease, iodixanol is used in CCTA diagnostically (radiographic contrast agent) and is not an FDA indication.
- For Crohn's disease, cyanocobalamin is used to treat malabsorption caused by Crohn's but is not an FDA indication for the disease.
- For type 2 diabetes mellitus: a) fenofibrate is mentioned because it was not shown to reduce coronary artery disease morbidity and mortality in patients with type 2 DM, b) mifepristone is not to be used for the treatment of type 2 diabetes mellitus unrelated to endogenous Cushing's syndrome
- For ulcerative colitis, lidocaine is mentioned as one of the active ingredients for an FDA-approved interarticular joint kit that can also be used for intramuscular injection of triamcinolone acetonide for ulcerative colitis.

Supplementary Note 1

EpiXcan has better performance than PrediXcan

(1) Performance evaluation in brain tissue. Dorsolateral pre-frontal cortex (DLPFC) gene expression and CommonMind Consortium (CMC) genotype data are utilized as one of the training sets for our approach. Human brain collection core (HBCC) and Genotype-Tissue Expression (GTEx) brain tissue transcriptome data are used only for verification as test datasets (**Supplementary Table 1**). *(2)* Performance evaluation in cardiometabolic tissues. The Stockholm-Tartu Atherosclerosis Reverse Network Engineering Task (STARNET) dataset for seven tissues and the GTEx dataset for six tissues (same tissues as STARNET, excluding mammary artery) serve as training and test datasets, respectively and vice versa (**Supplementary Table 1**).

First, we use cross-validation to evaluate prediction performance. The majority of the reference panel genes (>90%) are contained in the EpiXcan-trained predictor database and the overall R^2_{CV} is better than PrediXcan trained PredictDB's (**Figure 1, Supplementary Figures 3, 4**) with significant pair-wise Wilcoxon test *p* value regarding all the datasets that we utilized (**Supplementary Data 1**). We list the numbers of genes with $R^2_{CV} \geq$ 0.01 from both models. Using 0.01 as the $\mathrm{R}^2_{\mathrm{CV}}$ cut-off, we detect more genes with EpiXcan having good performance. In addition, EpiXcan has lower root-mean-square error (RMSE) values, further indicating increased performance (**Supplementary Data 1**).

Finally, we use independent test datasets to evaluate prediction performance and external model validity. We use the CMC dataset[22] to train the brain tissue model using both EpiXcan and PrediXcan. Afterwards, we first use the trained database of predictors (PredictDBs) to predict transcriptomes using HBCC genotype data[22]. We show that, when using EpiXcan, higher correlations between predicted and observed expression (in HBCC brain) are obtained (**Figure 1; Supplementary Figure 5-7**), with pairwise Wilcoxon test *p* value $< 9.0 \times 10^{-16}$ (**Supplementary Data 2**). We then use the CMC-trained PredictDB's to predict GTEx brain tissue expression and compare it with observed expression values from 13 different brain regions in that cohort. For all the brain regions, EpiXcan improves prediction performance (**Figure 1; Supplementary Figure 5, 6**). Similarly, for cardiometabolic tissues we use 7 trained STARNET models to predict corresponding 6 GTEx transcriptomes and vice versa and, overall, observe better predictive correlation for EpiXcan-trained models (**Figure 1; Supplementary Figure 5, 7**).

GTA colocalization property comparisons for EpiXcan and PrediXcan

To investigate whether the uniquely identified GTAs from EpiXcan still exhibit good co-localization properties as previously shown for PrediXcan[23], we limit our analysis to the GTAs identified in our previous SMR study

(p_SMR $\leq$ 0.05)[24]. We then classify them into GTAs with either good co-localization properties (p_HET $\geq$ 0.05) or not (p_HET < 0.05 rejecting the null hypothesis that there is a single causal variant affecting both gene expression and trait variation): For EpiXcan, we identify 189 GTAs that display good co-localization properties and 312 that do not. Similarly, for PrediXcan, we identify 135 GTAs that display good co-localization properties and 178 that do not. We find no significant difference in the ability of the two methods to uniquely identify GTAs with good co-localization properties (Pearson's χ^2 p value = 0.14).

Comparison of drug repurposing predictions with So et al.

So et al.[25] performed computational drug repurposing based on PrediXcan tissue gene expression prediction models from ten different GTEx brain regions to identify candidate therapeutic compounds. The five traits that are shared between our studies are: *(1)* AD = Alzheimer's Disease, *(2)* ADHD = Attention-Deficit/Hyperactivity Disorder, *(3)* ASD = Autism Spectrum Disorder, *(4)* Schizophrenia, and *(5)* Anxiety. The authors provided the top 100 compounds for each brain area predicted to normalize the gene-trait signature. For each trait, we combine all the predicted compounds from different brain regions in a single list containing all compounds that appear at least once. We then examine whether compounds that we predict to either normalize the gene-trait signature (Trait GReX antagonism) or induce a disease-like state (Trait GReX agonism) were included in this list or not (**Supplementary Figure 22a**). We notice that, out of all the common traits, only in schizophrenia are we more likely than not to find an agreement between our predictions and theirs (χ^2 p value = 0.026, OR = 1.31) and, looking back in **Figure 3b**, we see that the concordance of our predictions is higher when there are disproportionally more predictions originating from brain tissue (CMC). Since one of the main differences in our approach is that we leverage predictions from a more diverse set of tissues for our drug repurposing pipeline, we further explore this relationship as follows. By calculating enrichment scores (as in **Figure 3b**, described in **Online Methods**) for significant GTAs identified in brain tissue versus all other tissues pooled together (**Supplementary Figure 22b**), we show that the higher the brain tissue enrichment score, the higher the concordance between our predictions and theirs (Spearman's $\rho = 1$, p value < 2.2×10^{-16}, **Supplementary Figure 22c**). Of note is that we exclude the traits "anxiety, case/control" and "anxiety, factor scores" from the analysis because they yield no significant GTAs in brain tissue in our study. Thus, we conclude (as summarized in the **Discussion**) that there is concordance in our computational drug repurposing pipeline findings especially when brain tissue prediction models contribute disproportionally more GTAs, such as in schizophrenia, but, overall, there is a high level of dissimilarity in our predictions.

Theorem and proof

Optimal coefficients in Supplementary Equation 2 are estimated by Supplementary Equation 6:

$$\hat{\boldsymbol{\theta}} = \underset{\boldsymbol{\theta}}{\operatorname{argmin}}\, \mathbb{C}_{\text{WENet}}(\boldsymbol{\theta}, \lambda, \alpha) \tag{6}$$

Grouping effect of the WENet model is given in Theorem 1.

Theorem 1. Suppose $\hat{\boldsymbol{\theta}} = \underset{\boldsymbol{\theta}}{\operatorname{argmin}}\, \mathbb{C}_{\text{WENet}}(\boldsymbol{\theta}, \lambda, \alpha)$, given data $(\mathbf{y}, \mathbf{X})$ where $\mathbf{X}$ is standardized, and parameters (λ, α), if $\hat{\boldsymbol{\theta}}_i \hat{\boldsymbol{\theta}}_j > 0$, define $D_{\lambda,\alpha}(i,j) = \frac{1}{|\mathbf{y}|_1} |w_i \hat{\boldsymbol{\theta}}_i(\lambda,\alpha) - w_j \hat{\boldsymbol{\theta}}_j(\lambda,\alpha)|$, then $D_{\lambda,\alpha}(i,j) \le \frac{\sqrt{2(1-\sigma)}}{\lambda(1-\alpha)} + \frac{\alpha|w_i - w_j|}{2(1-\alpha)|\mathbf{y}|_1}$.

Here σ is sample correlation of $\mathbf{x}_i$ and $\mathbf{x}_j$.

Proof

Since $\hat{\boldsymbol{\theta}}_i(\lambda,\alpha)\hat{\boldsymbol{\theta}}_j(\lambda,\alpha) > 0$, $\operatorname{sign}(\hat{\boldsymbol{\theta}}_i) = \operatorname{sign}(\hat{\boldsymbol{\theta}}_j)$. Because $\hat{\boldsymbol{\theta}} = \underset{\boldsymbol{\theta}}{\operatorname{argmin}}\, \mathbb{C}(\boldsymbol{\theta}, \lambda, \alpha)$, $\hat{\boldsymbol{\theta}}$ satisfies $\left.\frac{\partial \mathbb{C}}{\partial \boldsymbol{\theta}_k}\right|_{\boldsymbol{\theta}=\hat{\boldsymbol{\theta}}} = \mathbf{0}$ if $\hat{\boldsymbol{\theta}}_k(\lambda,\alpha) \neq 0$. Thus

$$\mathbf{2}(\mathbf{y} - \mathbf{X}\hat{\boldsymbol{\theta}})^{\mathbf{T}}\, \mathbf{x}_{\boldsymbol{k}} + \lambda\alpha \operatorname{sign}(\hat{\boldsymbol{\theta}}_k) w_k + \mathbf{2}\lambda(1-\alpha)\hat{\boldsymbol{\theta}}^{\mathbf{T}}\, W_k = 0$$

Here W_k is the k-th column vector of matrix $\mathbf{W}$. Hence

$$\mathbf{2}\left(\mathbf{y} - \mathbf{X}\hat{\boldsymbol{\theta}}\right)^{\mathbf{T}} \mathbf{x}_{\boldsymbol{i}} + \lambda\alpha \operatorname{sign}\left(\hat{\boldsymbol{\theta}}_i\right) w_i + \mathbf{2}\lambda(1-\alpha)\hat{\boldsymbol{\theta}}^{\mathbf{T}}\, W_i = 0 \tag{7}$$

$$\mathbf{2}\left(\mathbf{y} - \mathbf{X}\hat{\boldsymbol{\theta}}\right)^{\mathbf{T}} \mathbf{x}_{\boldsymbol{j}} + \lambda\alpha \operatorname{sign}\left(\hat{\boldsymbol{\theta}}_j\right) w_j + \mathbf{2}\lambda(1-\alpha)\hat{\boldsymbol{\theta}}^{\mathbf{T}}\, W_j = 0 \tag{8}$$

Subtracting Supplementary Equation 8 from Supplementary Equation 7, we have

$$\mathbf{2}\left(\mathbf{y} - \mathbf{X}\hat{\boldsymbol{\theta}}\right)^{\mathbf{T}} (\mathbf{x}_{\boldsymbol{i}} - \mathbf{x}_{\boldsymbol{j}}) + \lambda\alpha \operatorname{sign}\left(\hat{\boldsymbol{\theta}}_i\right)(w_i - w_j) + \mathbf{2}\lambda(1-\alpha)\hat{\boldsymbol{\theta}}^{\mathbf{T}}\, (W_i - W_j) = 0 \tag{9}$$

According to property of matrix $\mathbf{W}$,

$$\hat{\boldsymbol{\theta}}^{\mathbf{T}}\, (W_i - W_j) = \boldsymbol{w_i}\hat{\boldsymbol{\theta}}_{\boldsymbol{i}} - \boldsymbol{w_j}\hat{\boldsymbol{\theta}}_{\boldsymbol{j}} \tag{10}$$

From Supplementary Equation 9, Supplementary Equation 10 and Cauchy-Schwartz inequality as well as property of L_1 norm, we get

$$\left|\boldsymbol{w_i}\hat{\boldsymbol{\theta}}_{\boldsymbol{i}} - \boldsymbol{w_j}\hat{\boldsymbol{\theta}}_{\boldsymbol{j}}\right| \le \frac{\mathbf{1}}{\lambda(1-\alpha)} \left|\mathbf{y} - \mathbf{X}\hat{\boldsymbol{\theta}}\right|_{\mathbf{1}} \left|\mathbf{x}_{\boldsymbol{i}} - \mathbf{x}_{\boldsymbol{j}}\right|_{\mathbf{1}} + \frac{\alpha|w_i - w_j|}{\mathbf{2}(1-\alpha)} \tag{11}$$

From Zou et al.[26], we know

$$\frac{\mathbf{1}}{\lambda(1-\alpha)|y|_1} \left|\mathbf{y} - \mathbf{X}\hat{\boldsymbol{\theta}}\right|_{\mathbf{1}} \left|\mathbf{x}_{\boldsymbol{i}} - \mathbf{x}_{\boldsymbol{j}}\right|_{\mathbf{1}} \le \frac{\sqrt{2(1-\sigma)}}{\lambda(1-\alpha)} \tag{12}$$

Both sides of Supplementary Equation 11 being divided by $|y|_1$ and from Supplementary Equation 12 we get

$$\frac{1}{|\mathbf{y}|_1} |w_i \hat{\boldsymbol{\theta}}_i(\lambda,\alpha) - w_j \hat{\boldsymbol{\theta}}_j(\lambda,\alpha)| \le \frac{\sqrt{2(1-\sigma)}}{\lambda(1-\alpha)} + \frac{\alpha|w_i - w_j|}{\mathbf{2}(1-\alpha)|\mathbf{y}|_1} \tag{13}$$

Supplementary Figures

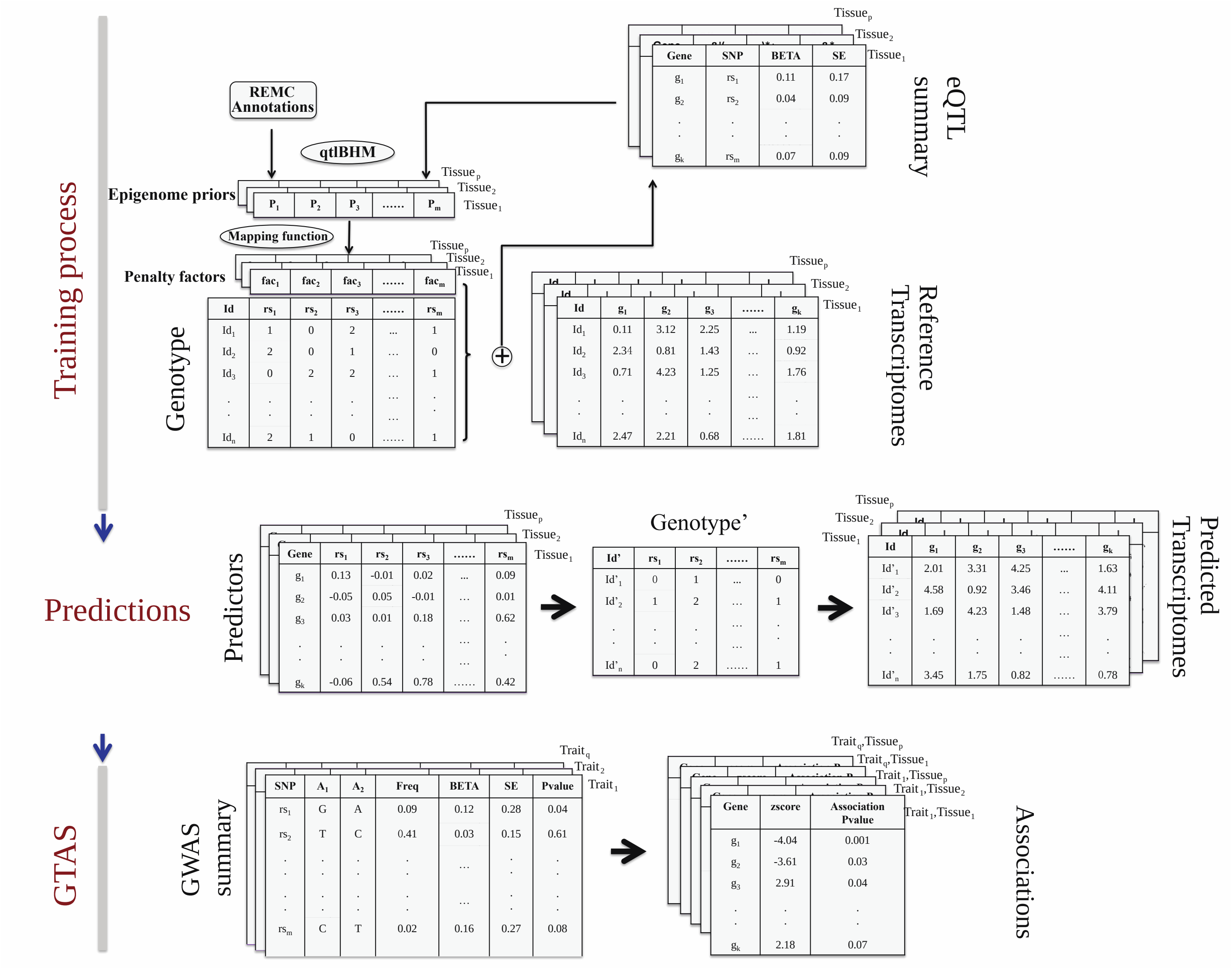

Supplementary Figure 1. Schematic of the EpiXcan workflow. For training of the prediction model (top panel), *m* genotypes and *k* transcripts are considered across *n* individuals in *p* tissue datasets. We obtain SNP priors by using a hierarchical Bayesian model (qtlBHM) that jointly analyzes REMC epigenome annotations and eQTL statistics. The priors are then transformed with an adaptive mapping function to penalty factors, which are then utilized by the WENet model. Using the WENet model, we jointly analyze SNP priors, genotypes and gene expression traits to estimate genetically regulated expression component across different tissues. For the gene-trait association studies (bottom panel), we integrate the SNP-transcriptome effect sizes with complex traits effect sizes to estimate the association between predicted gene expression and a trait, while taking in to consideration the linkage disequilibrium among SNPs.

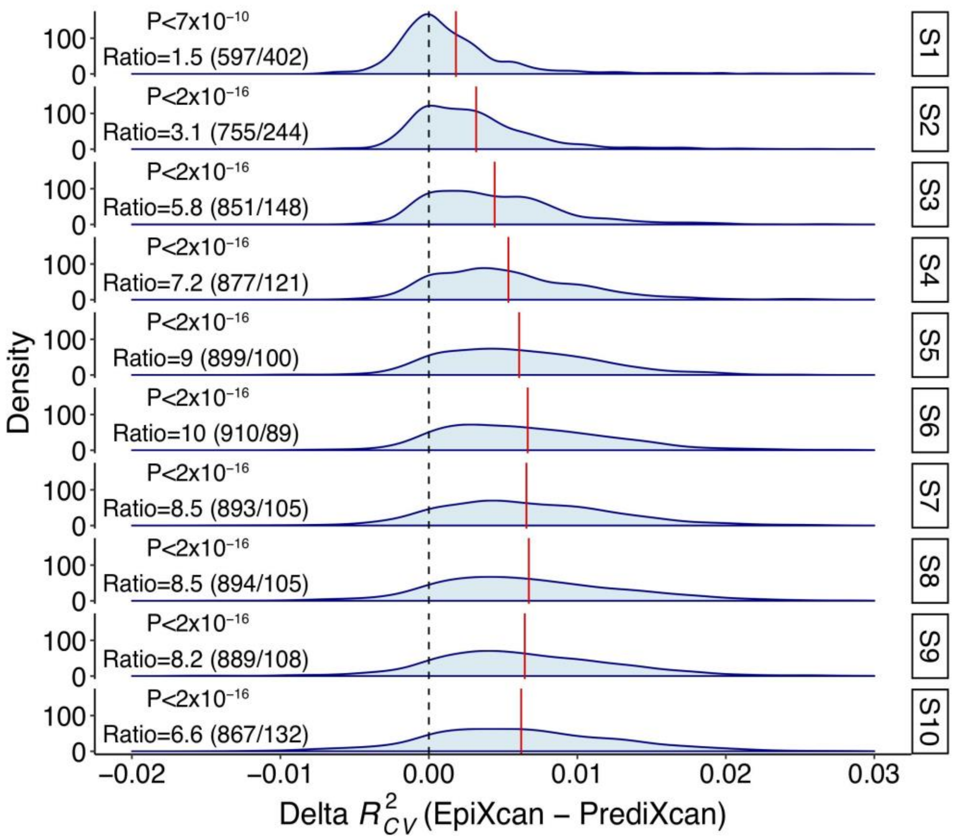

Supplementary Figure 2. Simulation results. Using simulated genotypes and gene expression in 500 samples, we compare the adjusted cross-validation (CV) R^2 of EpiXcan and PrediXcan by estimating the delta (Δ) R^2_{CV} value (EpiXcan R^2_{CV} minus PrediXcan R^2_{CV}). We simulated 10 scenarios, where in each scenario we increase the level of noise in the gene expression data. Across all simulations, the overall delta value is positive indicating that EpiXcan outperforms PrediXcan. *p* value from one-sample sign test is provided to compare whether the shift of the ΔR^2_{CV} values is different than zero (H_0: $\tilde{X} = 0$). The numbers in parentheses indicate the occasions where ΔR^2_{CV} was higher in EpiXcan ($\Delta R^2_{CV} > 0$; left number) and PrediXcan ($\Delta R^2_{CV} < 0$; right number); ratio is estimated by dividing the occasions of $\Delta R^2_{CV} > 0$ with $\Delta R^2_{CV} < 0$. The red vertical line shows the mean of delta value.

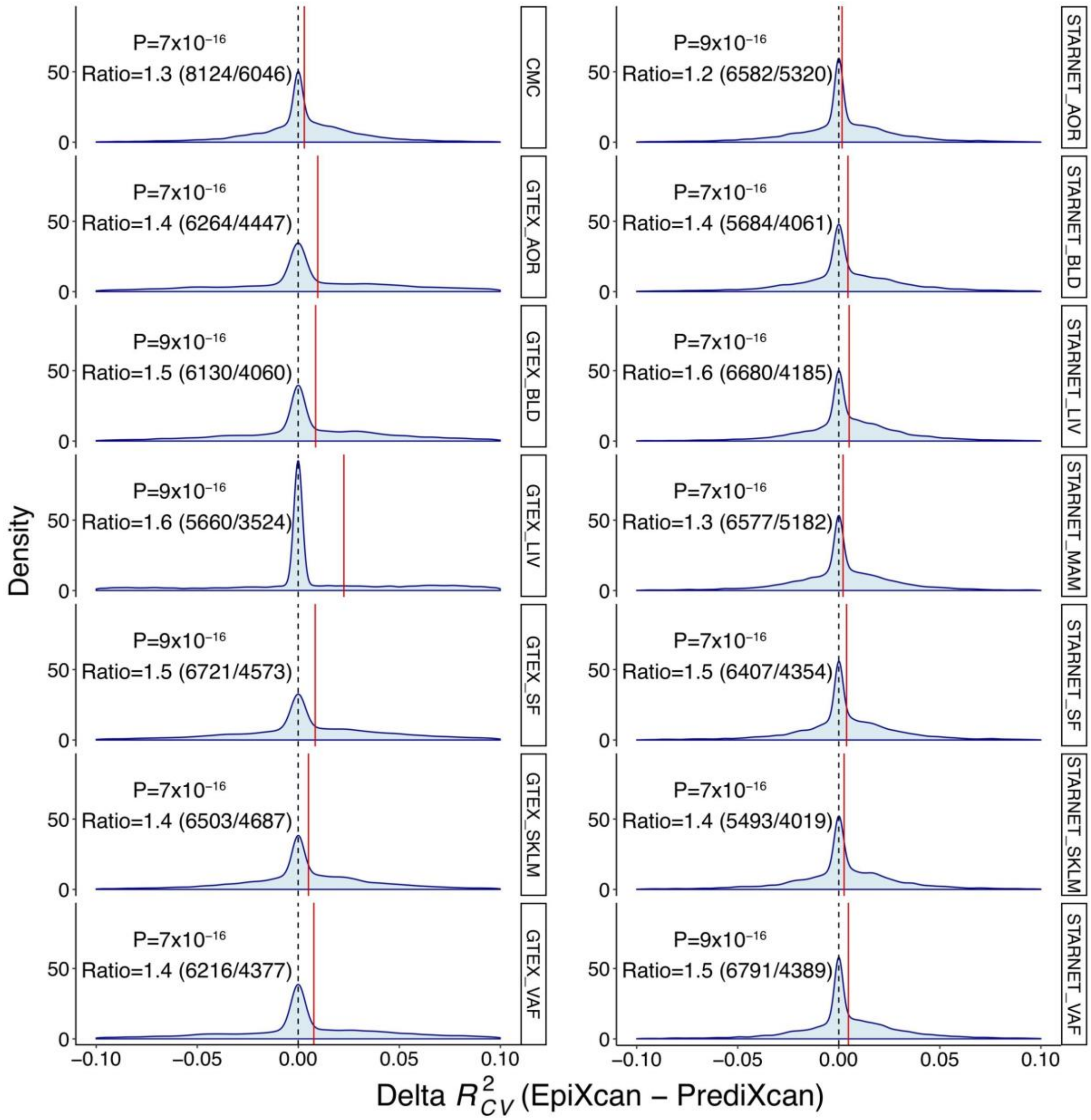

Supplementary Figure 3. Comparison of model performance during training (cross validation ΔR^2_{CV}). We apply EpiXcan and PrediXcan in 14 tissue datasets and compare the adjusted R^2_{CV} by estimating the ΔR^2_{CV} (EpiXcan R^2_{CV} minus PrediXcan R^2_{CV}). Across all datasets, the overall delta value is positive, indicating that EpiXcan outperforms PrediXcan. *p* value from one-sample sign test is provided to compare whether the shift of the ΔR^2_{CV} values is different than zero (H_0: $\tilde{X} = 0$). The numbers in parenthesis indicate the occasions where ΔR^2_{CV} was higher in EpiXcan ($\Delta R^2_{CV} > 0$; left number) and PrediXcan (delta $R^2_{CV} < 0$; right number); ratio is estimated by dividing the occasions of $\Delta R^2_{CV} > 0$ with $\Delta R^2_{CV} < 0$. The red vertical line shows the mean delta value.

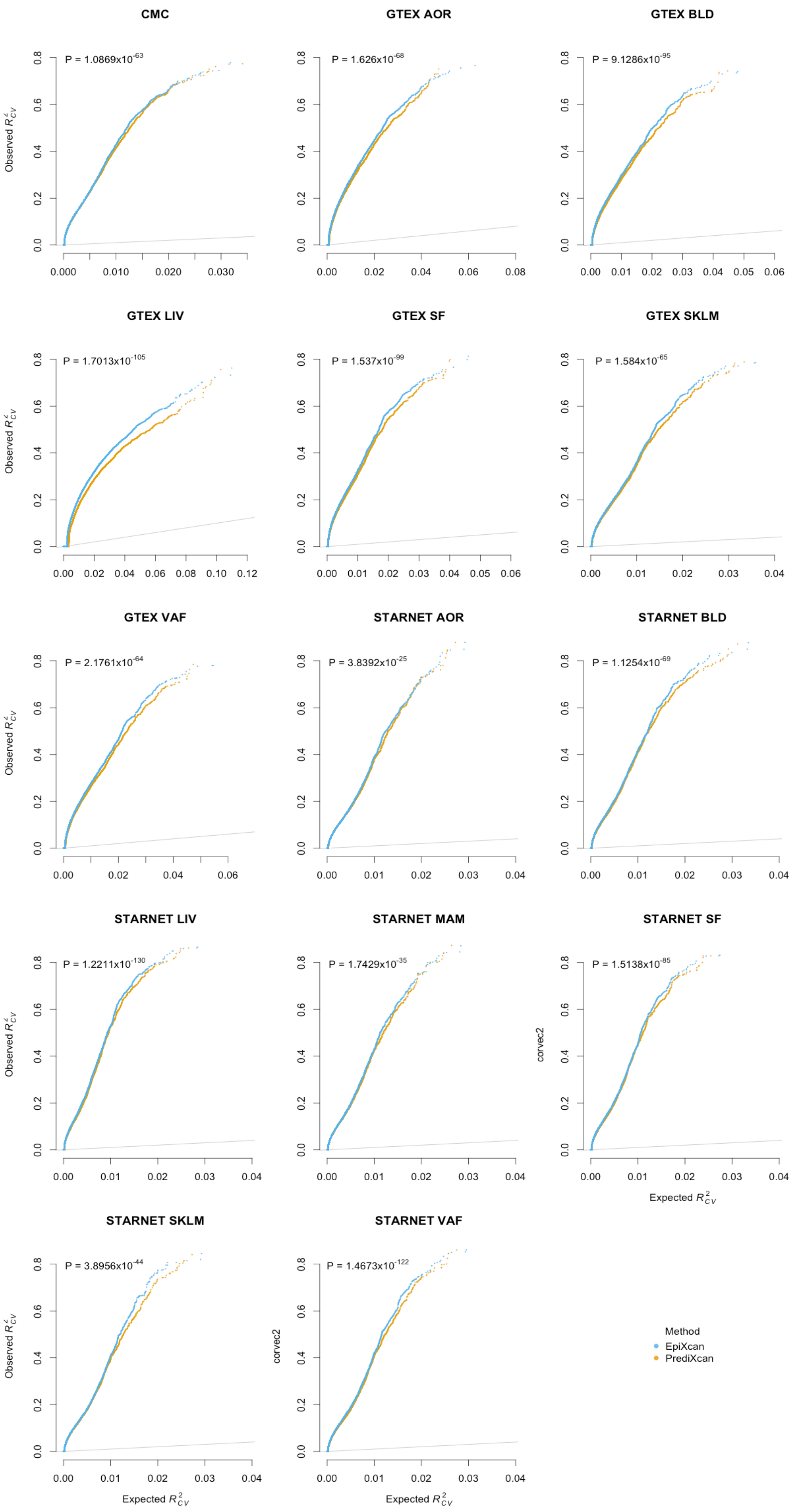
CMC
P = 1.0869x10^{-63}
GTEX AOR
P = 1.626x10^{-68}
GTEX BLD
P = 9.1286x10^{-95}
GTEX LIV
P = 1.7013x10^{-105}
GTEX SF
P = 1.537x10^{-99}
GTEX SKLM
P = 1.584x10^{-65}
GTEX VAF
P = 2.1761x10^{-64}
STARNET AOR
P = 3.8392x10^{-25}
STARNET BLD
P = 1.1254x10^{-69}
STARNET LIV
P = 1.2211x10^{-130}
STARNET MAM
P = 1.7429x10^{-35}
STARNET SF
P = 1.5138x10^{-85}
STARNET SKLM
P = 3.8956x10^{-44}
STARNET VAF
P = 1.4673x10^{-122}
Observed R^2_{CV}
corvec2
Expected R^2_{CV}
Method
EpiXcan
PrediXcan

Supplementary Figure 4. Comparison of model performance during training (cross validation R^2_{CV}). The adjusted R^2_{CV} of EpiXcan is higher than that of PrediXcan across all the tissues considered. The adjusted R^2_{CV} is employed to assess the prediction performance calculated from the cross validation R^2_{CV} after adjusting for the number of SNPs used by the prediction model for each gene. Expected R^2_{CV} corresponds to the null distribution. Statistical significance is evaluated using the pairwise Wilcoxon test.

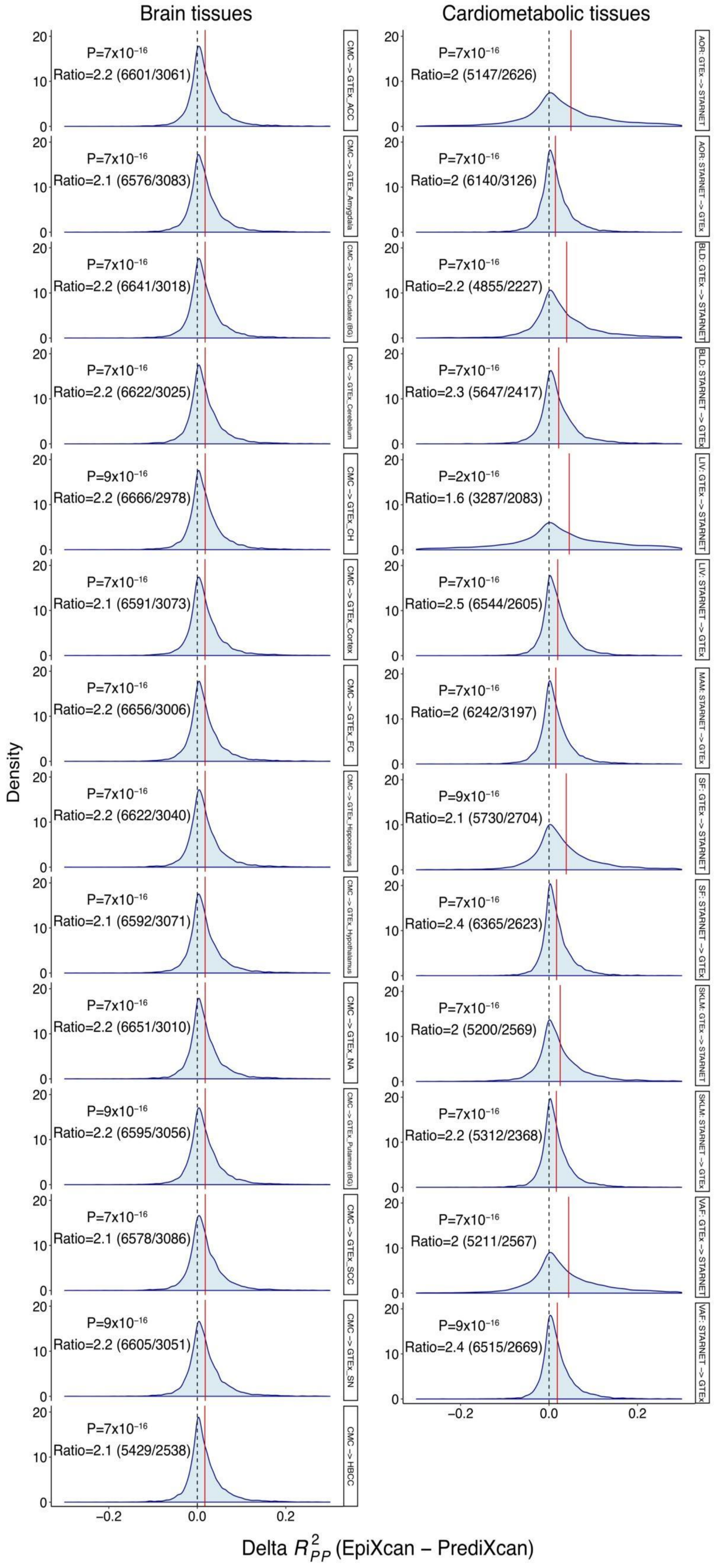

Brain tissues
Cardiometabolic tissues
P=7x10^-16
Ratio=2.2 (6601/3061)
CMC -> GTEx_ACC
P=7x10^-16
Ratio=2.1 (6576/3083)
CMC -> GTEx_Amygdala
P=7x10^-16
Ratio=2.2 (6641/3018)
CMC -> GTEx_Caudate (BG)
P=7x10^-16
Ratio=2.2 (6622/3025)
CMC -> GTEx_Cerebellum
P=9x10^-16
Ratio=2.2 (6666/2978)
CMC -> GTEx_CH
P=7x10^-16
Ratio=2.1 (6591/3073)
CMC -> GTEx_Cortex
P=7x10^-16
Ratio=2.2 (6656/3006)
CMC -> GTEx_FC
P=7x10^-16
Ratio=2.2 (6622/3040)
CMC -> GTEx_Hippocampus
P=7x10^-16
Ratio=2.1 (6592/3071)
CMC -> GTEx_Hypothalamus
P=7x10^-16
Ratio=2.2 (6651/3010)
CMC -> GTEx_NA
P=9x10^-16
Ratio=2.2 (6595/3056)
CMC -> GTEx_Putamen (BG)
P=7x10^-16
Ratio=2.1 (6578/3086)
CMC -> GTEx_SCC
P=9x10^-16
Ratio=2.2 (6605/3051)
CMC -> GTEx_SN
P=7x10^-16
Ratio=2.1 (5429/2538)
CMC -> HBCC
P=7x10^-16
Ratio=2 (5147/2626)
AOR: GTEx -> STARNET
P=7x10^-16
Ratio=2 (6140/3126)
AOR: STARNET -> GTEx
P=7x10^-16
Ratio=2.2 (4855/2227)
BLD: GTEx -> STARNET
P=7x10^-16
Ratio=2.3 (5647/2417)
BLD: STARNET -> GTEx
P=2x10^-16
Ratio=1.6 (3287/2083)
LIV: GTEx -> STARNET
P=7x10^-16
Ratio=2.5 (6544/2605)
LIV: STARNET -> GTEx
P=7x10^-16
Ratio=2 (6242/3197)
MAM: STARNET -> GTEx
P=9x10^-16
Ratio=2.1 (5730/2704)
SF: GTEx -> STARNET
P=7x10^-16
Ratio=2.4 (6365/2623)
SF: STARNET -> GTEx
P=7x10^-16
Ratio=2 (5200/2569)
SKLM: GTEx -> STARNET
P=7x10^-16
Ratio=2.2 (5312/2368)
SKLM: STARNET -> GTEx
P=7x10^-16
Ratio=2 (5211/2567)
VAF: GTEx -> STARNET
P=9x10^-16
Ratio=2.4 (6515/2669)
VAF: STARNET -> GTEx
Density
−0.2
0.0
0.2
Delta R^2_{PP} (EpiXcan − PrediXcan)

Supplementary Figure 5. Comparison of model performance during prediction in independent datasets (ΔR^2_{PP}). We used EpiXcan and PrediXcan models to predict expression levels in relevant brain and cardiometabolic independent datasets. We compare the adjusted predictive performance (R^2_{PP}) by estimating the ΔR^2_{PP} (EpiXcan R^2_{PP} minus PrediXcan R^2_{PP}). Across all datasets, the overall delta value is positive indicating that EpiXcan outperforms PrediXcan. p value from one-sample sign test is provided to compare whether the shift of the ΔR^2_{PP} values is different than zero (H_0: $\tilde{X} = 0$). The numbers in parentheses indicate the occasions where ΔR^2_{PP} is higher in EpiXcan ($\Delta R^2_{PP} > 0$; left number) and PrediXcan ($\Delta R^2_{PP} < 0$; right number); ratio is estimated by dividing the occasions of $\Delta R^2_{PP} > 0$ with $\Delta R^2_{PP} < 0$. The red vertical line shows the mean of delta value.

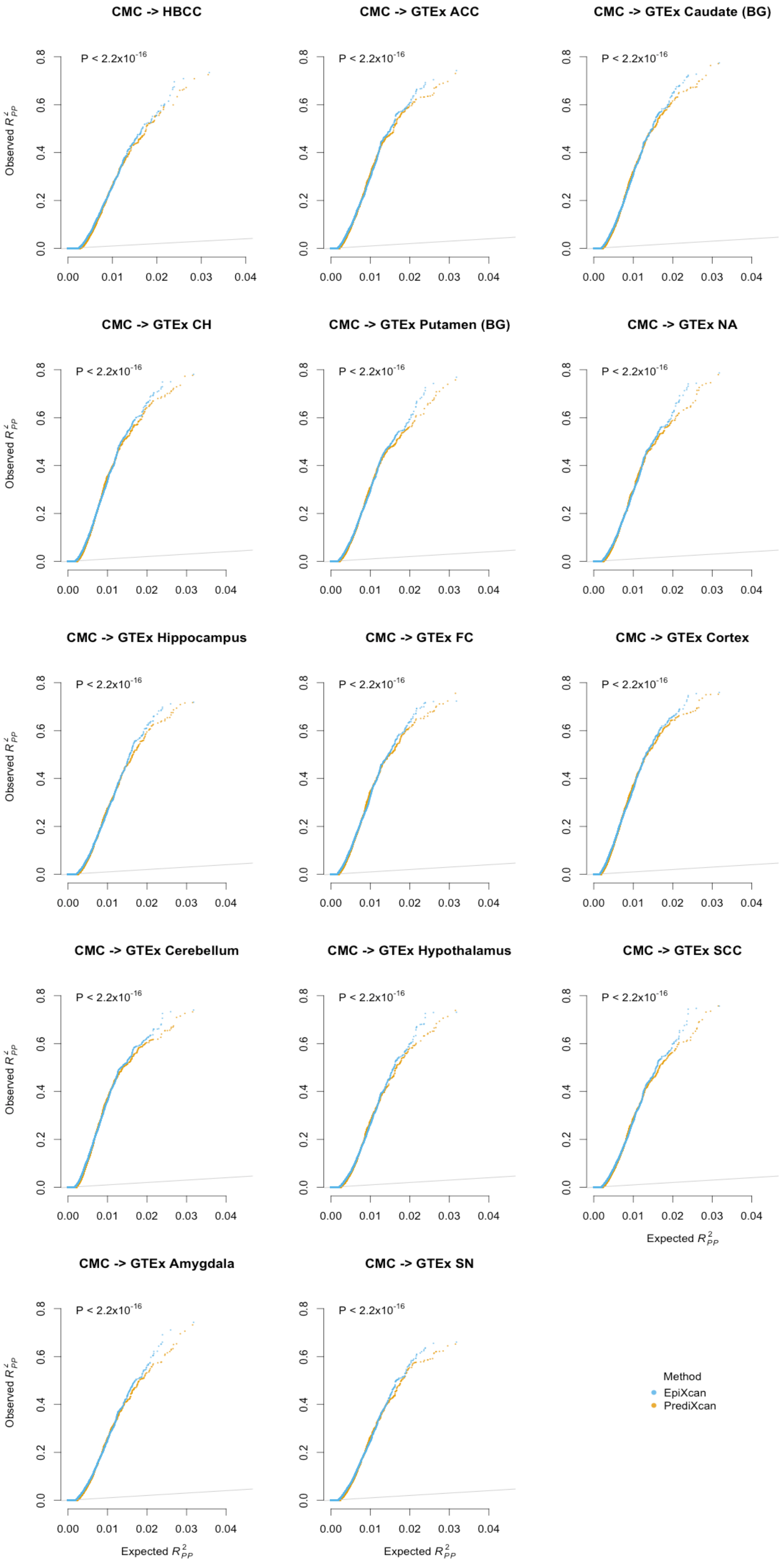

CMC -> HBCC
CMC -> GTEx ACC
CMC -> GTEx Caudate (BG)
CMC -> GTEx CH
CMC -> GTEx Putamen (BG)
CMC -> GTEx NA
CMC -> GTEx Hippocampus
CMC -> GTEx FC
CMC -> GTEx Cortex
CMC -> GTEx Cerebellum
CMC -> GTEx Hypothalamus
CMC -> GTEx SCC
CMC -> GTEx Amygdala
CMC -> GTEx SN
P < 2.2x10^{-16}
Observed R^2_{PP}
Expected R^2_{PP}
0.0
0.2
0.4
0.6
0.8
0.00
0.01
0.02
0.03
0.04
Method
EpiXcan
PrediXcan

Supplementary Figure 6. Comparison of model performance during prediction in independent datasets (R^2_{PP}) for brain tissues. QQ plots showing adjusted R^2_{PP} (between observed and predicted expression) for EpiXcan and PrediXcan. EpiXcan outperforms PrediXcan in gene expression imputation performance in independent test datasets. Models using CMC as training dataset are used to predict (->) gene expression of brain tissue from HBCC and 13 CNS regions from GTEx. For other relevant predictions, please refer to (**Supplementary Data 2**). Expected R^2_{CV} corresponds to the null distribution. p values indicate the significance of EpiXcan R^2_{PP} improvement over PrediXcan R^2_{PP} using Wilcoxon pairwise test. ACC: Anterior cingulate cortex; BG: basal ganglia; CH: Cerebellar Hemisphere; NA: Nucleus Accumbens; FC: Frontal Cortex; SCC: Spinal cord cervical; SN: Substantia Nigra.

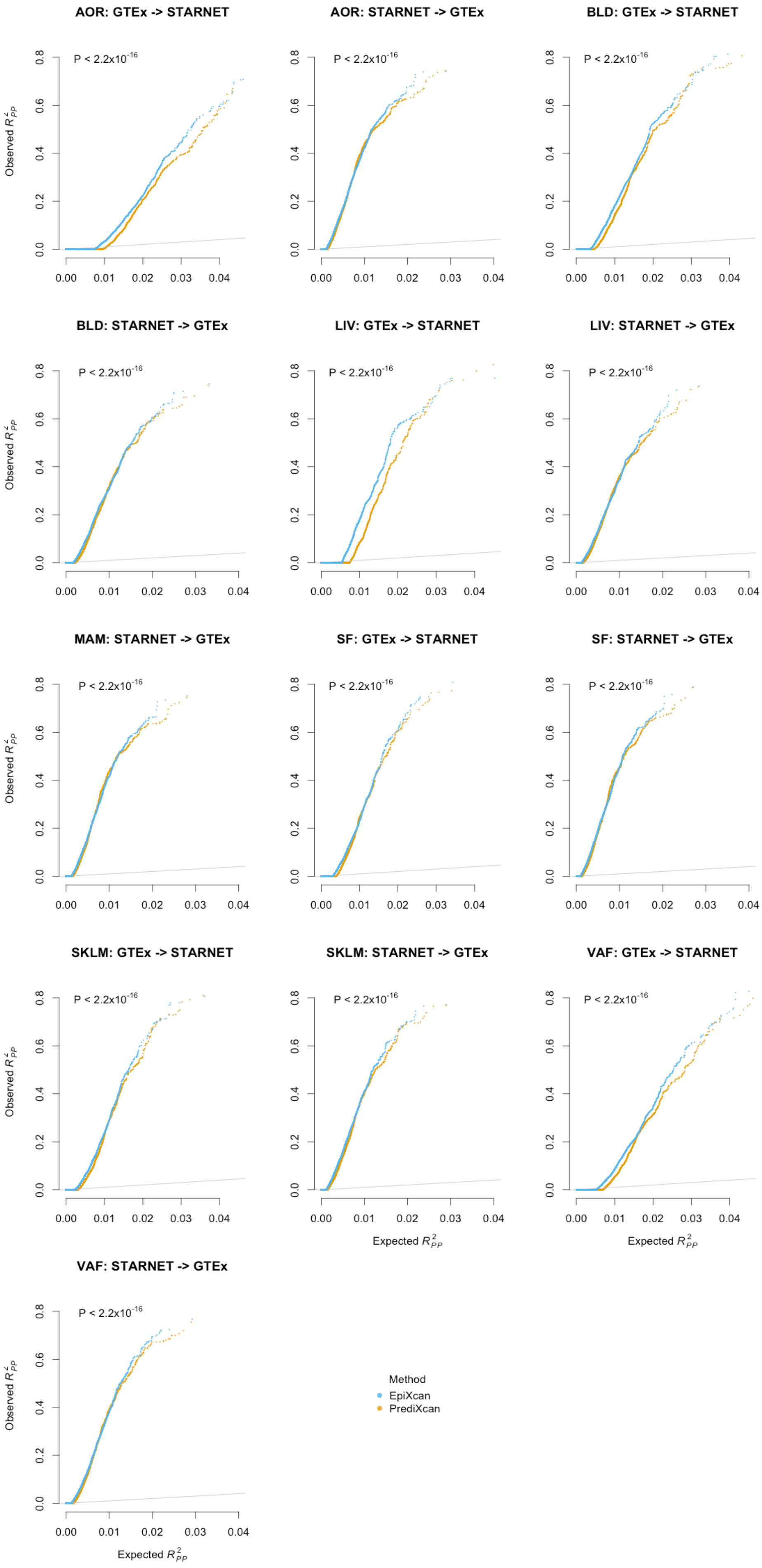

AOR: GTEx -> STARNET
AOR: STARNET -> GTEx
BLD: GTEx -> STARNET
BLD: STARNET -> GTEx
LIV: GTEx -> STARNET
LIV: STARNET -> GTEx
MAM: STARNET -> GTEx
SF: GTEx -> STARNET
SF: STARNET -> GTEx
SKLM: GTEx -> STARNET
SKLM: STARNET -> GTEx
VAF: GTEx -> STARNET
VAF: STARNET -> GTEx
P < 2.2x10^{-16}
Observed R^2_{PP}
Expected R^2_{PP}
Method
EpiXcan
PrediXcan

Supplementary Figure 7. Comparison of model performance during prediction in independent datasets (R^2_{PP}) for cardiometabolic tissues. QQ plots showing adjusted R^2_{PP} (between observed and predicted expression) for EpiXcan and PrediXcan. EpiXcan-trained gene expression models outperform PrediXcan-trained gene expression imputation models in test datasets. 'MAM: STARNET -> GTEx' denotes using model trained in STARNET mammary artery data to predict GTEx artery aorta transcriptome. For other relevant predictions, please refer to **Supplementary Data 2**. Expected R^2_{CV} corresponds to the null distribution. p values indicate the significance of EpiXcan R^2_{PP} improvement over PrediXcan R^2_{PP} using Wilcoxon pairwise test**.**

a

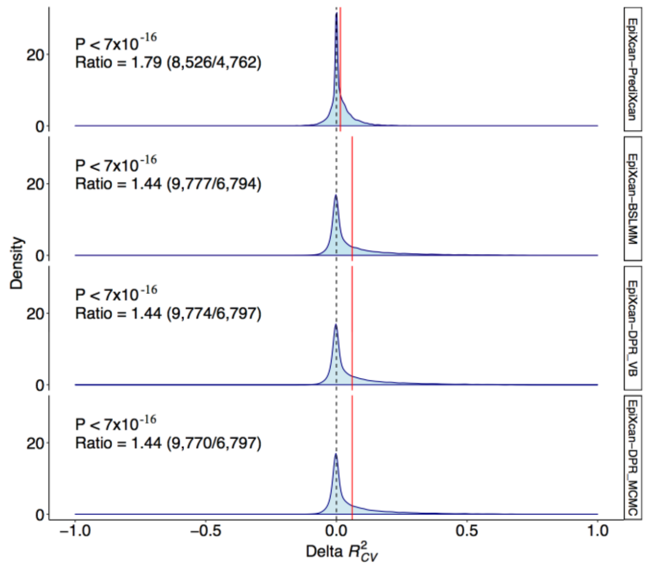

c

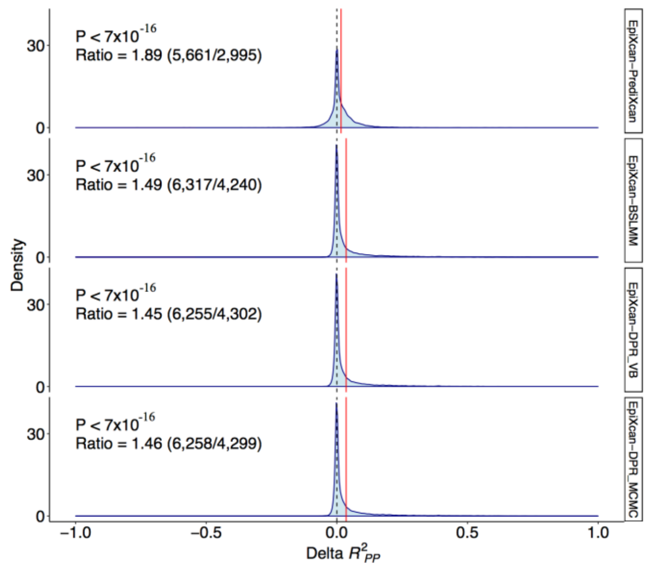

b

	Overall CPU cost (average time per gene; in seconds)	*p* value
EpiXcan	11.91	-
PrediXcan	12.99	$< 3 \times 10^{-16}$
BSLMM	2650.63	$< 3 \times 10^{-16}$
DPR_VB	11.53	$< 7 \times 10^{-16}$ *
DPR_MCMC	138.87	$< 3 \times 10^{-16}$

d

	Overall CPU cost (average time per gene; in seconds)	*p* value
EpiXcan	18.87	-
PrediXcan	20.71	$< 3 \times 10^{-16}$
BSLMM	3060.42	$< 3 \times 10^{-16}$
DPR_VB	11.97	$< 7 \times 10^{-16}$ *
DPR_MCMC	181.96	$< 3 \times 10^{-16}$

Supplementary Figure 8. Comparison of transcriptome imputation performance across five methods (EpiXcan, PrediXcan, BSLMM, DPR_VB, DPR_MCMC) in cross-validation and independent datasets. **(a-b) Comparison of R^2_{CV} and imputation computational duration using CMC datasets.** 16,572 genes are included in total. For PrediXcan comparison, we utilize adjusted R^2_{CV}**.** **(c-d) Comparison of R^2_{PP} and imputation computational duration when training in CMC and predicting HBCC brain tissue.** 10,557 HBCC genes are predicted and compared. In (a)/(c), we compare the R^2_{CV}/ R^2_{PP} by estimating the ΔR^2_{CV}/ ΔR^2_{PP} (EpiXcan R^2_{CV}/ R^2_{PP} minus Other method R^2_{CV} / R^2_{PP}). Across all the four other methods, (PrediXcan, BSLMM, DPR_VB, and DPR_MCMC) the overall delta value is positive, indicating that EpiXcan outperforms other methods. *p* value from one-sample sign test is provided to compare whether the shift of the $\Delta R^2_{CV}/\Delta R^2_{PP}$ values is different than zero (H$_0$: $\tilde{X} = 0$). The numbers in parentheses indicate the occasions where $\Delta R^2_{CV}/\Delta R^2_{PP}$ is higher in EpiXcan ($\Delta R^2_{CV}/\Delta R^2_{PP} > 0$; left number) and the other methods ($\Delta R^2_{CV}/\Delta R^2_{PP} < 0$; right number); ratio is estimated by dividing the occasions of $\Delta R^2_{CV}/\Delta R^2_{PP} > 0$ with $\Delta R^2_{CV}/\Delta R^2_{PP} < 0$. The red vertical line shows the mean of delta value. EpiXcan outperforms BSLMM and DPR in terms of transcriptomic imputation performance in both cross-validation and independent dataset testing (one-sample sign test *p* value $< 7 \times 10^{-16}$). Regarding the imputation computational duration, DPR_VB is faster than EpiXcan (*). On the other hand, DPR_MCMC is ~10× and BSLMM ~200× slower, respectively, when compared with EpiXcan. DPR was fitted with both the mean filed variational Bayesian (DPR_VB) and the Monte Carlo Markov Chain (DPR_MCMC) algorithms.

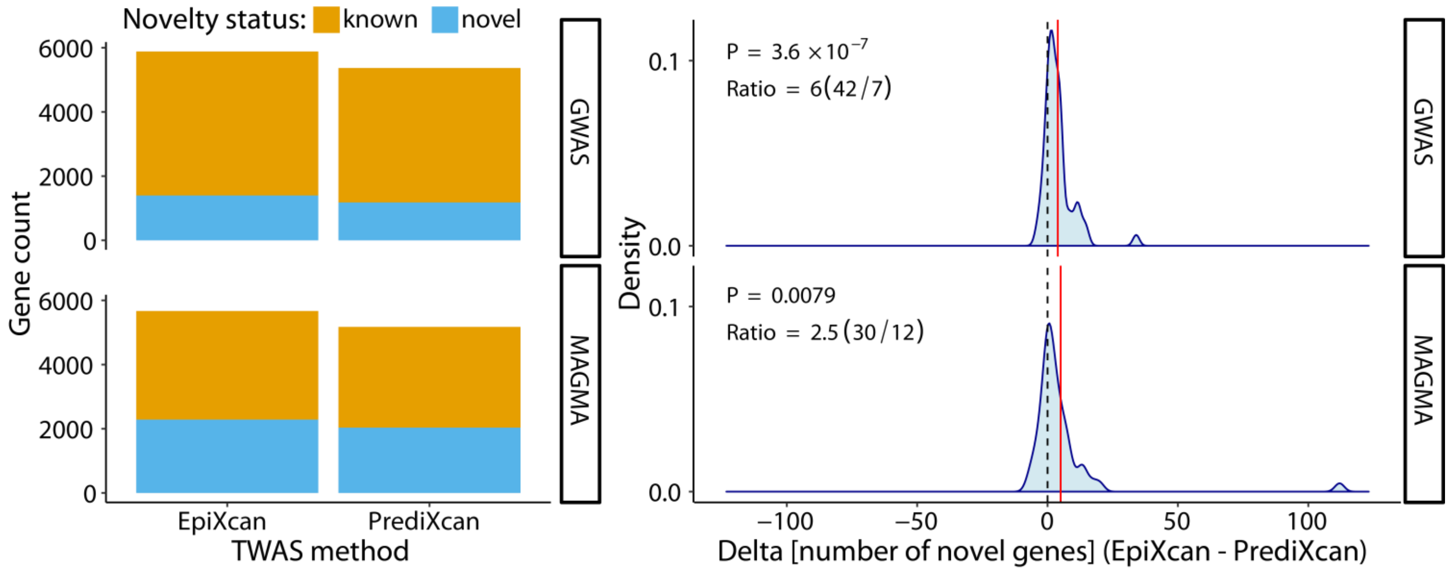

Supplementary Figure 9. EpiXcan identifies more "novel genes" than PrediXcan. Novel genes are those that, for a specific trait, no GWAS loci have reached genome-wide significance within 1Mb boundary on either side (GWAS), or are not identified by MAGMA gene analysis[27] after adjusting for multiple testing correction using the Benjamini-Hochberg method (FDR < 0.01). **Left panel:** Stacked bar plot showing total gene counts for known and novel genes identified by GWAS and MAGMA as above; overall, EpiXcan identifies more known and novel genes than PrediXcan. **Right panel:** Density plots depicting the distribution of the Δ[number of novel genes] (EpiXcan – PrediXcan) show that EpiXcan identifies more novel genes, which are within loci that did not reach genome-wide significance (GWAS) or were not identified by MAGMA (MAGMA), than PrediXcan (one-sample sign test p value = 3.6×10^{-7} and 0.0079 respectively, H_0: $\tilde{X} = 0$). Ratio is estimated by dividing the occasions of $\Delta\, n_{novel\ genes} > 0$ with $\Delta\, n_{novel\ genes} < 0$. The red vertical line shows the mean of delta values.

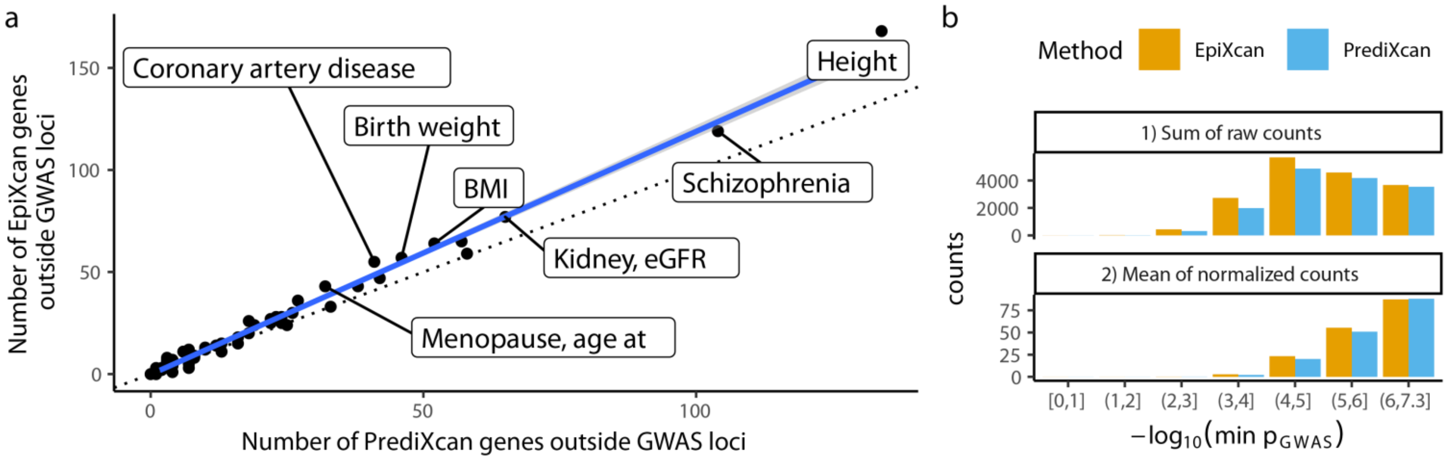

Supplementary Figure 10. Number of “novel genes” identified by EpiXcan and PrediXcan and the distribution of level of significance of associated SNPs in GWASs. (a) Scatter plot comparing the number of “novel genes” (not reaching genome-wide significance in GWAS) between EpiXcan and PrediXcan. The blue line corresponds to the regression line with 95% CI in grey. The dashed line is y=x. **(b)** Bar plots demonstrating the distribution of GWAS *p* values for SNPs corresponding to the “novel genes” for EpiXcan and PrediXcan. In the top panel, the sum of the SNPs for all traits are given for the respective *p* value bin. In the bottom panel, the SNP count was normalized for each GWAS by dividing the raw SNP count within the bin by the number of LD-independent genomic regions within the same bin; then the mean normalized counts per bin for all traits are plotted. The last bin (6,7.3] corresponds to *p* values within (10^{-6}, 5×10^{-8}).

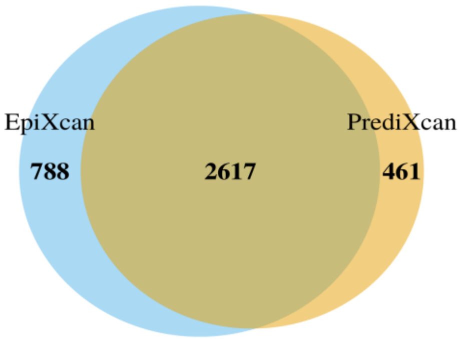

Supplementary Figure 11. EpiXcan uniquely identifies more significantly associated genes than PrediXcan. Venn diagram of genes with statistically significant gene-trait associations identified by both methods: EpiXcan and PrediXcan.

a

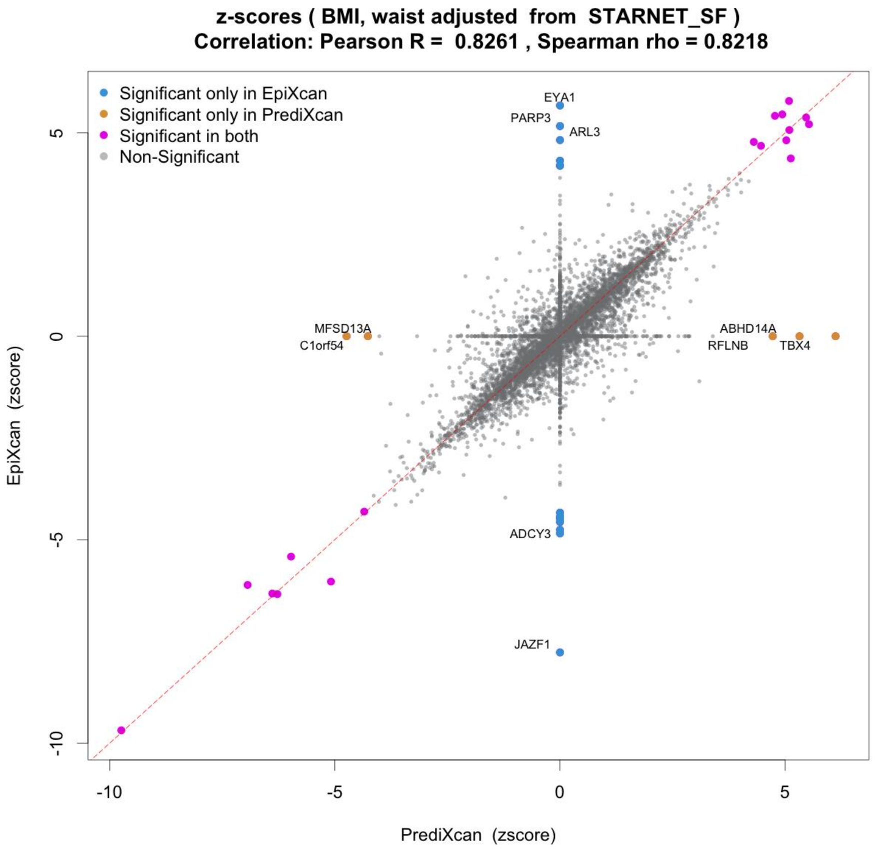
z-scores (BMI, waist adjusted from STARNET_SF)
Correlation: Pearson R = 0.8261 , Spearman rho = 0.8218
Significant only in EpiXcan
Significant only in PrediXcan
Significant in both
Non-Significant
EYA1
PARP3
ARL3
MFSD13A
C1orf54
ABHD14A
RFLNB
TBX4
ADCY3
JAZF1
EpiXcan (zscore)
PrediXcan (zscore)
-10
-5
0
5

b

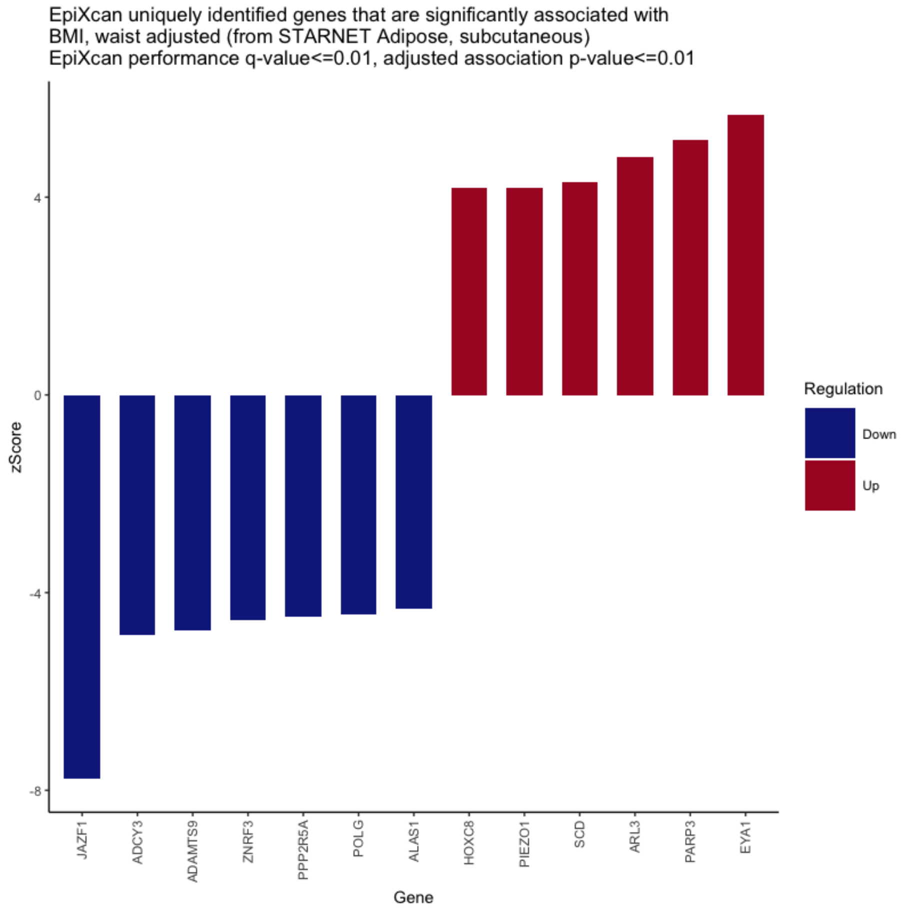
EpiXcan uniquely identified genes that are significantly associated with
BMI, waist adjusted (from STARNET Adipose, subcutaneous)
EpiXcan performance q-value<=0.01, adjusted association p-value<=0.01
zScore
4
0
-4
-8
Regulation
Down
Up
JAZF1
ADCY3
ADAMTS9
ZNRF3
PPP2R5A
POLG
ALAS1
HOXC8
PIEZO1
SCD
ARL3
PARP3
EYA1
Gene

Supplementary Figure. (a) Scatterplot demonstrating the correlation between z-score predictions by EpiXcan and PrediXcan for waist adjusted BMI in STARNET subcutaneous adipose tissue. Grey dots indicate genes that are identified by both methods but are not significant (FDR>1%). All colored dots (blue, orange and purple) denote genes that significantly associated with the trait (waist circumference adjusted BMI). Purple dots represent those significantly associated genes identified by both methods. Blue dots denote genes that are identified only by EpiXcan and orange dots those that are uniquely identified by PrediXcan; the top five genes based on [z] for each of the methods are indicated. Genes corresponding to uniquely identified genes by either method as above are not considered for the calculation of Pearson's and Spearman's correlation. **(b) Genes uniquely identified by EpiXcan for waist adjusted BMI in STARNET subcutaneous adipose tissue.** Z-scores of genes uniquely identified by EpiXcan corresponding to the blue dots in (a) panel. Respective (a) and (b) plots for all the 58 traits across the 14 tissues of the study can be found in our online repository http://icahn.mssm.edu/EpiXcan

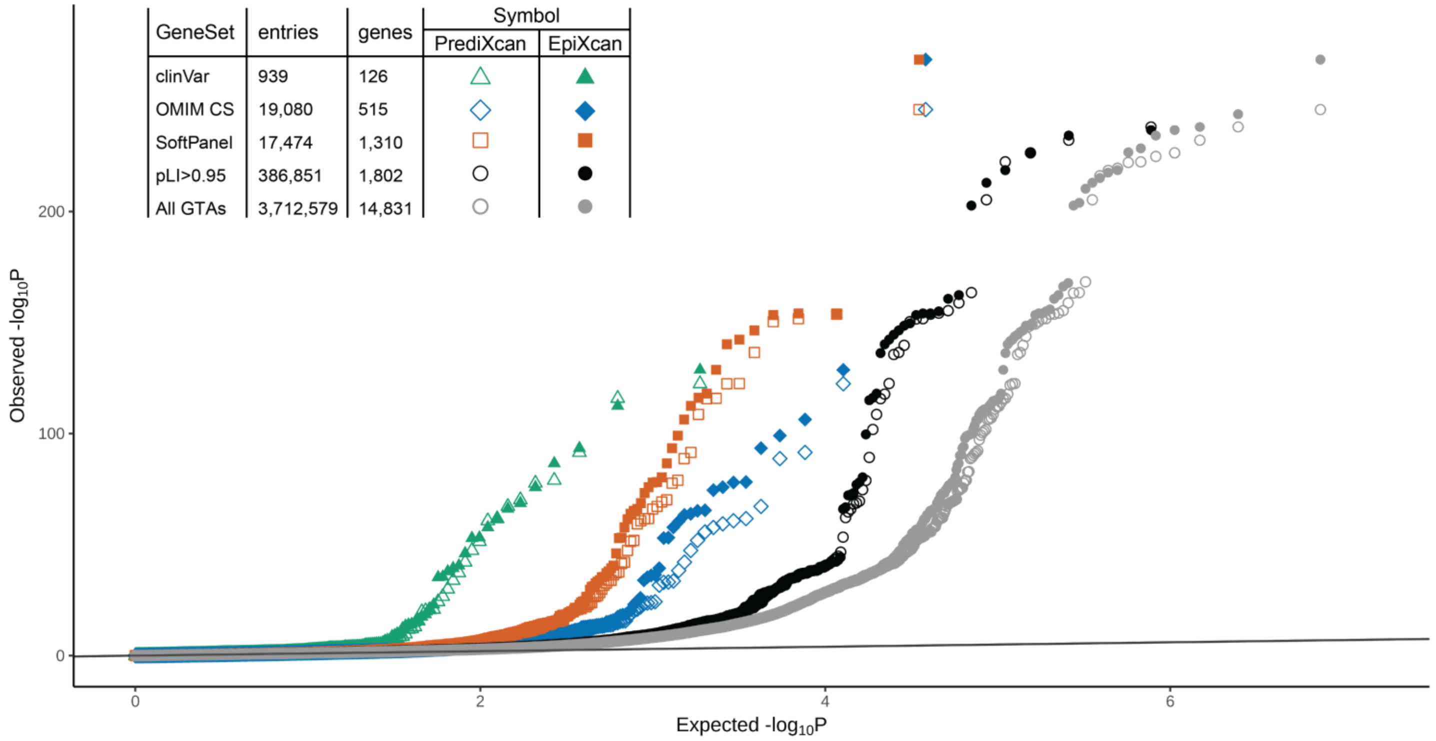

Supplementary Figure 13. Enrichment of clinically significant genes in EpiXcan and PrediXcan gene-trait associations. This is a Q-Q plot of the *p* values of the gene-trait associations for both EpiXcan and PrediXcan. For each human phenotype dataset (clinVar - green triangles, OMIM CS – blue diamonds, SoftPanel – orange squares) the entries are plotted for each gene, for all tissues but only for the traits for which the gene is associated in the respective dataset. In contrast, for the pLI > 0.95 dataset all points are plotted for all traits and tissues since there is not trait-specific information resulting in a higher number of entries. Since each entry represents a unique combination of gene, tissue (n=14) and trait (n=58), one gene can have up to 812 entries. All GTAs are also plotted for reference (grey dots). All Q-Q plots are statistically significantly different (Kolmogorov-Smirnov against all values – not shown).

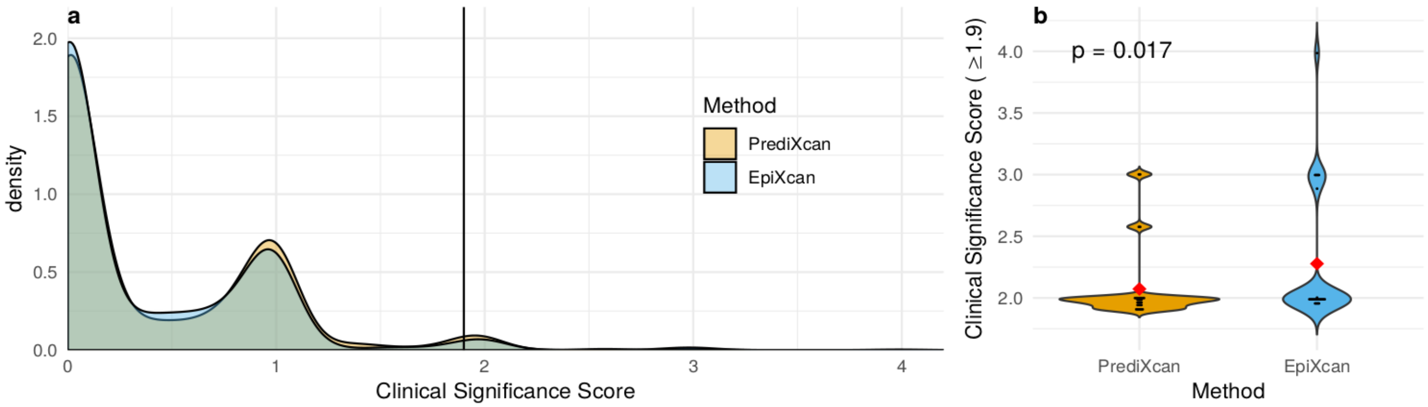

Supplementary Figure 14. Uniquely identified genes by EpiXcan are more likely to have clinical significance as verified by more than one clinical significance dataset. We define a clinical significance score (CSS) that ranges from 0 to 5, presence of a known gene-trait association in either of the datasets (ClinVar, OMIM CS, SoftPanel, MGD) counts for 1 point and then the pLI (0 to 1) is added to form the final score. (a) Density plot for the CSS of unique genes identified from EpiXcan and PrediXcan. The vertical line corresponds to CSS of 1.9 - the minimum score for a gene to have a gene-trait specific association corroborated by more than one datasets (eg. OMIM CS and pLI ≥ 0.9). (b) Violin plot for gene-trait associations with CSS ≥ 1.9. The red diamonds correspond to the mean of each distribution. *p* value was estimated with two-group Mann-Whitney U test.

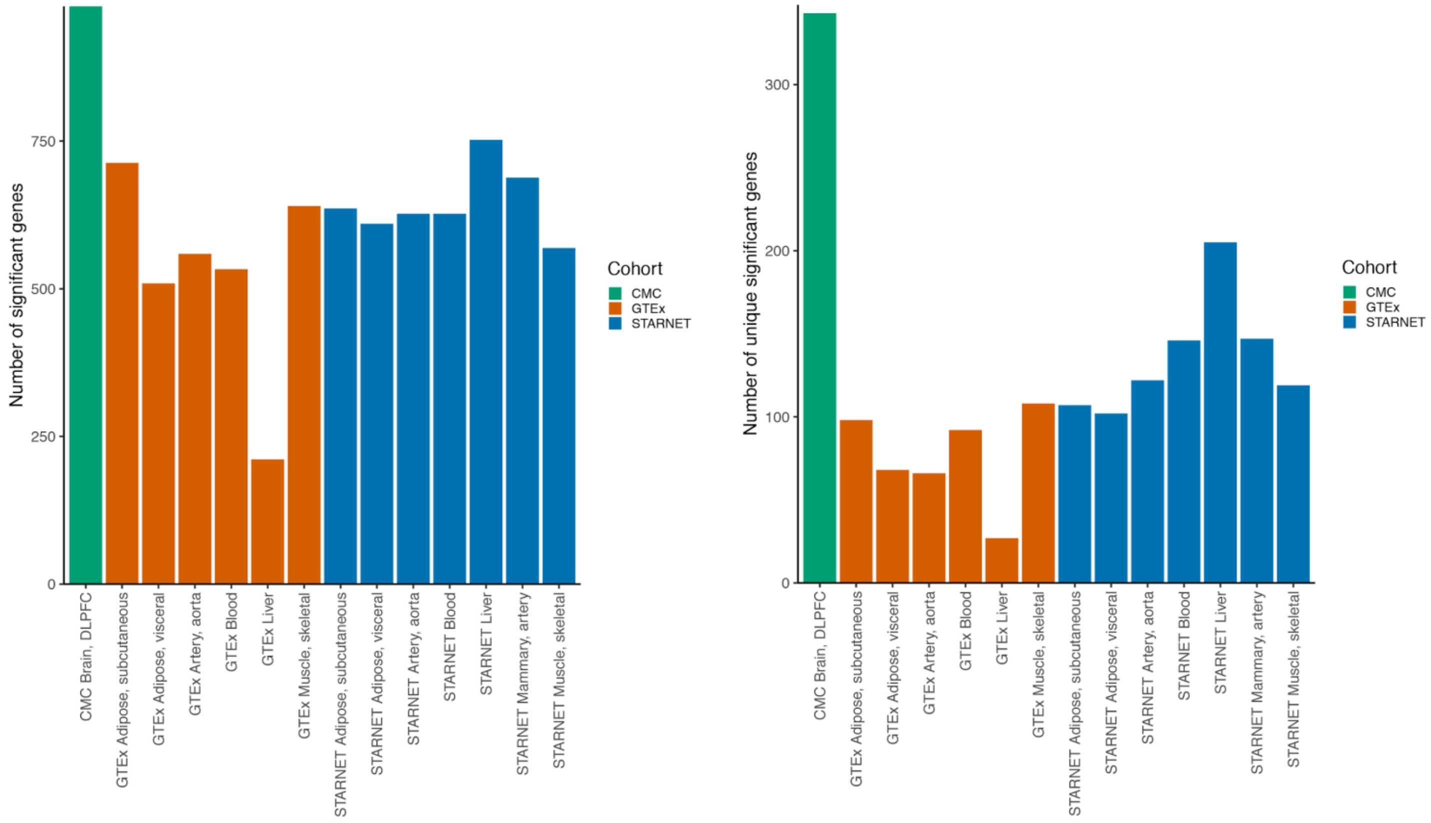

a **b**

Supplementary Figure 15. Gene contributions from different tissue models. (a) Contribution of significantly associated genes from each tissue in different cohorts. In total, there are 3,405 significant genes identified for all traits. (b) Contribution of unique (i.e. only identified for the trait in this specific tissue) significant genes from each tissue/cohort. GTEx liver tissue contributes less than others, which is reasonable, as it is the smallest sample size (n=130). For the size of studies, please refer to **Supplementary Table 1**.

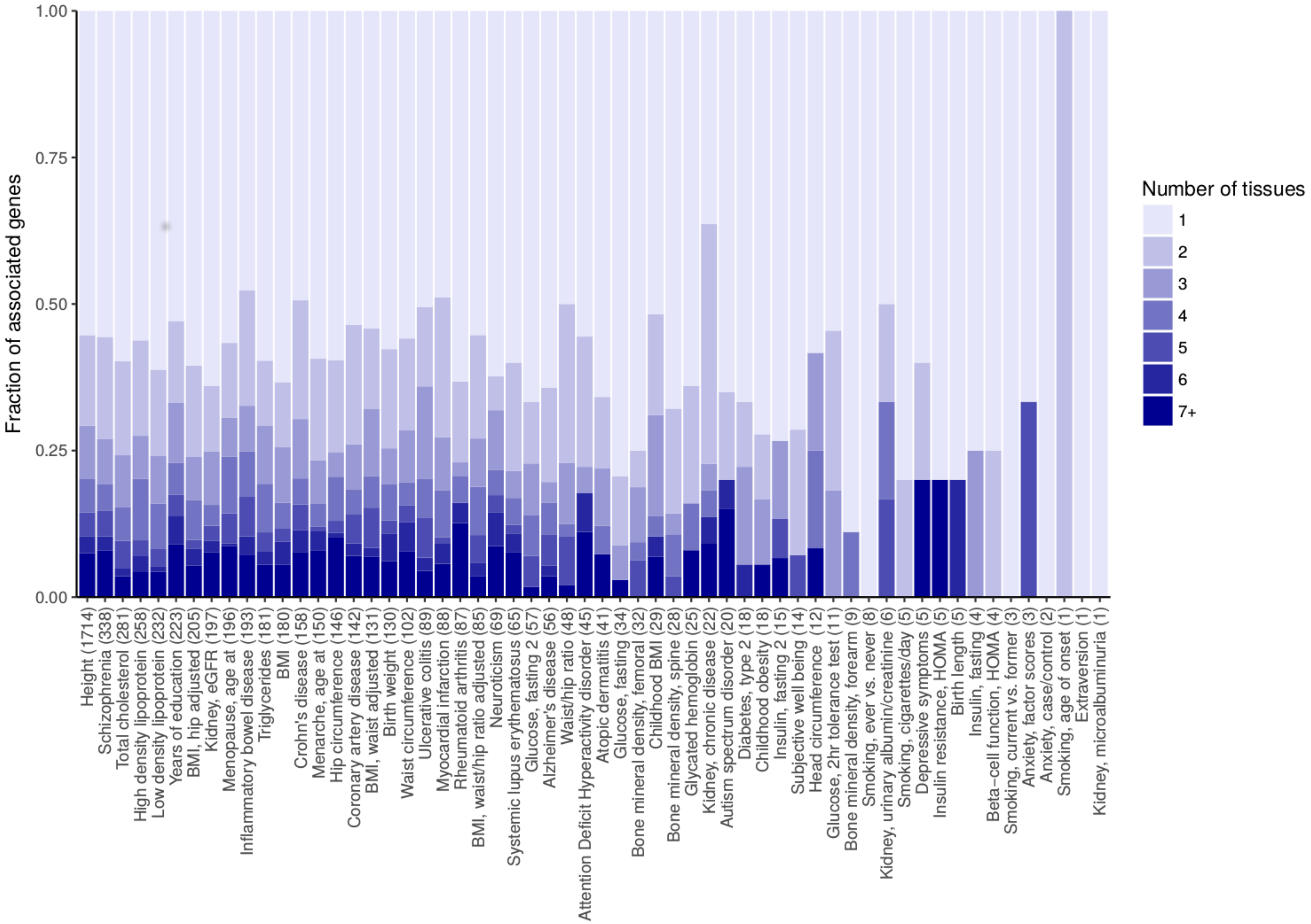

Supplementary Figure 16. Proportions of number of tissues contributing trait-specific gene trait associations. For each trait we show the fraction of associated genes that are contributed by a single tissue up to 7 or more tissues. Numbers in parentheses provide the total number of genes associated with each trait. We see that for most of the traits, more than 50% of the gene-trait associations are contributed from a single tissue.

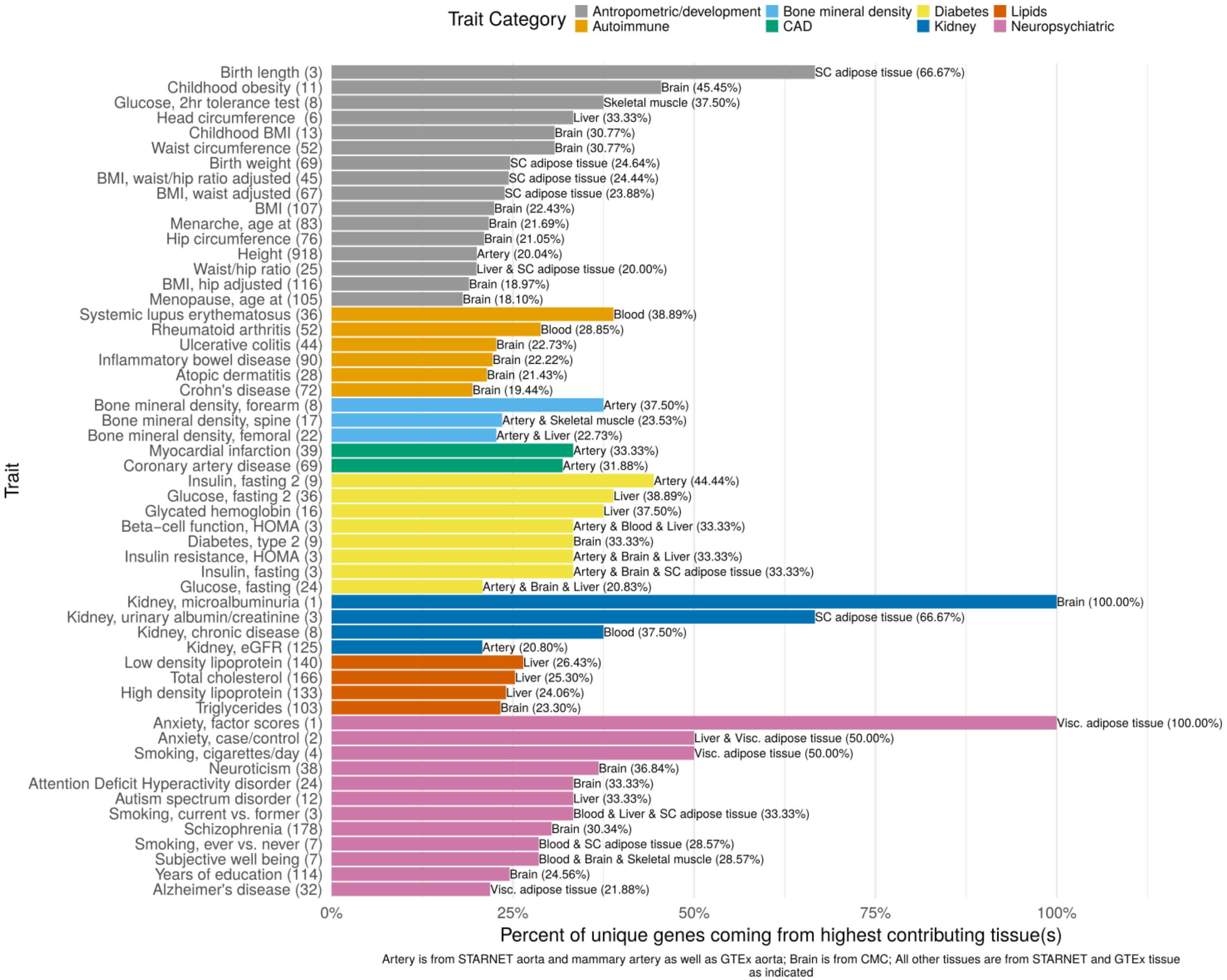

Supplementary Figure 17. **Percent of gene-trait associations contributed by top tissue type for each trait.** For almost all the traits, a large proportion of the unique associated genes come from one tissue type (32.98% ± 17.36%; mean ± SD). The digits in the parentheses indicate the number of genes being contributed by only one tissue type. The bars denote percentages of unique genes coming from highest contributing tissues for all traits. If more than one tissue contributes the same top number of unique genes, all tissue type names are provided (separated by “&”).

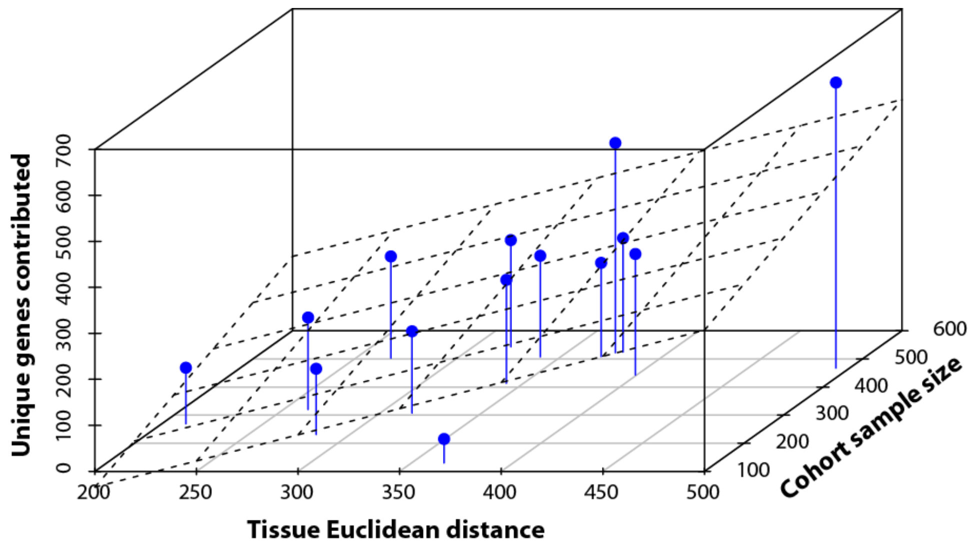

Supplementary Figure 18. Cohort sample size and tissue dissimilarity explain most of the variation in the number of unique genes contributed by each tissue model. 3D scatter plot demonstrating how tissue dissimilarity (as estimated by the average Euclidean distance of the significant *z* scores versus all other tissues) and cohort sample size correlate with the number of unique genes contributed. Each blue dot represents one of the 14 tissue models of the study, the blue line projections (for each tissue i: $\langle x, y, z \rangle = \langle x_i, y_i, z_i \rangle + t\langle 0, 0, -z_i \rangle$) help create sense of depth for visualization. The plane corresponding to the multiple linear regression model ($N_{\text{unique genes}}$ = -300.78 + 1.13 × "Tissue Euclidean distance" + 0.39 × "Cohort sample size", adjusted R^2 = 0.52, p value = 0.007) is drawn with dotted lines.

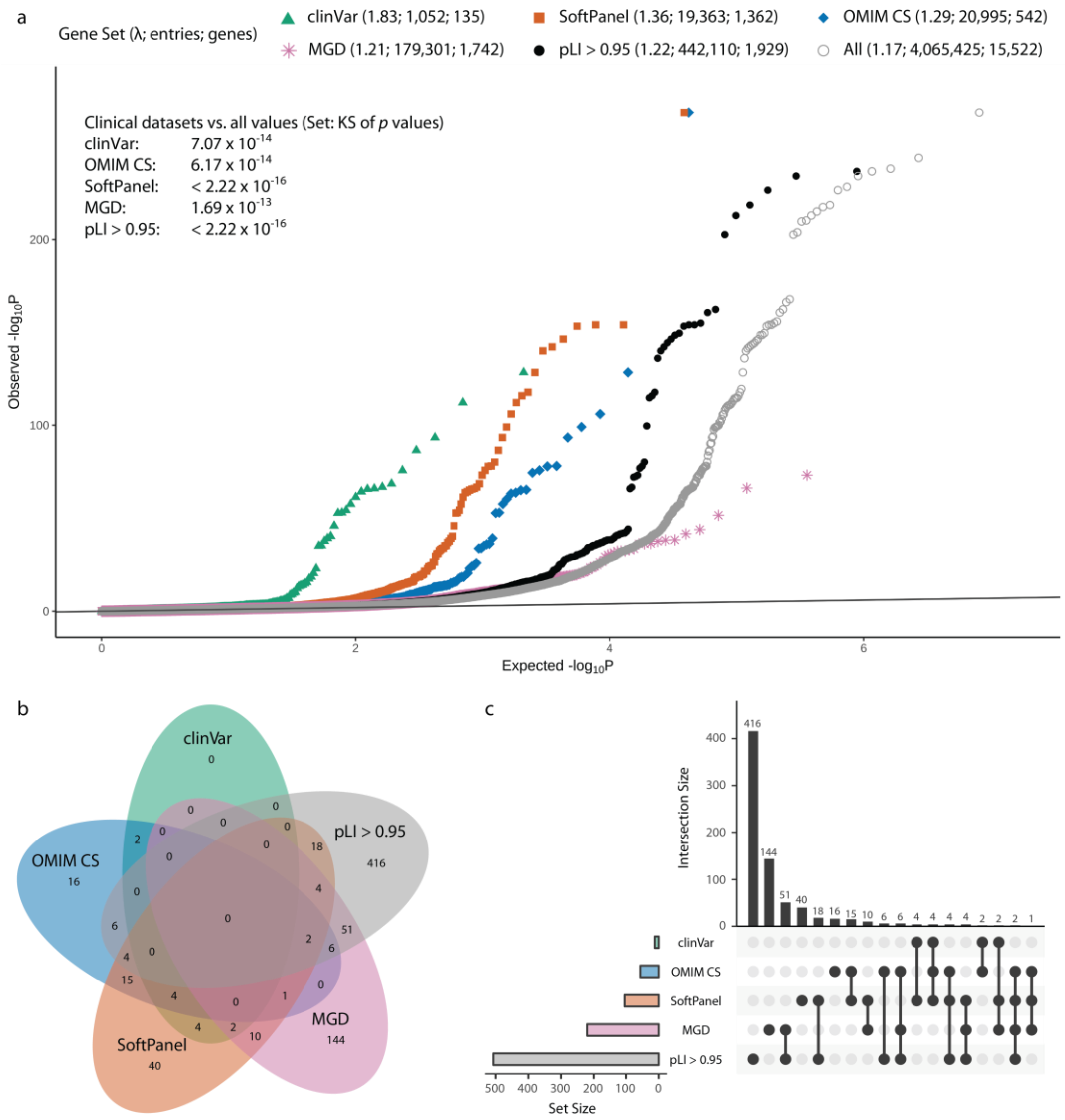

Supplementary Figure 19. Enrichment of associated clinically relevant genes in EpiXcan gene-trait associations. (a) Q-Q plot of the *p* values of the gene-trait associations for EpiXcan. For human phenotype datasets (**Supplementary Data 10**; clinVar - green triangles, OMIM CS – blue diamonds, SoftPanel – orange squares) the entries are plotted for each gene for all tissues, but only for the traits for which the gene is associated in the respective dataset; phenotypic severity is lower in OMIM CS which identifies clinical signs similar to the trait and higher in clinVar which, in most cases, corresponds to a Mendelian (monogenic) form of the trait. The entries are plotted similarly for the MGD dataset (MGD – pink stars) which identifies ortholog mouse genes that are associated with mouse phenotypes that are in the same broad phenotypic category as the human trait (**Supplementary Data 10**). In contrast, for the pLI > 0.95 dataset (black circles), all points are plotted for all traits and tissues since there is no trait-specific information. For reference, the *p* value distribution of all predictions is given (grey circles). Since each entry represents a unique combination of gene, tissue (n=14) and trait (n=58), one gene can have up to 812 entries. Genomic inflation factors (λ) are given in the legend at the top and Kolmogorov-Smirnov *p* values (against distribution of all values) are given in the custom annotation (top left). **Venn diagram (b) and matrix layout (c) for all the intersections of genes with statistically significant gene-trait associations that belong to at least one of the datasets in (a).**

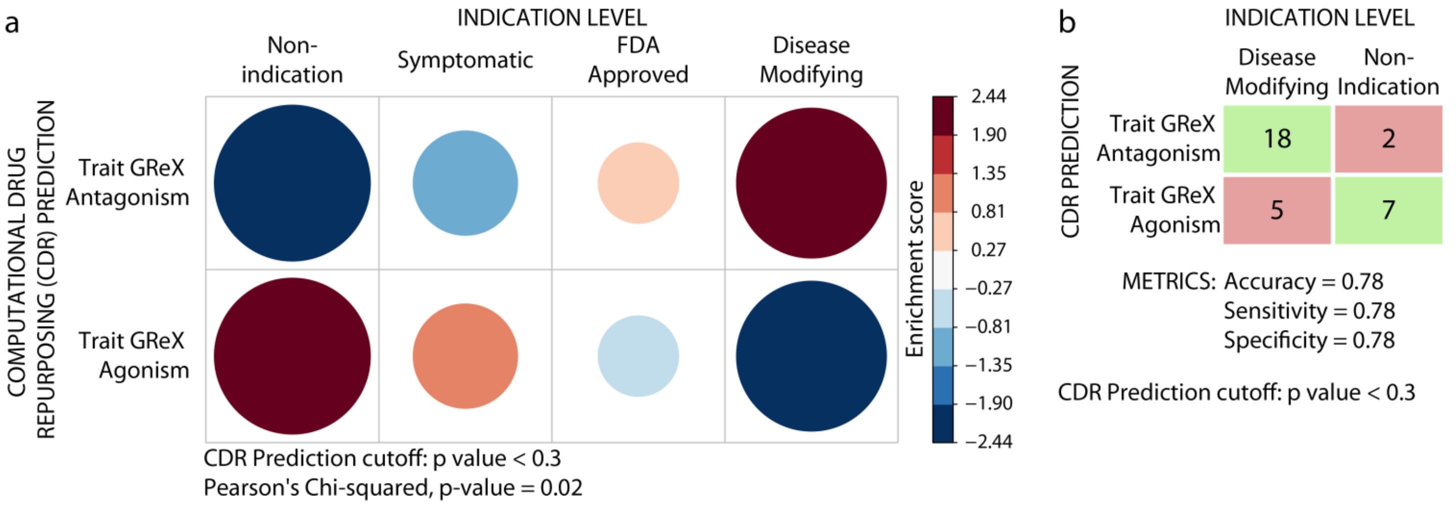

Supplementary Figure 20. **Assessment of computational drug repurposing (CDR) pipeline.** For the CDR compound predictions, we only consider predictions that have a nominal p value < 0.3. CDR predictions with a negative connectivity score (CS) are predicted to reverse the genetically-driven gene expression changes for the trait of interest (Trait GReX Antagonism – potentially therapeutic) and with a positive CS are predicted to drive them in the same direction (Trait GReX Agonism – potentially harmful). We split real-world indications into four groups of increasing efficacies: i) non-indication: "a drug that neither therapeutically changes the underlying or downstream biology nor treats a significant symptom of the disease", ii) symptomatic: "a drug that treats a significant symptom of the disease", iii) FDA-approved: a subset of the medications that are currently used (many are used “off-label”) that have been shown to have at least symptomatic indication, and iv) disease modifying: "a drug that therapeutically changes the underlying or downstream biology of the disease". **(a)** 2D enrichment matrix showing the relationship between predicted effect of compounds on a given trait compared to real-world indications level. We see that the higher the level of indication, the higher the level of enrichment for potentially therapeutic CDR predictions and, conversely, the lower the level of indication, the higher the level of enrichment for potentially harmful CDR predictions (Pearson’s χ^2 test of independence, p value = 0.02). The enrichment score is standardized residuals from the χ^2 test[28]. **(b)** Confusion matrix demonstrating the different types of correct (in green) and false classifications (in red) by the CDR pipeline. Compounds predicted to reverse trait-specific changes (trait GReX antagonism) are more likely to be disease modifying for the trait (conditional maximum likelihood estimator odds ratio 11.37, Barnard’s unconditional test 2-tailed p value = 0.006). Barnard’s unconditional test was performed because the expected values in the matrix did not meet the criteria for performing Pearson’s χ^2 test. The metrics of predicting disease modifying treatments (with non-indication as the negative control) are given below the table. The data used to generate this Figure are given in **Supplementary Table 2.**

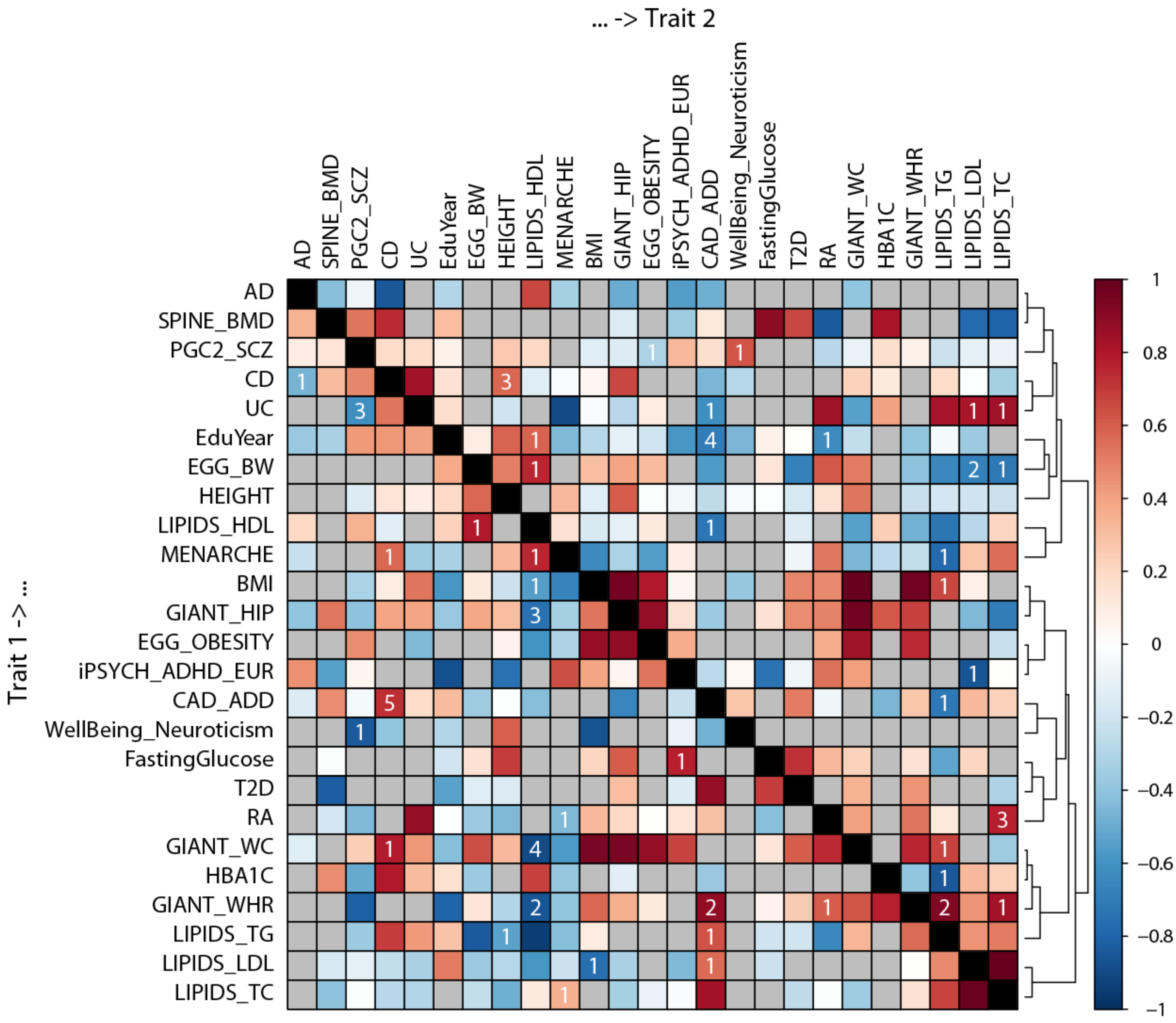

Supplementary Figure 21. **Putative trait causal relationships based on imputed transcriptomes.** Bi-directional regression analysis was performed for the predicted transcriptomes of all tissues for all significantly correlated trait pairs (r_g and r_{GReX}, q value ≤ 0.05) and the conditional estimates $\rho_{\text{Trait 1} \mid \text{Trait 2}}$ are shown as color-coded squares in this 2D matrix (blue = protective = -1; red = causal = 1). Trait pairs that are not significantly correlated are shown with grey. For the trait pairs that displayed a significant difference (p<0.05, Welch's t test) in their conditional estimates (Trait 1 -> Trait 2 *vs.* Trait 2 -> Trait 1), we use white labels to denote the number of tissues where this difference was observed. Dendrogram on the right edge is shown from Ward hierarchical clustering.

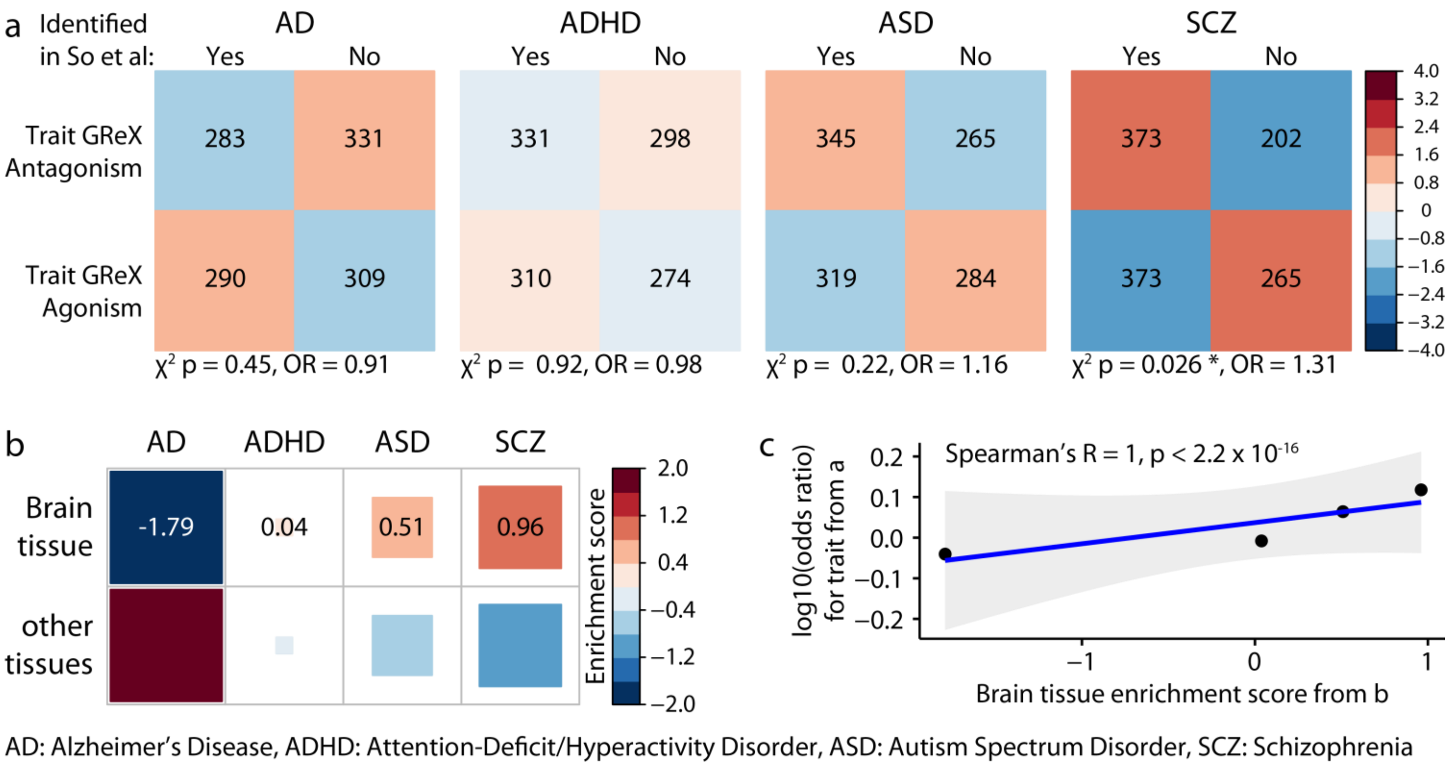

Supplementary Figure 22. Drug repurposing prediction comparisons with So *et al.*[25] **(a)** Confusion matrices with enrichment annotation accounting for the concordant and non-concordant predictions between our CDR pipelines across evaluated traits. Above matrices are provided for all common traits between the two studies: *(1)* Alzheimer's Disease – OR = 0.91, *p* value = 0.45, *(2)* Attention-Deficit/ Hyperactivity Disorder – OR = 0.98, *p* value = 0.92, *(3)* Autism Spectrum Disorder – OR = 1.16, *p* value = 0.22, and *(4)* Schizophrenia – OR = 1.31, *p* value = 0.026. OR: conditional maximum likelihood estimate odds ratio; *p* value: Pearson's χ^2 test of independence *p* value. Compounds predicted to normalize schizophrenia gene-trait signatures (Trait GReX antagonism) are more likely to be provided as top therapeutic candidates in So *et al* but this is not true for the other traits under evaluation; the ORs are used in **c**. **(b)** EpiXcan gene-trait association (GTA) 2D enrichment matrix showing the relationship between the brain tissue prediction model (CMC) and the evaluated traits. The enrichment score corresponds to the standardized residual for each tissue-trait pair (as described for **Figure 3b**) and, in this case, all other tissues except CMC are pooled together. The brain tissue enrichment scores for the evaluated traits are provided in the matrix and are used in **c**. Traits are ordered based on Ward hierarchical clustering **(c)** Scatter plot of brain tissue enrichment scores from **b** and $\log_{10}$OR from **a**. The data suggest that the higher the brain tissue enrichment score for the trait, the higher the likelihood that our results will be concordant with So *et al*. (Spearman's $\rho = 1$, *p* value $< 2.2 \times 10^{-16}$). For the whole analysis, the traits "anxiety, case/control" and "anxiety, factor scores" are excluded because they yield no significant GTAs in CMC (brain tissue) in our study after adjusting the *p* values for multiple correction as described in the main text.

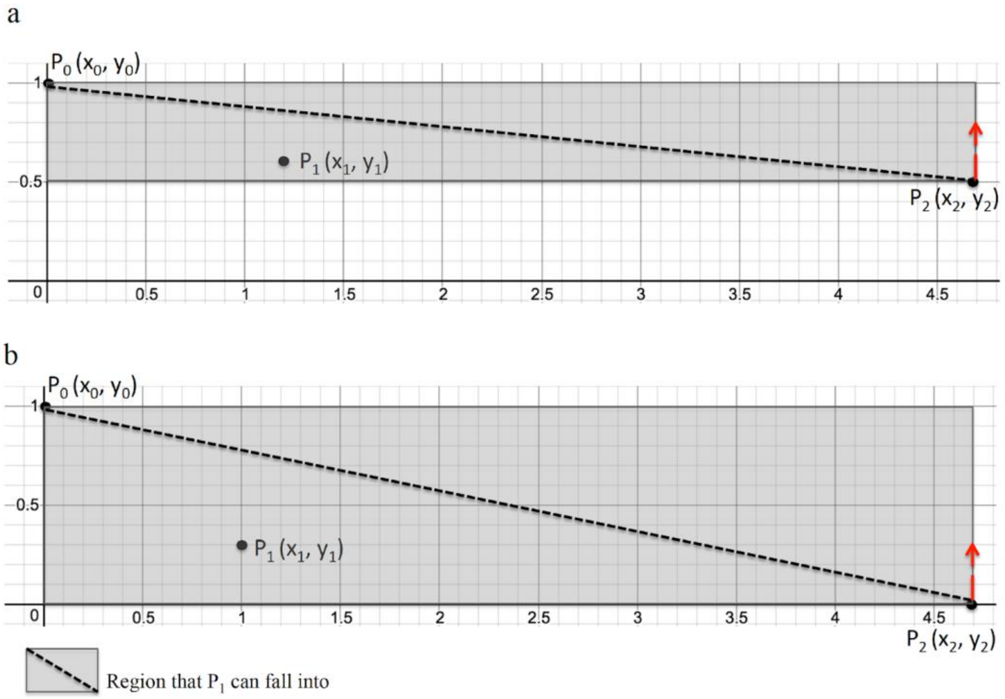

Supplementary Figure 23. Demonstration of the process to determine the rescaling function by second order Bézier approximation. By using 2nd order Bézier curve, we need to decide only one optimal intermediate control point P_1. P_0 is (0, 1), and P_2 has coordinate (x_2, y_2), where x_2 is the maximal value of all the priors and y_2 is fixed in each window. Red dashed arrow points to the direction of shifting the window. (a) The window of the process where y_2 = 0.5. (b) The first window of the process where y_2=0.

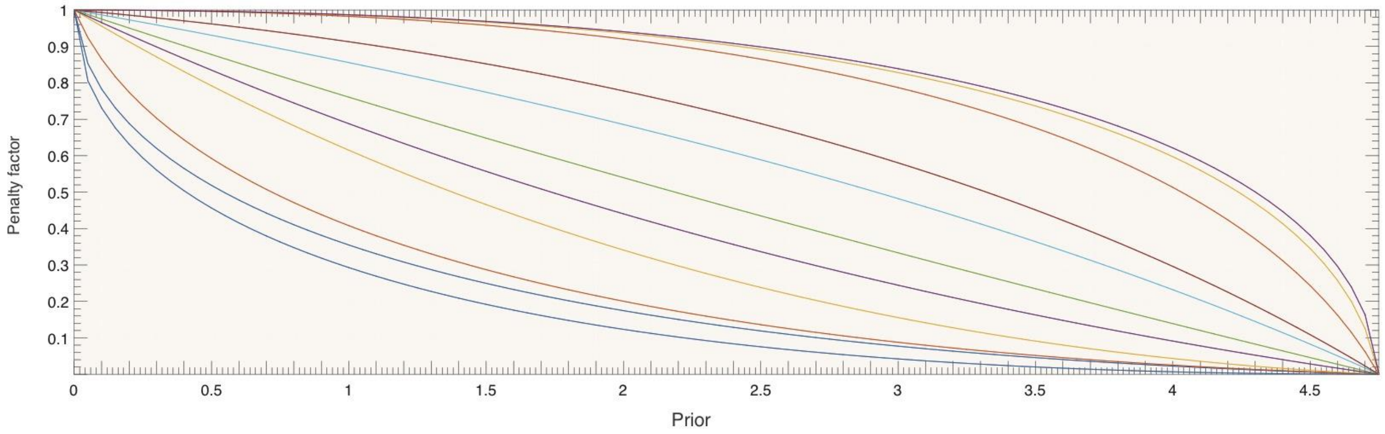

Supplementary Figure 24. Examples of candidate rescaling's in one illustrative window. The starting and end control points determine the start and end of the curve, but the intermediate control point determines the shape of the rescaling function. Every grid point in the window is one candidate intermediate control point, based on which different candidate interpolations are obtained. Since we use quadratic (second order) interpolation functions the direction of the curve can only change once thus creating either concave down or concave up curves in this set of examples. Higher order Bézier interpolation functions may have combination of concave down and concave up shapes.

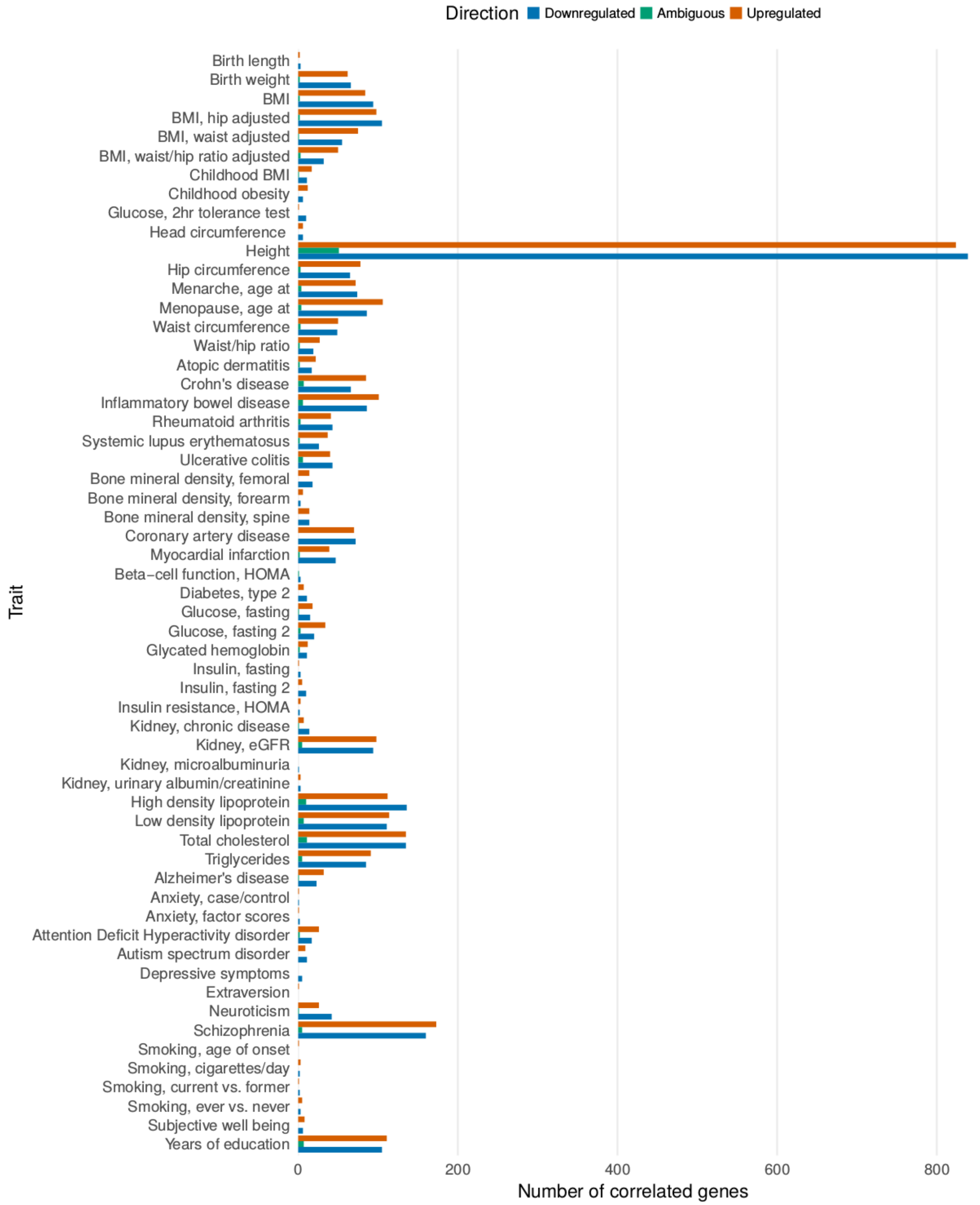

Supplementary Figure 25. Genes predicted to be up- or down-regulated in different tissues for each trait. A gene is regarded as up-/down-regulated for a given trait if z-score are positive/negative in the majority of the tissues. If there are equal numbers of tissues predicting up- or down-regulation, then the expressional change is regarded as ambiguous. Only genes that are significantly associated with traits are considered.

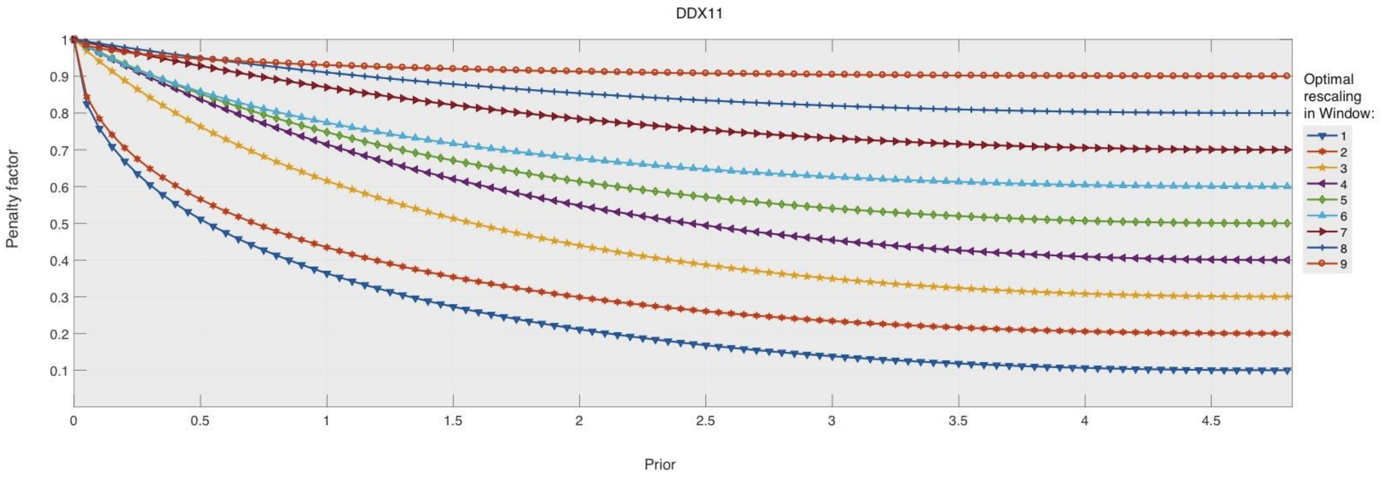

Supplementary Figure 26. Optimal re-scaling in each of the windows for *DDX11* simulations. The rescaling in window 7 was found to be the best performing one.

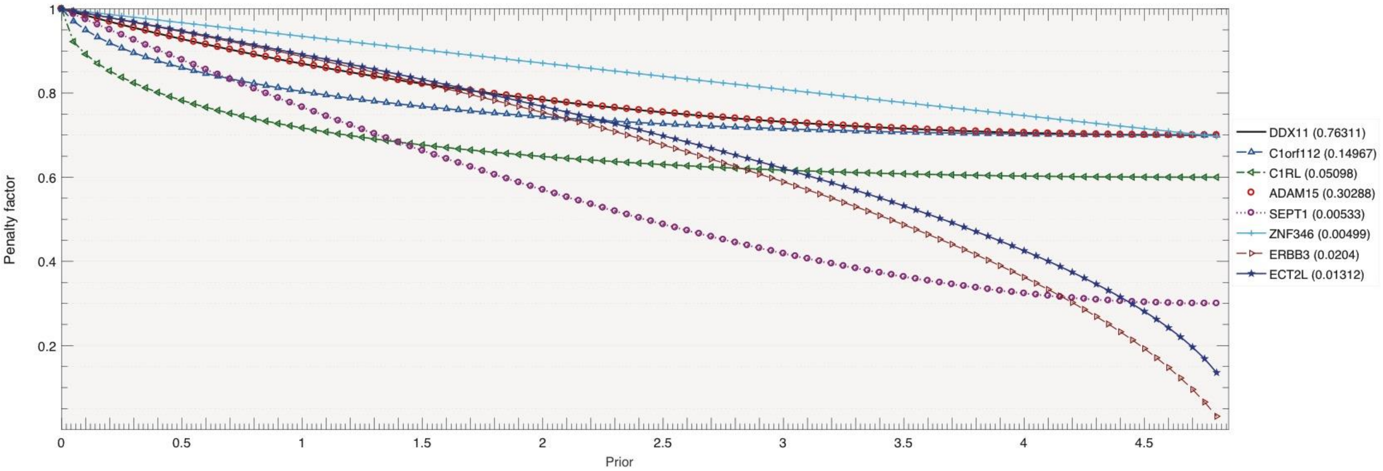

Supplementary Figure 27. **Optimal re-scaling regarding different simulations.** This plot shows the best re-scaling for each of the simulations regarding several genes. Numbers in brackets indicate the R^2_{CV} of the genes. Then the re-scalings are evaluated by the overall R^2_{CV} and the mapping(s) from *DDX11* (*ADAM15*) is assessed to be optimal. The mappings from these two genes are exactly the same and, more importantly, the overall R^2_{CV} of the rescaling is superior to others. Overall, R^2_{CV} is calculated based upon the real CMC data. For each re-scaling, we compare the overall R^2_{CV} with that of PrediXcan using Wilcoxon test, and the re-scaling with most significant *p* value is considered the most optimal.

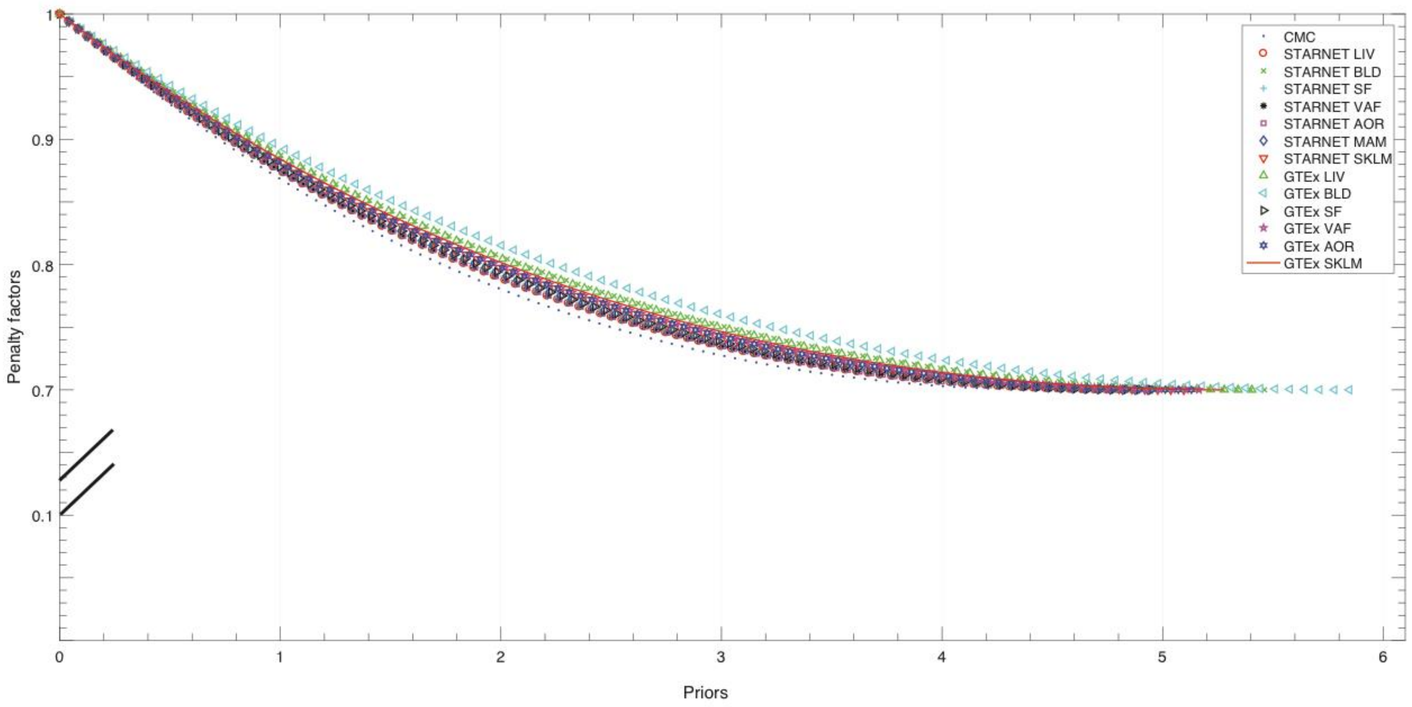

Supplementary Figure 28. **Optimal re-scaling for each of the data sets.** Due to different prior ranges of each data set, the re-scaling functions vary.

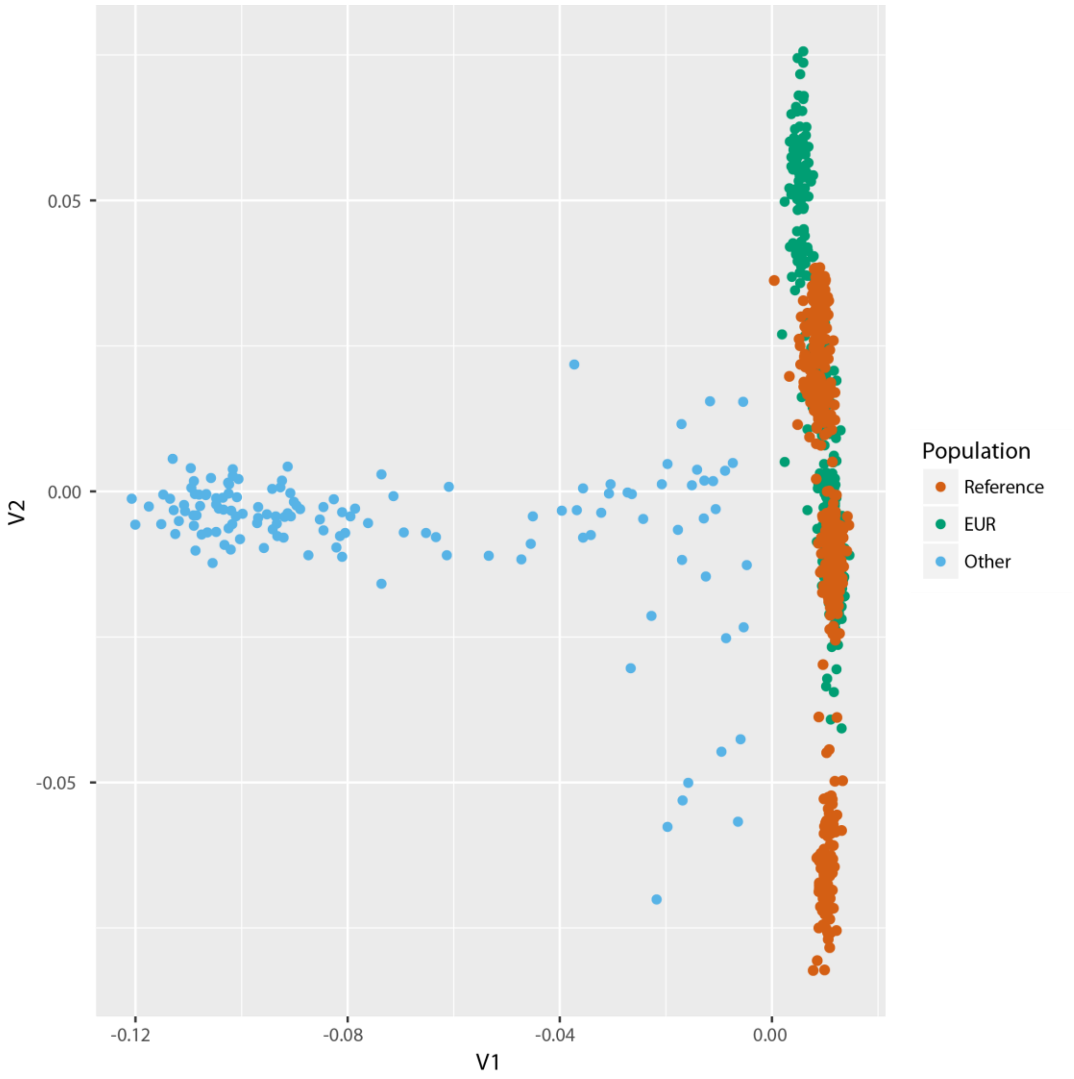

Supplementary Figure 29. Principle analysis to identify European population for CMC. The samples are merged with 1000 genome EUR subset (~500 samples) and principal component analysis (PCA) is performed using ~30,000 pruned and thinned variants. We plot first and second principal components and define an ellipsoid based on 1KG EUR samples (orange dots in the plot). Those that lie 6 SD away from the center of this ellipsoid are considered as genetic outliers (blue dots) and removed. Green dots denote EUR samples that were kept for downstream analysis.

Supplementary Tables

Supplementary Table 1. Overview of gene expression and genotype datasets included in current study. "Number of Genes" is the number of genes that are detectable in each dataset. "Predictor" indicates the datasets used to train PrediXcan and EpiXcan models. "Observed" indicates the datasets used to verify accuracy of predictions.

**Cohorts**	**Tissue**	**Sample Size**	**Number of Genes**	**Predictor**	**Observed**
**STARNET**	SF	543	14119	✓	✓
**STARNET**	MAM	524	15458	✓	✓
**STARNET**	LIV	522	13875	✓	✓
**STARNET**	AOR	508	16214	✓	✓
**STARNET**	SKLM	507	12544	✓	✓
**STARNET**	VAF	503	14964	✓	✓
**STARNET**	BLD	443	12843	✓	✓
**GTEx**	SKLM	413	14594	✓	✓
**GTEx**	SF	320	14720	✓	✓
**GTEx**	BLD	307	14010	✓	✓
**GTEx**	VAF	269	14720	✓	✓
**GTEx**	AOR	231	14711	✓	✓
**GTEx**	LIV	130	14536	✓	✓
**CMC**	Brain, DLPFC	467	16423	✓	
**HBCC**	Brain	280	12615		✓
**GTEx**	Brain, Cerebellum	154	14671		✓
**GTEx**	Brain, Caudate basal ganglia	144	14690		✓
**GTEx**	Brain, Cortex	136	14684		✓
**GTEx**	Brain, Nucleus accumbens basal ganglia	130	14689		✓
**GTEx**	Brain, Cerebellar Hemisphere	125	14648		✓
**GTEx**	Brain, Frontal Cortex	118	14675		✓
**GTEx**	Brain, Hippocampus	111	14694		✓
**GTEx**	Brain, Putamen basal ganglia	111	14671		✓
**GTEx**	Brain, Anterior cingulate cortex	109	14675		✓
**GTEx**	Brain, Hypothalamus	108	14701		✓
**GTEx**	Brain, Amygdala	88	14682		✓
**GTEx**	Brain, Spinal cord cervical	83	14720		✓
**GTEx**	Brain, Substantia nigra	80	14693		✓

Supplementary Table 2. Computational drug repurposing (CDR) pipeline validation. Comparison of computational drug repurposing pipeline predictions with real-world indications for trait-compound pairs.

Trait	Compound	ConnectivityScore	Pvalue	FDR	Indication
**Alzheimer's disease**	memantine	-0.13077489	0.187305819	1	disease modifying
**Alzheimer's disease**	selegiline	-0.152043071	0.066308164	1	symptomatic
**Atopic dermatitis**	dimenhydrinate	-0.1761405	0.071838772	0.994807846	disease modifying
**Atopic dermatitis**	fluocinonide	0.132623112	0.263270946	0.994807846	disease modifying
**Atopic dermatitis**	methylprednisolone	-0.146502884	0.208472161	0.994807846	disease modifying
**Atopic dermatitis**	methylprednisolone	-0.146502884	0.208472161	0.994807846	FDA approved
**Atopic dermatitis**	mometasone	-0.130404542	0.299838588	0.994807846	disease modifying
**Attention Deficit Hyperactivity disorder**	clonidine	0.126502326	0.118583297	0.985994892	symptomatic
**Attention Deficit Hyperactivity disorder**	guanfacine	-0.111252342	0.257038259	0.985994892	symptomatic
**Attention Deficit Hyperactivity disorder**	guanfacine	-0.111252342	0.257038259	0.985994892	FDA approved
**Attention Deficit Hyperactivity disorder**	imipramine	0.139539272	0.054347803	0.827224118	symptomatic
**Autism spectrum disorder**	risperidone	-0.134378364	0.219390105	0.985972983	symptomatic
**Autism spectrum disorder**	risperidone	-0.134378364	0.219390105	0.985972983	FDA approved
**Bone mineral density, femoral**	colecalciferol	-0.188639705	0.049803661	0.76298072	disease modifying
**Bone mineral density, femoral**	hydrochlorothiazide	0.153774546	0.169041741	0.863285507	non-indication

**Bone mineral density, forearm**	etidronic acid	-0.216329549	0.252813311	0.951583779	disease modifying
**Bone mineral density, forearm**	hydrochlorothiazide	0.304586861	0.022901794	0.76867816	non-indication
**Bone mineral density, forearm**	noretynodrel	0.312731504	0.015896842	0.687755769	non-indication
**Bone mineral density, forearm**	raloxifene	-0.276324894	0.056777236	0.951583779	disease modifying
**Bone mineral density, spine**	noretynodrel	0.158866666	0.157522538	0.993384055	non-indication
**Coronary artery disease**	estradiol	-0.103084548	0.085064907	1	non-indication
**Coronary artery disease**	fenofibrate	-0.100479296	0.104570026	1	disease modifying
**Coronary artery disease**	nifedipine	-0.093038538	0.173487447	1	symptomatic
**Crohn's disease**	atropine	-0.118239687	0.028824267	0.997883623	symptomatic
**Crohn's disease**	mesalazine	-0.090796677	0.219120371	0.997883623	disease modifying
**Depressive symptoms**	citalopram	0.218833452	0.132376543	0.999155236	symptomatic
**Depressive symptoms**	citalopram	0.218833452	0.132376543	0.999155236	FDA approved
**Depressive symptoms**	imipramine	0.248230269	0.053679776	0.999155236	symptomatic
**Depressive symptoms**	isocarboxazid	0.252550663	0.045764743	0.999155236	FDA approved
**Depressive symptoms**	paroxetine	0.2956409	0.006239812	0.624596786	symptomatic
**Diabetes, type 2**	orlistat	0.154929852	0.171204983	0.966436519	disease modifying
**Diabetes, type 2**	simvastatin	0.189709207	0.035603451	0.690209389	non-indication

**Glucose, 2hr tolerance test**	bromocriptine	0.209214792	0.174810875	0.995676207	disease modifying
**Glucose, 2hr tolerance test**	ramipril	0.21935404	0.138012423	0.995676207	disease modifying
**Kidney, chronic disease**	bumetanide	-0.177695054	0.12822103	0.999656561	symptomatic
**Kidney, chronic disease**	deferoxamine	0.155198489	0.264358965	0.999656561	symptomatic
**Kidney, chronic disease**	furosemide	0.201468945	0.04484184	0.838542408	symptomatic
**Kidney, eGFR**	deferoxamine	0.10441957	0.007242401	0.375082072	symptomatic
**Kidney, eGFR**	etacrynic acid	-0.077462044	0.184456507	0.998475555	symptomatic
**Myocardial infarction**	nadolol	0.132228105	0.051313062	1	FDA approved
**Myocardial infarction**	spironolactone	0.146318027	0.015107966	0.689244639	FDA approved
**Rheumatoid arthritis**	azathioprine	-0.104201393	0.20677519	0.995126362	disease modifying
**Rheumatoid arthritis**	azathioprine	-0.104201393	0.20677519	0.995126362	FDA approved
**Rheumatoid arthritis**	betamethasone	-0.138806481	0.025082491	0.995126362	disease modifying
**Rheumatoid arthritis**	cyclobenzaprine	0.097368356	0.272097325	0.995126362	non-indication
**Rheumatoid arthritis**	dexamethasone	-0.106127135	0.191440792	0.995126362	disease modifying
**Rheumatoid arthritis**	diclofenac	0.121676324	0.069567319	0.995126362	symptomatic
**Rheumatoid arthritis**	ergocalciferol	-0.119298868	0.100113866	0.995126362	disease modifying
**Rheumatoid arthritis**	methylprednisolone	-0.104845819	0.201608326	0.995126362	disease modifying
**Rheumatoid arthritis**	methylprednisolone	-0.104845819	0.201608326	0.995126362	FDA approved
**Rheumatoid arthritis**	prednisone	-0.104361882	0.20548538	0.995126362	disease modifying
**Rheumatoid arthritis**	prednisone	-0.104361882	0.20548538	0.995126362	FDA approved
**Rheumatoid arthritis**	tolmetin	-0.101840621	0.225942544	0.995126362	symptomatic
**Schizophrenia**	carbamazepine	0.065126629	0.099080856	0.983605046	symptomatic
**Schizophrenia**	clozapine	-0.05783003	0.196868911	0.983605046	symptomatic
**Schizophrenia**	clozapine	-0.05783003	0.196868911	0.983605046	FDA approved

**Schizophrenia**	dimenhydrinate	0.058083355	0.197454694	0.983605046	symptomatic
**Schizophrenia**	fluphenazine	-0.070282181	0.058430042	0.983605046	symptomatic
**Schizophrenia**	haloperidol	-0.057064939	0.207931914	0.983605046	symptomatic
**Schizophrenia**	haloperidol	-0.057064939	0.207931914	0.983605046	FDA approved
**Systemic lupus erythematosus**	betamethasone	-0.115183595	0.135556012	0.861368542	disease modifying
**Systemic lupus erythematosus**	cyclobenzaprine	-0.102318056	0.233944629	0.930409689	non-indication
**Systemic lupus erythematosus**	hydrocortisone	0.194276372	0.000137914	0.090264554	disease modifying
**Systemic lupus erythematosus**	hydrocortisone	0.194276372	0.000137914	0.090264554	FDA approved
**Systemic lupus erythematosus**	methotrexate	-0.114988865	0.136871396	0.861368542	disease modifying
**Systemic lupus erythematosus**	methylprednisolone	-0.112815623	0.15198953	0.880047148	disease modifying
**Systemic lupus erythematosus**	methylprednisolone	-0.112815623	0.15198953	0.880047148	FDA approved
**Systemic lupus erythematosus**	omeprazole	0.132343339	0.072544811	0.730470442	non-indication
**Total cholesterol**	lovastatin	-0.080131419	0.130660265	0.999184816	FDA approved

Supplementary References

1. Gamazon, E. R. *et al.* A gene-based association method for mapping traits using reference transcriptome data. *Nat. Genet.* **47,** 1091–1098 (2015).
2. Li, C. & Li, H. Network-constrained regularization and variable selection for analysis of genomic data. *Bioinformatics* **24,** 1175–1182 (2008).
3. Zhang, W. *et al.* Molecular pathway identification using biological network-regularized logistic models. *BMC Genomics* **14,** S7 (2013).
4. Shabalin, A. A. Matrix eQTL: Ultra fast eQTL analysis via large matrix operations. *Bioinformatics* **28,** 1353–1358 (2012).
5. Li, Y. I. *et al.* RNA splicing is a primary link between genetic variation and disease. *Science* **352,** 600–4 (2016).
6. Ernst, J. & Kellis, M. ChromHMM: automating chromatin-state discovery and characterization. *Nat. Methods* (2012). doi:10.1038/nmeth.1906
7. Su, Z., Marchini, J. & Donnelly, P. HAPGEN2: Simulation of multiple disease SNPs. *Bioinformatics* **27,** 2304–2305 (2011).
8. Auton, A. *et al.* A global reference for human genetic variation. *Nature* **526,** 68–74 (2015).
9. Zhou, X. & Stephens, M. Efficient multivariate linear mixed model algorithms for genome-wide association studies. *Nat. Methods* (2014). doi:10.1038/nmeth.2848
10. Zeng, P. & Zhou, X. Non-parametric genetic prediction of complex traits with latent Dirichlet process regression models. *Nat. Commun.* (2017). doi:10.1038/s41467-017-00470-2
11. Landrum, M. J. *et al.* ClinVar: improving access to variant interpretations and supporting evidence. *Nucleic Acids Res.* **46,** D1062–D1067 (2018).
12. McKusick-Nathans Institute of Genetic Medicine, Johns Hopkins University (Baltimore, M. Online Mendelian Inheritance in Man, OMIM®. Available at: https://omim.org/. (Accessed: 1st February 2018)
13. Wang, L. *et al.* SoftPanel: A website for grouping diseases and related disorders for generation of customized panels. *BMC Bioinformatics* **17,** 1–9 (2016).
14. Blake, J. A. *et al.* Mouse Genome Database (MGD)-2017: Community knowledge resource for the laboratory mouse. *Nucleic Acids Res.* **45,** D723–D729 (2017).
15. Lek, M. *et al.* Analysis of protein-coding genetic variation in 60,706 humans. *Nature* **536,** 285–91 (2016).
16. Himmelstein, Daniel; Khankhanian, Pouya; S. Hessler, Christine; J. Green, Ari; Baranzini, S. *PharmacotherapyDB 1.0: the open catalog of drug therapies for disease.* (2016).

doi:10.6084/m9.figshare.3103054.v1

17. Wei, W. Q. *et al.* Development and evaluation of an ensemble resource linking medications to their indications. *J. Am. Med. Informatics Assoc.* (2013). doi:10.1136/amiajnl-2012-001431
18. Khare, R., Li, J. & Lu, Z. LabeledIn: Cataloging labeled indications for human drugs. *J. Biomed. Inform.* (2014). doi:10.1016/j.jbi.2014.08.004
19. Khare, R. *et al.* Scaling drug indication curation through crowdsourcing. *Database* (2015). doi:10.1093/database/bav016
20. McCoy, A. B. *et al.* Development and evaluation of a crowdsourcing methodology for knowledge base construction: Identifying relationships between clinical problems and medications. *J. Am. Med. Informatics Assoc.* (2012). doi:10.1136/amiajnl-2012-000852
21. Gottlieb, A., Stein, G. Y., Ruppin, E. & Sharan, R. PREDICT: A method for inferring novel drug indications with application to personalized medicine. *Mol. Syst. Biol.* (2011). doi:10.1038/msb.2011.26
22. Fromer, M. *et al.* Gene expression elucidates functional impact of polygenic risk for schizophrenia. *Nat. Neurosci.* **19,** 1442–1453 (2016).
23. Barbeira, A. N. *et al.* Exploring the phenotypic consequences of tissue specific gene expression variation inferred from GWAS summary statistics. *Nat. Commun.* **9,** 1–20 (2018).
24. Hauberg, M. E. *et al.* Large-Scale Identification of Common Trait and Disease Variants Affecting Gene Expression. *Am. J. Hum. Genet.* **100,** 885–894 (2017).
25. So, H. C. *et al.* Analysis of genome-wide association data highlights candidates for drug repositioning in psychiatry. *Nat. Neurosci.* **20,** 1342–1349 (2017).
26. Zou, H. & Hastie, T. Regularization and variable selection via the elastic net. *J. R. Stat. Soc. Ser. B (Statistical Methodol.* **67,** 301–320 (2005).
27. de Leeuw, C. A., Mooij, J. M., Heskes, T. & Posthuma, D. MAGMA: Generalized Gene-Set Analysis of GWAS Data. *PLoS Comput. Biol.* **11,** 1–19 (2015).
28. Agresti, A. *An Introduction to Categorical Data Analysis: Second Edition*. (2006). doi:10.1002/0470114754
