# Supplementary material for: Integrative transcriptome imputation reveals tissue-specific and shared biological mechanisms mediating susceptibility to complex traits
Source: Nat Commun. 2019 Aug 23;10:3834. doi: 10.1038/s41467-019-11874-7 (PMC6707297; doi:10.1038/s41467-019-11874-7)
Supplement: Supplementary file 13 — Description of Additional Supplementary Files [file 41467_2019_11874_MOESM13_ESM.docx]

**Description of Additional Supplementary Files**

**File name:** Supplementary Data 1

**Description:** Comparison of R^2^_CV_ between EpiXcan approach and PrediXcan. *p* values show significances of EpiXcan R^2^_CV_ improvements over PrediXcan R^2^_CV_ using Wilcoxon pair-wise test with ‘greater’ option.

**File name:** Supplementary Data 2

**Description:** Comparison of prediction correlations, R^2^_PP_, between EpiXcan and PrediXcan.

**File name:** Supplementary Data 3

**Description:** Information of the 58 traits and GWAS datasets. In total, there are 58 GWASs that considered. This table gives the resource and information of each GWAS used in our analysis. Corresponding full names of the traits are listed and the category to which each trait belongs is also provided. We give the number of genes that significantly associate with each trait. The numbers of genes up-/down- regulated with all of the traits are listed.

**File name:** Supplementary Data 4

**Description:** GSEA - pLI (excel file with 4 sheets). Gene set enrichment analysis (GSEA) for all pLI (probability of loss of function intolerant) deciles. We first test for enrichment among all significant genes identified by each method (sheets: “EpiXcan_all” and “PrediXcan_all” respectively). We then test for enrichment among genes specific to each trait categories identified by each method (sheets: “EpiXcan_trait_categories” and “PrediXcan_trait_categories” respectively”). GSEA is performed for all pLI deciles, *p* values are calculated with the fisher exact test and are FDR-adjusted to q values.

**File name:** Supplementary Data 5

**Description:** Pathway analysis of significantly associated genes in 43 traits. In order to study if the associated genes are specific to biological processes regarding various diseases, we performed gene-set enrichment analysis of 43 traits with >10 significantly associated genes. After corrections, 74 highly enriched pathways are obtained with *p* value < 1.70$\times$10^-5^, and adjusted *p* value < 0.0488. This table provides the top pathways in which the associated genes are enriched.

**File name:** Supplementary Data 6

**Description:** Computational drug repurposing hits for traits, chemogenomic enrichment and indication enrichment analyses.

**File name:** Supplementary Data 7

**Description:** Trait association pairs. For each pair of traits, we calculate the number of significantly associated genes that they share. Only traits that associated with at least 50 genes are considered. The number of significantly associated genes of each trait is listed. In total, there are 311 association pairs, 245 of which are crossing trait categories. 66 association pairs are within trait categories.

**File name:** Supplementary Data 8

**Description:** qtlBHM priors of each dataset. This table lists the prior values of SNPs within every annotation region that integrated.

**File name:** Supplementary Data 9

**Description:** The names of the 1,309 perturbagens and prototype-ranked list (PRL) signatures. Each column represents a single perturbagen, individual cells are comprised of entrez gene identifiers, which are ordered according to their PRL assigned rank, with identifiers at the top representing genes that are over-expressed following drug treatment, and those at the bottom of the list representing those that are relatively under-expressed.

**File name:** Supplementary Data 10

**Description:** Query parameters for traits in the study used to a) infer gene-trait associations from the clinical datasets and b) identify trait-compound pairs for clinical indications.
