# Supplementary material for: Integrative transcriptome imputation reveals tissue-specific and shared biological mechanisms mediating susceptibility to complex traits
Source: Nat Commun. 2019 Aug 23;10:3834. doi: 10.1038/s41467-019-11874-7 (PMC6707297; doi:10.1038/s41467-019-11874-7)
Supplement: Supplementary file 14 — Peer Review File [file 41467_2019_11874_MOESM14_ESM.pdf]

Editorial Note: This manuscript has been previously reviewed at another journal that is not operating a transparent peer review scheme. The manuscript was considered suitable for publication without further review at *Nature Communications.*
